# Highly diastereoselective cascade [5 + 1] double Michael reaction, a route for the synthesis of spiro(thio)oxindoles

**DOI:** 10.1038/s41598-021-01766-6

**Published:** 2021-11-24

**Authors:** Firouz Matloubi Moghaddam, Vahid Saberi, Ashkan Karimi

**Affiliations:** 1 Laboratory of Organic Synthesis and Natural Products, Department of Chemistry, Sharif University of Technology, Tehran, 111559516 Iran; 2 Department of Chemistry, McGill University, Montreal, H3A-0B8 Canada

**Keywords:** Biochemistry, Chemistry

## Abstract

The first diastereoselective synthesis of spirothiooxindoles is reported via the Michael reaction between thiooxindoles and dibenzalacetones. The reaction was conducted without any catalyst or additive under green conditions, i.e., ethanol as the solvent and at room temperature. In addition, the described robust method benefits from scalability, simple work-up, and column chromatography-free purification. This work demonstrates the art of governing regio- and stereoselectivity, which has been discussed in the light of Density Functional Theory calculations. Our method represents the first synthesis of spiro[cyclohexanone-thiooxindoles] with the relative configuration of the aryl moieties at the cyclohexanone ring as *cis*. The obtained *cis*-spirothiooxindoles, can be used to afford *cis*-spirooxindoles, which their synthesis had not been explored before. According to our molecular docking studies, *cis*-spirooxindoles demonstrate higher binding affinities than corresponding *trans*-spirooxindoles for the OPRT domain of the *Leishmania donovani* uridine 5′-monophosphate synthase (LdUMPS). Thus, the reported method may eventually be utilized to develop new hit compounds for leishmaniasis treatment.

## Introduction

**Figure 1:**
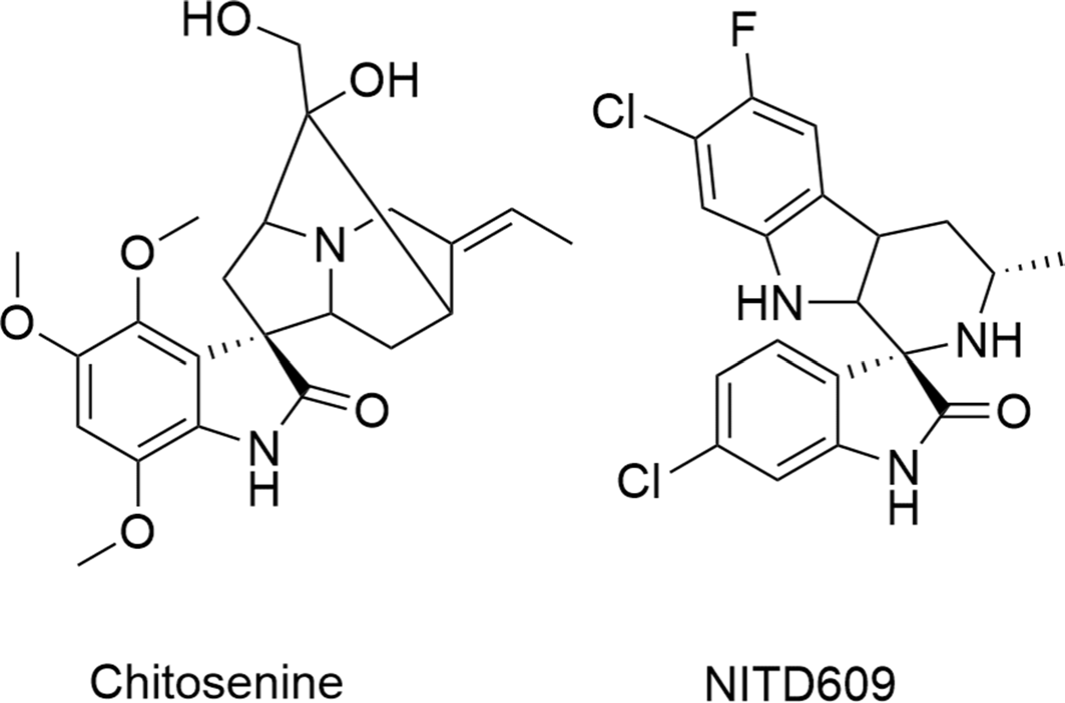
Biologically active natural products with a spirooxindole scaffold.

**Figure 2:**
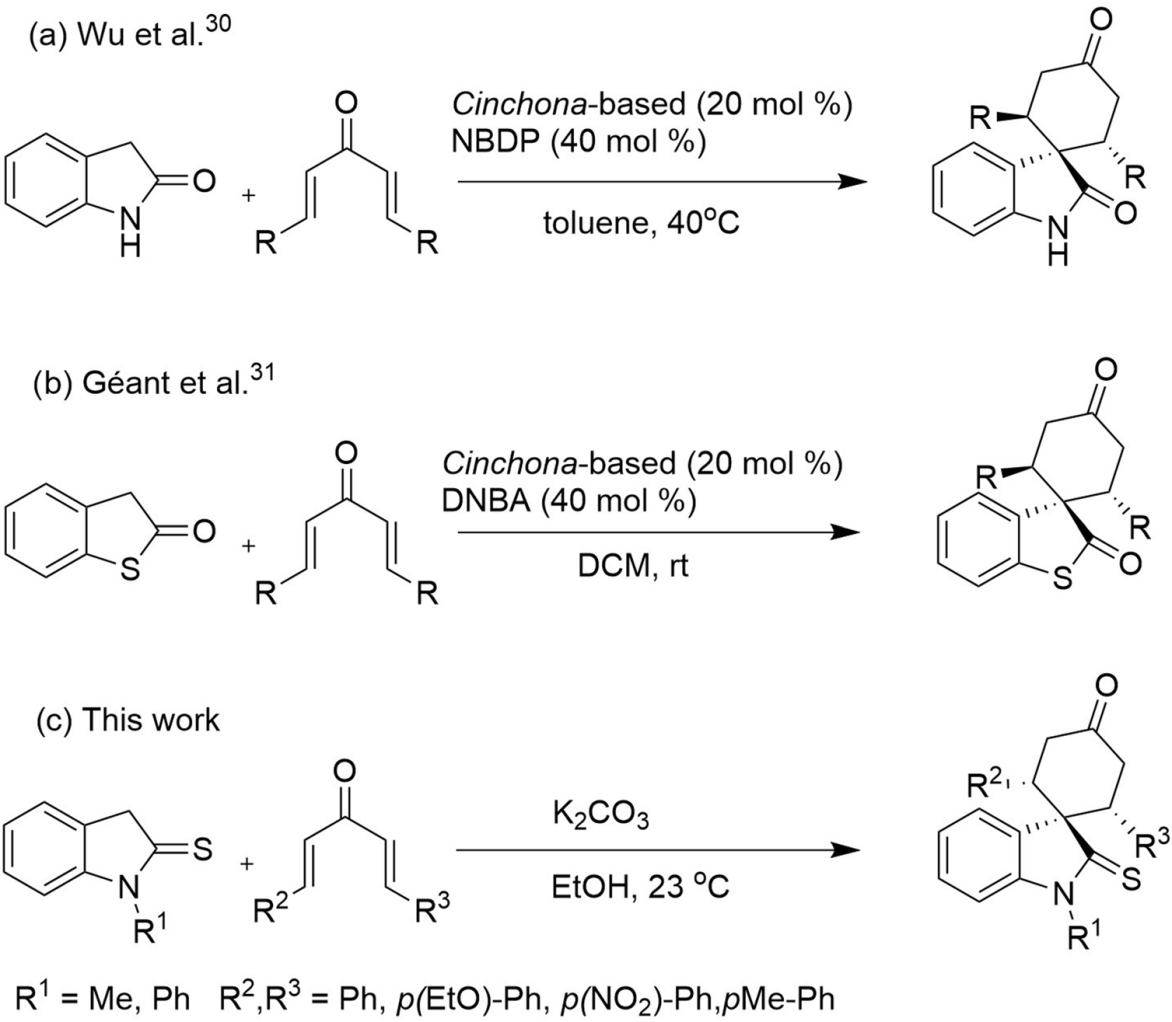
Comparison of different cascade [5 + 1] double Michael additions of dibenzalacetones on (thio)oxindoles.

Oxindole scaffolds are a vital pharmacophore^1^, encountered in natural products^2,3^ and pharmaceutical compounds. Inspired by the presence of spirooxindole scaffolds in several natural products such as gelsemine, chitosenine, spirotryprostatin B, and NITD609^4–11^ (Fig. 1), it is speculated that introducing a tetrahedral spiro carbon can further improve bioactivity by providing structural rigidity^12^. Consequently, there have been numerous efforts made towards synthesizing heterocycles containing spirooxindole as the core structure^13–18^. Among different methodologies developed for synthesizing spirocyclic compounds^19,20^, the Michael addition is one of the most robust^21–24^. Recently, the organocatalytic cascade Michael addition has emerged as an efficient strategy for synthesizing spirooxindoles in one-pot^25–29^. For example, Wu et al*.* demonstrated a cascade [5 + 1] double Michael addition between dibenzalacetones and oxindoles^30^ to afford spiro[cyclohexanone-oxindole] derivatives (Fig. 2a). Later, Géant et al*.* applied a tandem [5 + 1] double Michael addition, obtaining spiro[cyclohexanone-benzothiophenone] derivatives with high optical purity (Fig. 2b) ^31^.

However, despite all previous efforts to synthesize spiro[cycloalkanone-oxindole] compounds with the relative configuration of the aryl moieties at the cycloalkanone ring as *trans* (*trans*-spirooxindoles), the synthesis of *cis*-spirooxindoles has remained unexplored. Since previous biological studies have only focused on *trans*-spirooxindoles, the *cis* isomer could be a demanding target for synthesis as it may further expand the biological activities of oxindoles. For example, Scala et al*.* discovered *trans*-spiro[cyclohexanone-oxindole] derivatives are efficient inhibitors that kill *Leishmania infantum* promastigotes and amastigotes without significant cytotoxic effects^32^. More evidence for the high activity of spirooxindoles against leishmaniasis were confirmed by Saha et al.^33^. The leishmaniases are a group of diseases caused by protozoan parasites from more than 20 *Leishmania *species^34^. A better understanding of leishmaniasis was provided when French et al. demonstrated that *L. donovani* uridine 5′-monophosphate synthase (LdUMPS) is an essential enzyme for promastigotes viability and presented the crystal structure of the LdUMPS in complex with its product, UMP^35^.Their structural analysis revealed a tetrameric structure for LdUMPD with two dimeric OMPDC and two dimeric OPRT functional domains. As a result of this unusual structure, the oligomerization of LdUMPS is controlled by ligand binding at the OPRT active site.

Regarding the structure–activity relationship, we hypothesized a *cis*-spiro[cyclohexanone-(thio)oxindole] might have a higher affinity for the OPRT domain of LdUMPS than the *trans* isomer. To test this hypothesis, some docking studies were initially done, and then a method to synthesize *cis*-spirothiooxindoles was introduced (Fig. 2c). In the next step and in continuation of our previous works on oxindole chemistry^36,37^, thiooxidole was employed to react with dibenzalacetones. The highly diastereoselective [5 + 1] double Michael addition resulted in *cis*-spiro[cyclohexanone-thiooxindoles], which could then be converted to *cis*-spiro[cyclohexanone-oxindoles]. Finally, density functional theory (DFT) was used to explain the reaction regioselectivity and stereoselectivity.

## Result and discussion

**Figure 3:**
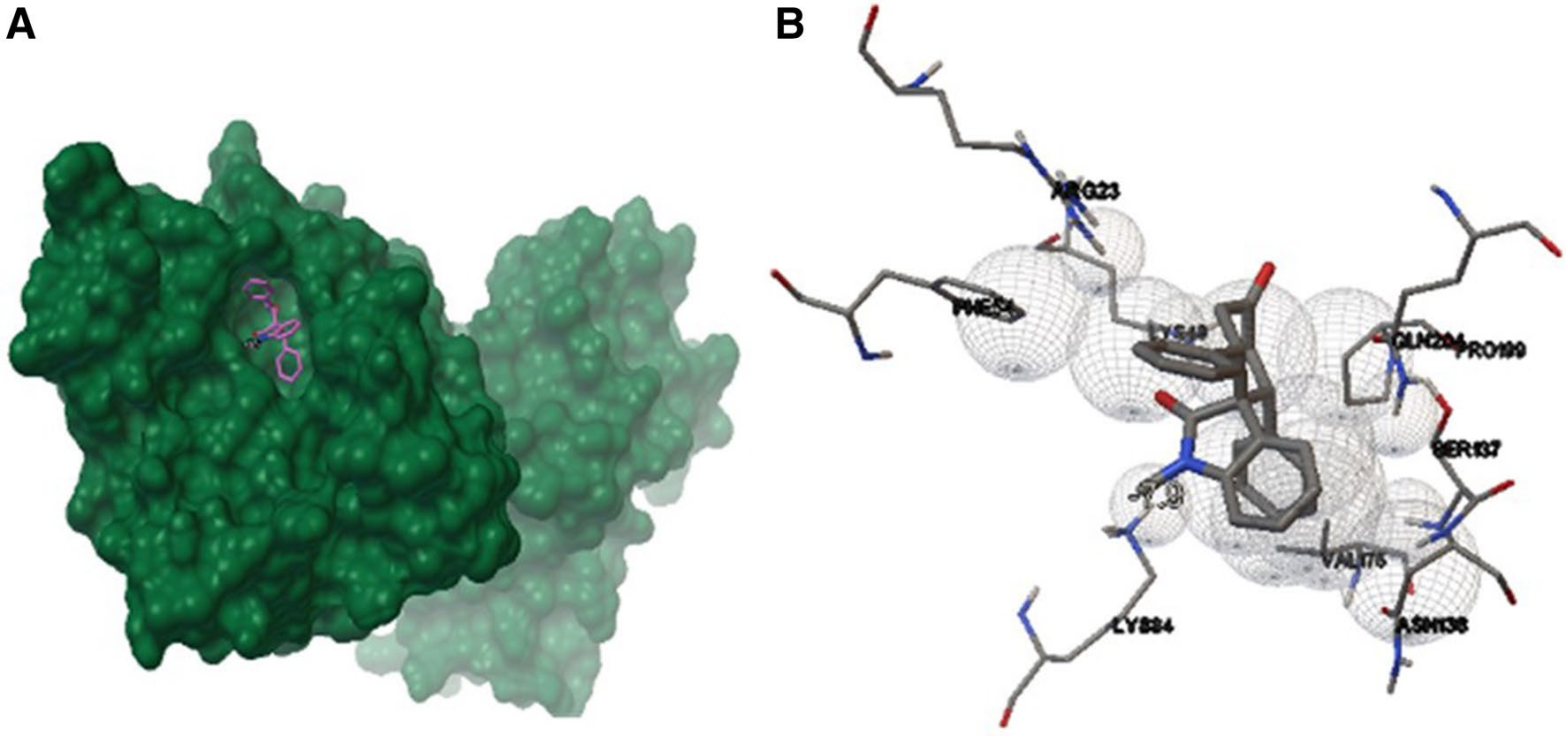
Interaction between *cis*-spirooxindole **2** with (**A**) OPRT domain of the LdUMPS (PDB ID: 2WNS) and (**B**) residual amino acids of the active site, which include Arg23, Phe54, Lys84, Asn138, Val175, Ser137, Pro199, Gln204, and Lys49. The figure was drawn using UCSF chimera 1.8 (https://www.cgl.ucsf.edu/chimera) and AutoDockTools version 1.5.6 (http://autodock.scripps.edu)^38,39^.

To gain insight into the molecular determinants that can modulate the antagonistic activities of oxindoles and thiooxindoles, we performed molecular docking studies on *cis* and *trans* isomers. The results generated based on the crystal structure of the LdUMPS receptor, revealed that *cis*-spiro[cyclohexanone-oxindole] **2** (*cis*-spirooxindole) has the highest binding affinity for the LdUMPS active site (Table 1). It binds to the OPRT domain of the LdUMPS receptor through interactions with Arg23, Phe54, Lys84, Asn138, Val175, Ser137, Pro199, Gln204, and Lys49 (Figs. 3 and S1). The higher binding energy of *cis*-spirooxindole **2** than the *trans* isomer **1** relies on steric hindrance between *trans*-spirooxindole and the active site. Electronic factors seem responsible for the higher activity of *cis*-oxindole **2** in comparison with *cis*-thiooxindole **4**.

**Table 1:**
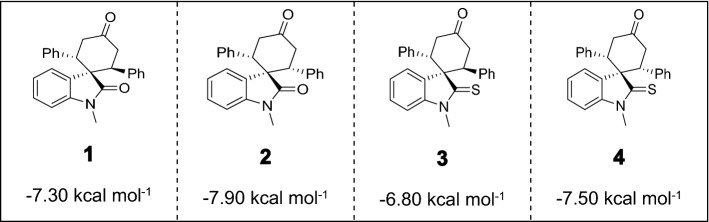
Calculated binding energies (kcal mol^−1^) of *trans/cis*-spirocyclic (thio)oxindoles with OPRT domain of LdUMPS.

**Figure 4:**
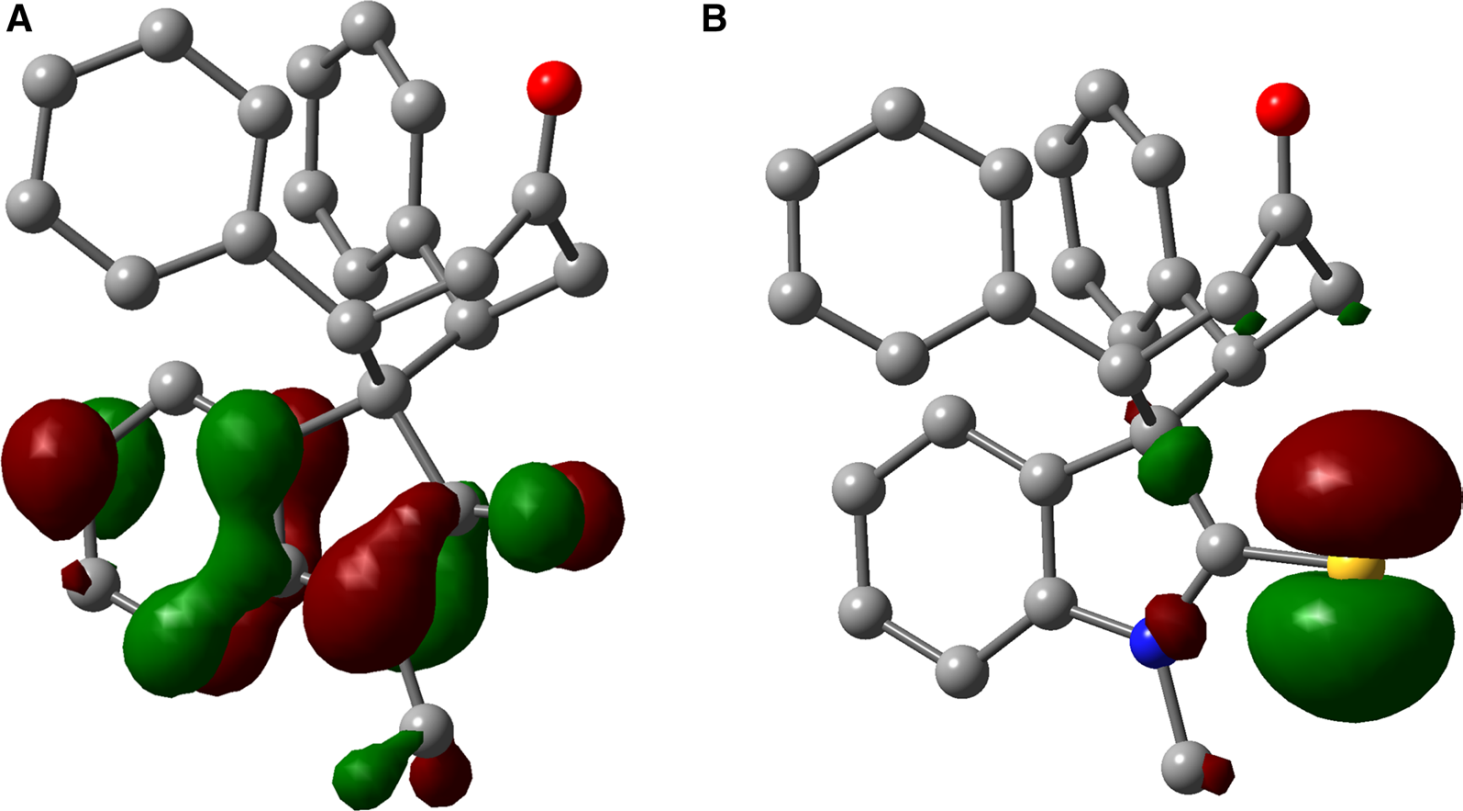
HOMO electronic cloud of (**A**) *cis*-spirooxindole **2** and (**B**) *cis*-spirothiooxindole **4**. The diffusive electronic cloud around the sulfur atom in thiooxindole makes the *cis* isomer to be more stable than the *trans* isomer. DFT calculations were performed using B3LYP/6-31 + G(d) in the gas phase. The figure was drawn using GaussView version 6.1.1 (https://gaussian.com/gaussview6)^40^.

Motivated by the initial docking results, which confirmed the importance of designing a robust synthetic method to provide *cis*-oxindole **2**, we, therefore, commenced the synthesis design. Since all previously applied Michael reactions on oxindole have thus far resulted in the *trans* isomer **1** formation, it was hypothesized that replacing the carbonyl group of the oxindole with a thiocarbonyl group may introduce an intramolecular repulsion between the large lone-pair electrons of sulfur and aryl π electrons. This could favor the formation of *cis*-thiooxindole **4,** which after one extra step could be converted to *cis*-oxindole **2**. Theoretical calculations confirmed this hypothesis. The DFT computations (Fig. 4) demonstrated that the *cis* isomer **4** is more thermodynamically stable than the *trans* isomer **3** (Table S1), due to the electronic repulsion. These results also explain why previous works on oxindoles, where the *trans* isomer **1** is more stable than the *cis* isomer **2**, could only achieve the *trans* isomer.

In the following step, thiooxindole derivatives were generated from the reaction of oxindole with P_2_S_5_ and sodium bicarbonate in dry THF. dibenzalacetone derivatives were prepared through the condensation reaction of acetone and aryl aldehydes in the presence of a suitable base with ethanol as the solvent. The [5 + 1] double Michael addition reaction was conducted with an equimolar mixture of freshly prepared thiooxindole **6** with dibenzalacetone to provide the single diastereomer **7** in high yield. The product is not soluble in the reaction solvent—ethanol—thus, we could isolate the pure product by filtration followed by washing it with ethanol and diethyl ether. The regio- and diastereoselectivity of this reaction were established by the aim of the one- and two-dimensional NMR. In ^1^H-NMR, compound **7** displayed two doublets at δ 6.55 and 6.65 ppm, equivalent to four protons for each signal. This confirmed the *p*-ethoxyphenyl groups of the thiooxindole moiety exist at the *cis* position with respect to each other. In the ^1^H and ^13^C-NMR spectra, no signal was observed related to other diastereomers. Further analysis by COSY, NOESY, and single-crystal X-ray diffraction, revealed that the aryl groups stand toward the phenyl part of thiooxindole.

**Figure 5:**
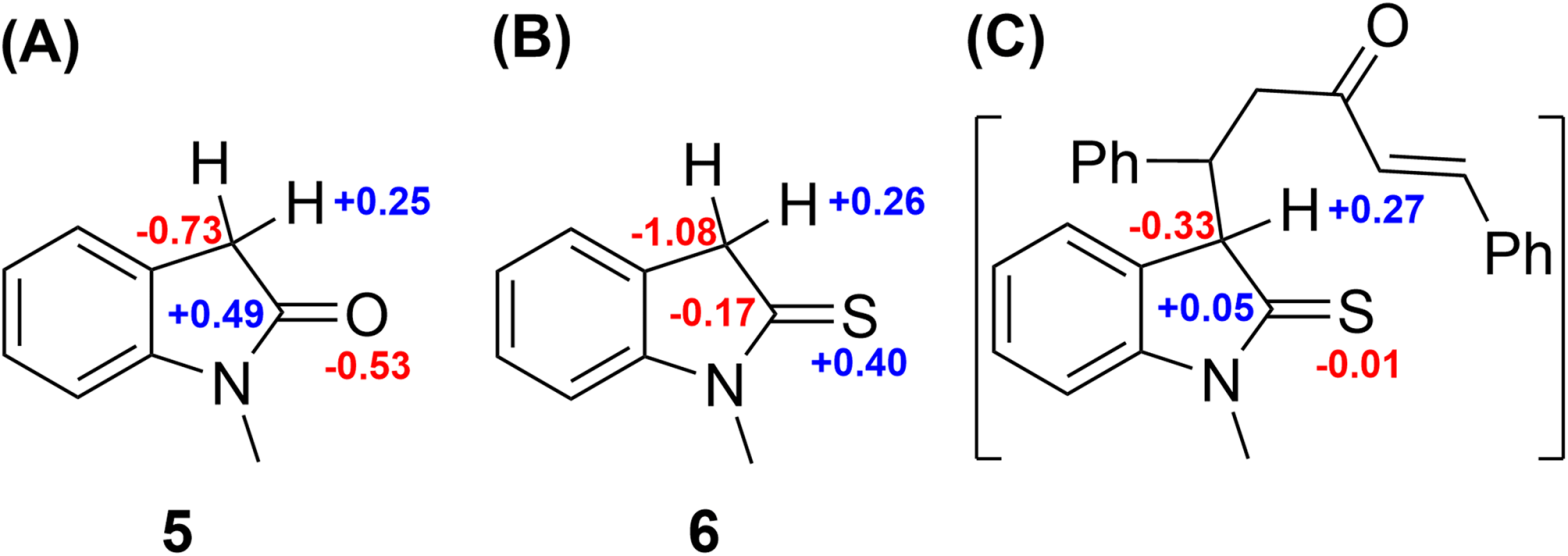
Mulliken charge distribution on (**A**) N-methyl oxindole **5**, (**B**) N-methyl thiooxindole **6** and (**C**) the intermediate after the first Michael addition on N-methyl thiooxindole **6**. Calculations were performed using B3LYP/6-31G(d) in the gas phase.

Initially, the obtained regioselectivity of this reaction was a surprise as one could expect to observe the Michael addition on alpha carbon and sulfur, which results in the formation of an eight-member ring. To explain the observed regioselectivity, we used DFT calculations. Considering the charge distribution (Fig. 5), we noticed the alpha carbon of thiooxindole is more electron-rich than sulfur—even after the first Michael addition (Fig. 5C)—and thus can act as a better nucleophile. Furthermore, the alpha carbon of thiooxindole **6** is more electron-rich than the alpha carbon of oxindole **5**. Accordingly, thiooxindole **6** is a better Michael donor than oxindole **5**. This trend is in agreement with the strong resonance between the nitrogen atom and the carbonyl group in oxindole, whereas in thiooxindole there is a stronger resonance between the alpha carbon and the thiocarbonyl group. Therefore, the charge distribution explains the formation of the spirocyclic scaffold over the eight-membered ring compounds.

Based on these results, we conducted various experiments to obtain the optimal reaction conditions using the reaction mentioned above as the model (Table 2). The results showed that K_2_CO_3_ accelerates the deprotonation step and thus makes the reaction proceed faster. The efficiency of this process could be further improved by increasing the amount of base up to 100 mol%. Moreover, exploring the effect of temperature showed that if the reaction was carried out in ethanol at a higher temperature (reflux condition), the yield of compound **7** would decrease. This temperature effect was predictable based on the negative entropy change for this reaction. We also studied the effect of L-Proline in the reaction, which resulted in a lower yield. Initially, it was expected that L-Proline would accelerate the reaction by forming dibenzaliminium intermediate. However, DFT calculations revealed that the beta carbon of dibenzaliminium is more electron-rich than the beta carbon of dibenzalacetone (Fig. S2), and thus the latter is a better Michael acceptor.

**Table 2:** Optimization of the double Michael addition reaction conditions.


Entry	Base	Solvent	Temperature (°C)	Yield^b^ (%)	Entry	Base	Solvent	Temperature (°C)	Yield^b^ (%)
1	K_2_CO_3_^c^	EtOH	23	21	7	K_2_CO_3_	Toluene^e^	23	31f.
2	K_2_CO_3_	EtOH	23	81	8	K_2_CO_3_	THF	23	Trace
3	K_2_CO_3_^d^	EtOH	23	56	9	K_2_CO_3_	H_2_O	23	Trace
4	–	EtOH	23	–	10	K_2_CO_3_	MeOH	23	Trace
5	DABCO	EtOH	23	20	11	K_2_CO_3_	DCM	23	Trace
6	NaOH	EtOH	23	Trace	12	K_2_CO_3_	EtOH	Reflux	36

^a^0.5 mmol thiooxindole (1.0 eq), 0.5 mmol dibenzalacetone (1.0 eq), 0.5 mmol base (1.0 eq), 20 mL solvent.

^b^Isolated yield.

^c^0.025 mmol base (0.5 eq) was used.

^d^0.5 mmol L-Proline (1.0 eq) was used as an organocatalyst.

^e^Mixture of *cis* and *trans* products was obtained.

^f^Isolated yield of the *cis* product.

**Figure 6:**
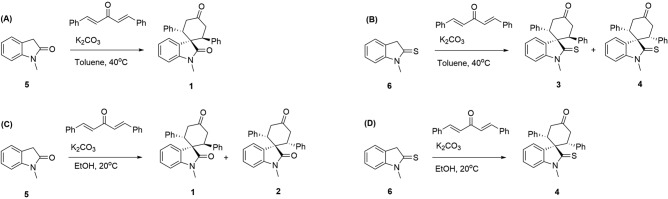
Double Michael addition reaction of N-methyloxindole and N-methylthiooxindole on dibenzalacetone in different solvents.

In the next step, the solvent effect on the reaction was surveyed. As an aprotic and apolar solvent, toluene has been previously used to convert oxindole **5** to *trans*-spirooxindole **1** (Fig. 6A). However, the same reaction condition on thiooxindole **6**, resulted in a mixture of *cis-* and *trans*-spirocyclic products **3** and **4** (Fig. 6B). We, therefore, introduced a method using ethanol as a polar and protic solvent that produces *cis*-spirothiooxindole **4** as a single diastereomer (Fig. 6D). Significantly, treating oxindole **5** under our reaction condition resulted in a mixture of *cis-* and *trans*-spirooxindole **1** and **2** (Fig. 6C). Therefore, we concluded both solvent and substrate electronic structure are involved in the determination of reaction diastereoselectivity. To better understand these effects, conductor-like polarizable continuum model (CPCM)^41^ was used in our DFT calculations. The theoretical results (Fig. S3) revealed *trans* configuration is more favorable in toluene, whereas the *cis* configuration can form easier in ethanol. This selectivity comes from the fact that the energy level of molecular orbitals and thus the repulsion between the chalcogen lone-pair electrons and π electrons of aryl groups depends on the solvent dielectric constant. Thus, the double Michael addition on oxindole (or thiooxindole) in ethanol (or toluene) resulted in selective formation of compound **1** (or **4**). However, when the reaction was performed on oxindole in toluene or on thiooxindole in ethanol, the solvent and electronic effects conflicted with each other, and a mixture of *cis* and *trans* isomers was obtained.

To further illustrate the scope of this reaction for the synthesis of complex heterocyclic spirothiooxindoles, N-methylthiooxindole and N-phenylthiooxindole with dibenzalacetone bearing electron-donating or -withdrawing groups were used. The obtained results are summarized in Table 3. In all cases, the reaction furnished a single diastereomer of spirothiooxindoles in moderate to good yields. Furthermore, we successfully scaled up the reaction and isolated > 35 g of compound **7** with 66% yield.

**Table 3:**
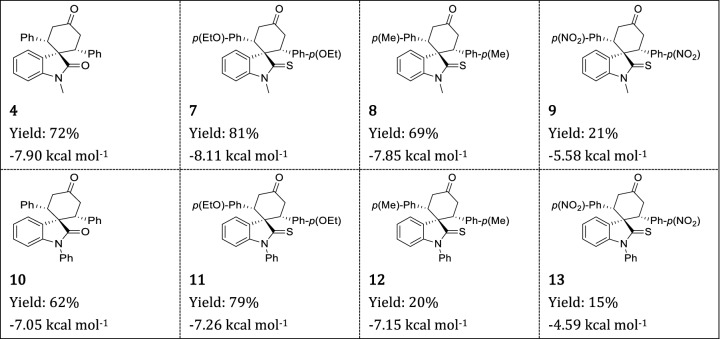
Double Michael addition of thiooxindole on dibenzalacetone, reaction yield, and binding energies (kcal mol^−1^) with OPRT domain of LdUMPS.

**Figure 7:**
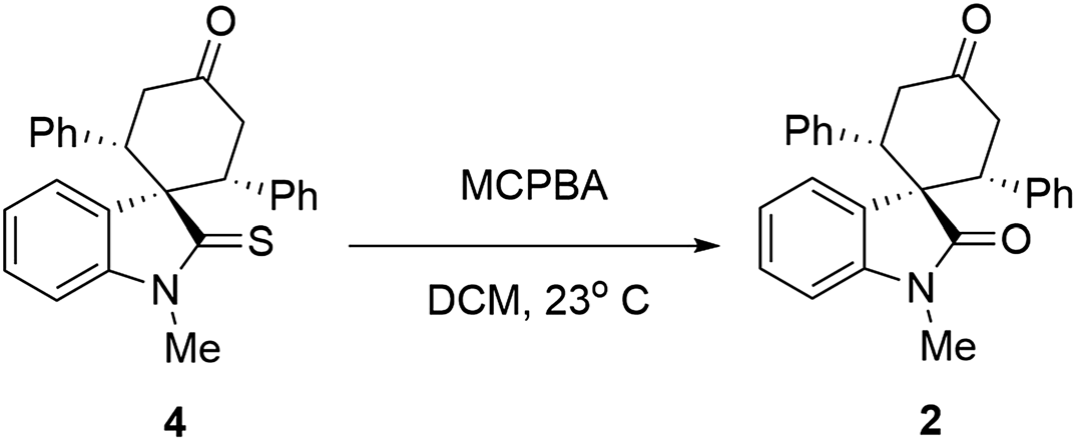
Conversion of *cis*-spirothiooxindole into *cis*-spirooxindole.

The potential of synthesized compounds as inhibitors of LdUMPS was then recognized by molecular docking (Table 3). Our study showed that compound **7** fits better into the active site of the receptor. N-phenyl derivatives sterically intercut this fitting, and, as a result, they have a lower binding affinity. Finally, we demonstrated that it is possible to convert synthesized spirocylic thiooxindoles into oxindoles (Fig. 7). Thus, our method opens a new window in the formation of biologically important *cis*-spirooxindoles and *cis*-spirothiooxindoles.

## Conclusion

In summary, we have introduced a robust synthetic strategy for diastereomerically-pure spiro[cyclohexanone-thiooxindole] compounds via double Michael addition of thiooxindoles on dibenzalacetone. The reaction furnished corresponding spiro products in only one diastereomer. The regioselectivity and stereoselectivity of the reaction were discussed in the light of the computational results. Molecular docking studies were performed on the reaction products, which demonstrated high binding affinities for the OPRT domain of the LdUMPS receptor. Our catalyst-free protocol is scalable, has mild reaction conditions, and does not need column chromatography for purification. These advantages make this method a suitable candidate for synthesizing complex spirocyclic oxindoles and thiooxindoles with potential applications in medicinal chemistry.

## Methods

### General information

All materials used are commercially available and were purchased from Merck and used without any additional purification. ^1^H NMR, ^13^C NMR, and 2-D NMR spectra were recorded on a Bruker (Avance DRX-500) spectrometer using CDCl_3_ or DMSO-d6 as solvent at room temperature. Chemical shifts (δ) are given in parts per million (ppm) and are reported relative to residual solvent peaks: CDCl_3_ (δH 7.26, δC 77.16 ppm), DMSO-d6 (δH 2.50, δC 39.52 ppm). Coupling constants (J) are given in Hertz (Hz). FTIR spectra of samples were taken using an ABB Bomem MB-100 FTIR spectrophotometer. HRMS analyses were performed using a TOF mass analyzer on Mat95XP-Trap apparatus. X-ray crystallography was carried out through APEX II CCD BRUKER detector.

### Computational studies

In all calculations, B3LYP DFT functional theory^42^ and 6-31G(d) basis set^43^ was used. Calculations were performed in the gas phase or solution by CPCM model and at 298.15 K. Conformer distribution was studied by Spartan04 (https://www.wavefun.com)^44^ and Gaussian16 revision A.03 (https://gaussian.com/gaussian16)^45^ was used for other calculations. The first frequency was utilized to assess whether structures were in the true optimized structure. The docking study was performed by using the Autodock 1.5.6. The size of the grid box was set to 20 Å. The enzyme structure was downloaded from the website protein data banks (PDB ID: 2WNS) and ligands optimized at 6-31G(d)-B3LYP level of theory by Gaussian. Auto Dock tools programs were used to visualize the complex in 3D. All single bonds of residue side chains inside the grid box were regarded as rotatable, and the docked ligand was allowed to rotate on all the single bonds and move flexibly within the grid box. The structural optimization was performed for 50,000 generations using a genetic algorithm.

### Single crystal X-ray diffraction

A single crystal of the compound **11** was mounted on a Kapton loop using a Paratone oil and analyzed by single crystal Xray diffraction. An APEX II CCD BRUKER detector and a graphite Mo-Kα monochromator were used for the data acquisition. All measurements were done at 150 K and a refinement method was used for solving the structure. The structure resolution was accomplished using the SHELXT-2014 program and the refinement was done with the SHELXL-2014/7 program^46,47^. The structure solution and the refinement were achieved with the PLATON software (http://www.platonsoft.nl/platon)^48^. Finally, pictures of the compound structure were obtained using the MERCURY 3.08 software (https://mercury1.software.informer.com)^49^. During the refinement steps, all atoms-except hydrogens-were refined anisotropically. The position of the hydrogens was determined using residual electronic densities which are calculated by a Fourier difference. Finally, in order to obtain a complete refinement, a weighting step followed by multiples loops of refinement was done. The R1 factor and the completeness are indicators of a complete refinement but also a good estimation of the quality of the experimental data.

### General procedure for the synthesis of dibenzalacetones

Preparation of dibenzalacetone from solid aryl aldehyde: A mixture of LiClO_4_ (5 mmol), Et_3_N (5 mmol), acetone (5 mmol), and aryl aldehyde (5 mmol) in 20 mL ethanol as solvent was stirred at room temperature. The reaction completion was monitored by TLC. After the completion of the reaction, the solvent was removed by rotatory evaporation at reduced pressure, and the residue was washed by ethanol to afford the pure product.

Preparation of dibenzalacetone from liquid aryl aldehyde: A mixture of NaOH (25 mmol), acetone (5 mmol), and aryl aldehyde (5 mmol) in the mixture of H_2_O/EtOH (10:8) was stirred at room temperature for 10 min. The reaction completion was monitored by TLC. After the completion of the reaction, the solvent was removed by rotatory evaporation at reduced pressure, and the residue was washed by ethanol to afford the pure product.
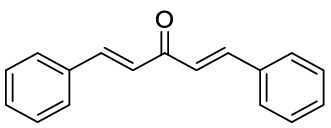


1*,5-Diphenyl-penta-1,4-dien-3-one:* Yellow solid, ^1^H NMR (CDCl_3_, 500 MHz), *δ* 7.02 (2 H, d, *J* = *15.9* Hz), 7.42 (4 H, dd, *J* = *5.0, 1.8* Hz), 7.63 (6 H, dd, *J* = *6.4, 3.1* Hz), 7.75 (2H, d, *J* = *15.9* Hz). HRMS (ESI): *m/z* 234.1051 (M^+**•**^ C_17_H_14_O^+**•**^ requires 234.1039). The spectroscopic data are consistent with those previously reported^50^.
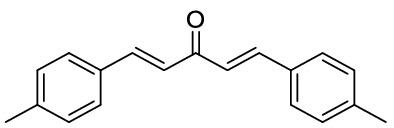


*1,5-Di-p-tolyl-penta-1,4-dien-3-one*: Yellow solid, ^1^H NMR (CDCl_3_, 500 MHz), δ 2.35 (6H, s), 7.09 (2H, d, *J* = 15.0 Hz), 7.29 (4H, d, *J* = 7.5 Hz, 2H), 7.50 (4H, d, *J* = 7.5 Hz), 7.92 (2H, d, *J* = 15.2 Hz). HRMS (ESI): *m/z* 262.1385 (M^+ **•**^ C_19_H_18_O^+**•**^ requires 262.1352). The spectroscopic data are consistent with those previously reported^50^.
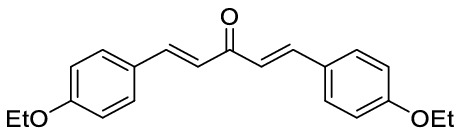


*1,5-Bis-(4-ethoxy-phenyl)-penta-1,4-dien-3-one:* Yellow solid, ^1^H NMR (CDCl_3_, 500 MHz), *δ* 1.44 (6 H, t, *J* = *7.0* Hz), 4.08 (4 H, q, *J* = *7.0* Hz), 6.92 (4 H, d, *J* = *8.7* Hz), 6.95 (2 H, d, *J* = *15.9* Hz), 7.56 (4 H, d, *J* = *8.7* Hz), 7.69 (2H, d, *J* = *15.8* Hz). HRMS (ESI): *m/z* 322.1574 (M^+ **•**^ C_21_H_22_O_3_^+**•**^ requires 322.1563). The spectroscopic data are consistent with those previously reported^50^.
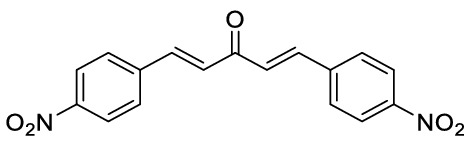


*1,5-Bis-(4-nitro-phenyl)-penta-1,4-dien-3-one:* Brown solid, ^1^H NMR (d6-DMSO, 500 MHz), *δ* 7.56 (2 H, d, *J* = *16.1* Hz), 7.93 (2 H, d, *J* = *16.1* Hz), 8.08 (4 H, d, *J* = *8.5* Hz), 8.31 (4H, d, *J* = *8.5* Hz). HRMS (ESI): *m/z* 324.0749 (M^+ **•**^ C_17_H_12_N_2_O_5_^+**•**^ requires 324.0746). The spectroscopic data are consistent with those previously reported^50^.

### General procedure for synthesizing thiooxindoles

An oven-dried flask was charged with oxindole (3.7 mmol), dry THF (25 mL), and P_2_S_5_ (2.3 mmol). The reaction mixture was stirred at room temperature for 10 min. Then, NaHCO_3_ (7.5 mmol) was slowly added and stirred overnight.

After removing the solvent by rotatory evaporation at reduced pressure, the residue was transferred into a 100-mL beaker. By adding 250 mL mixture of ice and water, a pale yellow solid appeared. The light-yellow precipitate was washed with 10% solution of NaHCO_3_ to remove unreacted oxindole and give the pure product.
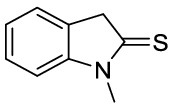


*1-methylindoline-2-thione (6):* Yellow solid, ^1^H NMR (CDCl_3_, 500 MHz), *δ* 3.61 (s, 3H), 4.09 (s, 2H), 6.98 (1H, d, *J* = 7.8 Hz), 7.16 (1H, t, *J* = 7.4 Hz), 7.27 (1H, d, *J* = 7.8 Hz), 7.32 (1H, t, *J* = 7.8 Hz). HRMS (ESI): *m/z* 163.0467 (M^+**•**^ C_9_H_9_NS^+**•**^ requires 163.0450). The spectroscopic data are consistent with those previously reported^51^.
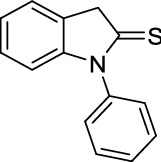


*1-phenylindoline-2-thione:* brown solid, ^1^H NMR (CDCl_3_, 500 MHz), *δ* 4.32 (2 H, s), 6.65 (1 H, d, *J* = *7.7* Hz), 7.20 (1 H, t, *J* = *7.32* Hz), 7.25 (1 H, t, *J* = *7.5* Hz), 7.38 (1 H, d, *J* = *7.15* Hz), 7.43 (2 H, d, *J* = *7.5* Hz), 7.55 (1 H, t, *J* = *7.4* Hz), 7.63 (2 H, t, *J* = *7.6* Hz). HRMS (ESI): *m/z* 225.0622 (M^+**•**^ C_14_H_11_NS^+**•**^ requires 225.0607). The spectroscopic data are consistent with those previously reported^52^.

### General procedure for double Michael addition reaction

A mixture of thiooxindole (0.5 mmol), dibenzalacetone (0.5 mmol), and K_2_CO_3_ (0.5 mmol) in 10 mL of ethanol was stirred at room temperature for about 24 h. Reaction completion was monitored by TLC using mobile phase (ethyl acetate/hexane; 1:6). Since the product is insoluble in ethanol and diethyl ether, the purification was done by washing the crude reaction mixture with these solvents.

*(1s,2R,6S)-1*′*-methyl-2,6-diphenyl-2*′*-thioxospiro[cyclohexane-1,3*′*-indolin]-4-one (4):* White solid, ^1^H NMR (CDCl_3_, 500 MHz), *δ* 2.82 (2H, dd, *J* = *15.7, 4.0* Hz), 3.00 (3 H, s), 3.46 (2 H, t, *J* = 15.1 Hz), 4.14 (2H, dd, *J* = 14.6, 4.2 Hz), 6.63 (1H, d, *J* = *6.9* Hz), 6.82 (4 H, d, *J* = *7.5* Hz), 6.95 (4 H, d, *J* = *7.7* Hz), 7.32–7.38 (2H, m), 8.02–8.07 (1H, m). ^13^C NMR (CDCl_3_, 125.8 MHz): *δ* 34.6, 43.7, 49.8, 67.6, 108.5, 125.5, 127.3, 127.5, 128.13, 128.5, 128.8, 129.5, 139.5, 146.9, 207.7, 208.5. HRMS (EI): *m/z* 397.1480 (M^+**•**^ C_26_H_23_NOS^+**•**^ requires 397.1495).

*(1s,2R,6S)-2,6-bis(4-ethoxyphenyl)-1*′*-methyl-2*′*-thioxospiro[cyclohexane-1,3*′*-indolin]-4-one (7):* White solid, ^1^H NMR (CDCl_3_, 500 MHz), *δ* 1.30 (6 H, t, *J* = *7.0* Hz), 2.75 (2H, dd, *J* = *15.6, 3.9* Hz), 3.00 (3 H, s), 3.39 (2 H, t, *J* = 15.1 Hz), 3.85 (4 H, q, *J* = *7.0* Hz), 4.06 (2H, dd, *J* = 14.3, 2.7 Hz), 6.45 (4 H, d, *J* = *8.7* Hz), 6.64 (1H, d, *J* = *6.9* Hz), 6.71 (4 H, d, *J* = *8.7* Hz), 7.32 (2H, quin, *J* = *7.6* Hz), 7.99 (1H, d, *J* = *7.3* Hz). ^13^C NMR (CDCl_3_, 125.8 MHz): *δ* 14.7, 31.1, 45.0, 51.2, 63.1, 68.1, 110.0, 113.3, 123.2, 125.3, 128.8, 129.5, 129.7, 132.6, 146.2, 157.8, 204.7, 208.2. HRMS (EI): *m/z* 485.2015 (M^+**•**^ C_30_H_31_NO_3_S^+**•**^ requires 485.2025).

*(1s,2R,6S)-1*′*-methyl-2*′*-thioxo-2,6-di-p-tolylspiro[cyclohexane-1,3*′*-indolin]-4-one (8):* White solid, ^1^H NMR (CDCl_3_, 500 MHz), *δ* 2.13 (6 H, s), 2.77 (2H, dd, *J* = *15.6, 4.1* Hz), 2.98 (3 H, s), 3.42 (2 H, t, *J* = 15.2 Hz), 4.09 (2H, dd, *J* = 14.5, 4.2 Hz), 6.62 (1H, d, *J* = *6.1* Hz), 6.68 (4 H, d, *J* = *8.0* Hz), 6.74 (4 H, d, *J* = *7.9* Hz), 7.31–7.41 (2H, m), 8.01 (1H, d, *J* = *7.2* Hz). ^13^C NMR (CDCl_3_, 125.8 MHz): *δ* 20.8, 31.0, 44.9, 51.7, 67.8, 110.0, 123.2, 125.3, 128.1, 128.3, 128.7, 132.5, 134.6, 136.6, 146.2, 204.5, 208.1. HRMS (EI): *m/z* 425.1796 (M^+**•**^ C_28_H_27_NOS^+**•**^ requires 425.1813).

*(1s,2R,6S)-1*′*-methyl-2,6-bis(4-nitrophenyl)-2*′*-thioxospiro[cyclohexane-1,3*′*-indolin]-4-one (9):* Brown solid, ^1^H NMR (CDCl_3_, 500 MHz) δ 2.81 (2H, dd, *J* = 15.6, 3.2 Hz), 3.00 (3H, s), 3.47 (2H, t, *J* = 15.2 Hz), 4.13 (2H, dd, *J* = 14.5, 3.5 Hz), 6.76 (1H, d, J = 6.3 Hz), 7.59–7.58 (2H, m), 7.68 (4H, d, *J* = *8.5* Hz), 7.91 (4H, d, *J* = *8.5* Hz) 8.16 (1H, dd, *J* = 4.9, 1.9 Hz). ^13^C NMR (CDCl_3_, 125.8 MHz): *δ* 34.6, 43.7, 49.8, 67.6, 108.5, 123.3, 125.5, 127.3, 128.5, 128.6, 129.5, 146.0 146.9, 149.0, 207.7, 208.5. HRMS (EI): *m/z* 487.1187 (M^+**•**^ C_26_H_21_N_3_O_5_S^+**•**^ requires 487.1202).

*(1s,2R,6S)-1*′*,2,6-triphenyl-2*′*-thioxospiro[cyclohexane-1,3*′*-indolin]-4-one (10):* White solid, ^1^H NMR (CDCl_3_, 500 MHz), *δ* 2.85 (2H, d, *J* = *14.5* Hz), 3.50 (2 H, t, *J* = *15.0* Hz), 4.16 (2H, d, *J* = *14.1* Hz), 6.20 (3H, s), 7.32 (8 H, s), 7.53–7.56 (11H, m), 8.06 (1H, d, *J* = *7.3* Hz). ^13^C NMR (CDCl_3_ and d6-DMSO, 125.8 MHz): *δ* 43.3, 50.5, 66.6, 109.5, 112.5, 122.3, 125.8, 126.7, 127.3, 127.4, 127.4, 128.3, 130.6, 133.3, 135.1, 135.3, 145.6, 204.4, 208.4. HRMS (EI): *m/z* 459.1553 (M^+ **•**^ C_31_H_25_NOS^+**•**^ requires 459.1657).

*(1s,2R,6S)-2,6-bis(4-ethoxyphenyl)-1*′*-phenyl-2*′*-thioxospiro[cyclohexane-1,3*′*-indolin]-4-one (11):* White solid, ^1^H NMR (CDCl_3_, 500 MHz), *δ* 1.34 (6H, s), 2.83 (2 H, d, *J 14.7*), 3.48 (2 H, t, *J* = *15.0*), 3.91 (4 H, s), 4.14 (2 H, d, *J* = *13.3*), 6.19 (1H, d), 6.28 (2 H, s), 6.54 (4 H, d, *J* = *7.6*), 6.81 (4 H, d, *J* = *7.4*), 7.21 (1 H, t), 7.33 (4 H, s), 8.04 (1 H, d,* J* = *6.6*). ^13^C NMR (CDCl_3_, 125.8 MHz): *δ* 13.5, 43.5, 50.0, 61.8, 67.0, 109.5, 112.0, 122.4, 124.6, 125.9, 127.5, 127.7, 128.3, 128.4, 128.5, 130.7, 135.1, 145.6, 156.7, 204.7, 208.6. HRMS (EI): *m/z* 547.2187 (M^+**•**^ C_35_H_33_NO_3_S^+**•**^ requires 547.2176).

*(1s,2R,6S)-1*′*-phenyl-2*′*-thioxo-2,6-di-p-tolylspiro[cyclohexane-1,3*′*-indolin]-4-one (12):* White solid, ^1^H NMR (CDCl_3_, 500 MHz), *δ* 2.23 (6H, s), 2.88 (2 H, d, *J* = *14.5* Hz), 3.53 (2H, t, *J* = *14.7* Hz), 4.18 (2H, d, *J* = 14.2 Hz), 6.23 (3H, s), 6.83 (8 H, dd, *J* = *25.0* Hz), 7.34 (5H, m), 8.08 (1H, d, *J* = *7.2* Hz). ^13^C NMR (CDCl_3_ and d6-DMSO, 125.8 MHz): *δ* 20.3, 44.0, 51.2, 67.3, 110.2, 113.2, 123.0, 126.5, 127.1, 128.0, 128.1, 128.3 129.0, 134.0, 135.8, 136.0, 146.4, 205.2, 207.1. HRMS (EI): *m/z* 487.1959 (M^+ **•**^ C_35_H_33_NO_3_S^+**•**^ requires 487.1970).

*(1s,2R,6S)-2,6-bis(4-nitrophenyl)-1*′*-phenyl-2*′*-thioxospiro[cyclohexane-1,3*′*-indolin]-4-one (13):* Brown solid, ^1^H NMR (CDCl_3_, 500 MHz) δ 2.95 (2H, dd, *J* = 15.5, 3.2 Hz), 3.60 (2H, t, *J* = 15.3 Hz), 4.26 (2H, dd, *J* = 14.5, 3.3 Hz), 6.72 (3H, s), 7.78–7.89 (5H, m), 7.98 (4H, d, *J* = *8.5* Hz), 8.01 (4H, d, *J* = *8.5* Hz) 8.15 (1H, dd, *J* = 4.4, 1.7 Hz). ^13^C NMR (CDCl_3_, 125.8 MHz): *δ* 43.7, 49.8, 67.3, 112.7, 123.3, 125.4, 126.4, 127.4, 128.2, 128.6, 128.6, 129.1, 131.3, 139.4, 142.6, 146.0 149.0, 207.0, 208.5. HRMS (EI): *m/z* 549.1361 (M^+**•**^ C_31_H_23_N_3_O_5_S^+**•**^ requires 549.1358).

## Supplementary Information

Supplementary Information 1.

## References

[1] Bassyouni FH, Hefnawi ME, Rashed AE, Rehim MA (2017). Molecular modeling and biological activities of new potent antimicrobial, antiinflammatory and anti-nociceptive of 5-nitro indoline-2-one derivatives. Drug Des..

[2] Brahmachari G, Banerjee B (2014). Facile and one-pot access of 3,3-bis(indol-3-yl)indolin-2-ones and 2,2-bis(indol-3-yl)acenaphthylen-1(2h)-one derivatives via an eco-friendly pseudo-multicomponent reaction at room temperature using sulfamic acid as an organo-catalyst. ACS Sustain. Chem. Eng..

[3] Venkatesan H, Davis MC, Altas Y, Snyder JP, Liotta DC (2001). Total synthesis of SR 121463 A, a highly potent and selective vasopressin v2 receptor antagonist. J. Org. Chem..

[4] Sakai S (1975). Gardneria alkaloids, part 9. Structures of chitosenine and three other minor bases: From gardneria multiflora makino. Tetrahedron Lett..

[5] Sakai S-I, Aimi N, Yamaguchi K, Yamanaka E, Haginiwa J (1982). Gardneria alkaloids. Part 13. Structure of gardmultine and demethoxygardmultine; bis-type indole alkaloids of gardneria multiflora makino. J. Chem. Soc., Perkin Trans..

[6] Rottmann M (2010). Spiroindolones, a potent compound class for the treatment of malaria. Science.

[7] Dideberg O (1977). Structure cristalline et moléculaire d'un nouvel alcalöide bisindolique: complexe moléculaire 1:2 strychnofoline–ethanol (C_30_H_34_N_4_O_2_.2C_2_H_6_O). Acta Cryst..

[8] Subramaniam G (2007). Biologically active aspidofractinine, rhazinilam, akuammiline, and vincorine alkaloids from kopsia. J. Nat. Prod..

[9] Janin YL (2007). Antituberculosis drugs: Ten years of research. Biorg. Med. Chem..

[10] Gutierrez-Lugo M-T, Bewley CA (2008). Natural products, small molecules, and genetics in tuberculosis drug development. J. Med. Chem..

[11] Zea A, Alba A-NR, Mazzanti A, Moyano A, Rios R (2011). Highly enantioselective cascade synthesis of spiropyrazolones. Org. Biomol. Chem..

[12] Zhu C-L (2011). Enantioselective base-free electrophilic amination of benzofuran-2(3h)-ones: Catalysis by binol-derived p-spiro quaternary phosphonium salts. Angew. Chem. Int. Ed..

[13] Bondock S, Rabie R, Etman HA, Fadda AA (2008). Synthesis and antimicrobial activity of some new heterocycles incorporating antipyrine moiety. Eur. J. Med. Chem..

[14] Wan J-P, Lin Y, Huang Q, Liu Y (2014). Diastereoselective construction of tetrahydropyridine fused bicyclic structures via three-component domino reaction. J. Org. Chem..

[15] Chen X-B, Liu Z-C, Yang L-F, Yan S-J, Lin J (2014). A Three-component catalyst-free approach to regioselective synthesis of dual highly functionalized fused pyrrole derivatives in water–ethanol media: Thermodynamics versus kinetics. ACS Sustain. Chem. Eng..

[16] Song Z, Huang X, Yi W, Zhang W (2016). One-pot reactions for modular synthesis of polysubstituted and fused pyridines. Org. Lett..

[17] Mancebo-Aracil J, Nájera C, Sansano JM (2013). Multicomponent synthesis of unnatural pyrrolizidines using 1,3-dipolar cycloaddition of proline esters. Chem. Commun..

[18] Zhang X, Legris M, Muthengi A, Zhang W (2017). [3+2] Cycloaddition-based one-pot synthesis of 3,9-diazabicyclo[4.2.1]nonane-containing scaffold. Chem. Heterocycl. Compd..

[19] Selva V (2017). Diastereoselective [3 + 2] vs [4 + 2] cycloadditions of nitroprolinates with α, β-unsaturated aldehydes and electrophilic alkenes: An example of total periselectivity. J. Org. Chem..

[20] Zhang X (2018). One-pot double [3 + 2] cycloadditions for diastereoselective synthesis of pyrrolidine-based polycyclic systems. J. Org. Chem..

[21] Yamazaki T, Shinohara N, Kitazume T, Sato S (1995). Highly diastereoselective sequential enolate-Michael addition-ireland Claisen rearrangement. J. Org. Chem..

[22] Srivastava N, Banik BK (2003). Bismuth nitrate-catalyzed versatile Michael reactions. J. Org. Chem..

[23] Krause N, Hoffmann-Röder A (2001). Recent advances in catalytic enantioselective Michael additions. Synthesis.

[24] Okino T, Hoashi Y, Takemoto Y (2003). Enantioselective Michael reaction of malonates to nitroolefins catalyzed by bifunctional organocatalysts. J. Am. Chem. Soc..

[25] Sivasankara C, Satham L, Namboothiri INN (2017). One-pot construction of functionalized spiro-dihydronaphthoquinone-oxindoles via Hauser–Kraus annulation of sulfonylphthalide with 3-alkylideneoxindoles. J. Org. Chem..

[26] Wang L-L (2010). Highly organocatalytic asymmetric Michael–ketone aldol–dehydration domino reaction: Straightforward approach to construct six-membered spirocyclic oxindoles. Chem. Commun..

[27] Ball-Jones NR, Badillo JJ, Franz AK (2012). Strategies for the enantioselective synthesis of spirooxindoles. Org. Biomol. Chem..

[28] Cheng D, Ishihara Y, Tan B, Barbas CF (2014). Organocatalytic asymmetric assembly reactions: Synthesis of spirooxindoles via organocascade strategies. ACS Catal..

[29] Li Z, Li J, Yang J (2017). Chemoselective double michael addition: Synthesis of 2,6-diarylspiro[cyclohexane-1,3′-indoline]-2′,4-diones via addition of indolin-2-one to divinyl ketones. J. Chem. Res..

[30] Wu B (2012). Highly enantioselective synthesis of spiro[cyclohexanone-oxindoles] and spiro[cyclohexanone-pyrazolones] by asymmetric cascade [5+1] double Michael reactions. Eur. J. Org. Chem..

[31] Géant P-Y, Urban M, Remeš M, Císařová I, Veselý J (2013). Enantioselective organocatalytic synthesis of sulfur-containing spirocyclic compounds. Eur. J. Org. Chem..

[32] Scala A (2014). Direct synthesis of C3-mono-functionalized oxindoles from N-unprotected 2-oxindole and their antileishmanial activity. Biorg. Med. Chem..

[33] Saha S (2016). A novel spirooxindole derivative inhibits the growth of Leishmania donovani parasites both in vitro and in vivo by targeting type ib topoisomerase. Antimicrob. Agents Chemother..

[34] Pulvertaft RJV, Hoyle GF (1960). Stages in the life-cycle of *Leishmania donovani*. Trans. R. Soc. Trop. Med. Hyg..

[35] French JB (2011). The *Leishmania donovani* UMP synthase is essential for promastigote viability and has an unusual tetrameric structure that exhibits substrate-controlled oligomerization. J. Biol. Chem..

[36] Moghaddam FM, Khodabakhshi MR, Kiamehr M, Ghahremannejad Z (2013). Synthesis of novel pentacyclic thiopyrano indole-annulated benzo-δ-sultone derivatives via a domino Knoevenagel-hetero-Diels–Alder reaction in water. Tetrahedron Lett..

[37] Moghaddam FM, Saeidian H, Mirjafary Z, Taheri S, Kheirjou S (2009). A new and facile synthesis of thieno[2,3-b]indole derivatives via condensation of isocyanide and indolin-2-thiones. Synlett.

[38] Pettersen EF (2004). UCSF Chimera—A visualization system for exploratory research and analysis. J. Comput. Chem..

[39] Goodsell DS, Olson AJ (1990). Automated docking of substrates to proteins by simulated annealing. Proteins: Struct. Funct. Genet..

[40] Keith TA, Millam JM (2016). GaussView, Version 6.1, Roy Dennington.

[41] Cossi M, Barone V (2001). Time-dependent density functional theory for molecules in liquid solutions. J. Chem. Phys..

[42] Zhao Y, Schultz NE, Truhlar DG (2005). Exchange-correlation functional with broad accuracy for metallic and nonmetallic compounds, kinetics, and noncovalent interactions. J. Chem. Phys..

[43] Hariharan PC, Pople JA (1973). The influence of polarization functions on molecular orbital hydrogenation energies. Theor. Chim. Acta.

[44] Hehre W, Yu J, Klunzinger P, Lou L (2000). Spartan Software. Wavefunction.

[45] Frisch MJ (2010). Gaussian 16, Revision A.03.

[46] Sheldrick GM (2015). SHELXT—Integrated space-group and crystalstructure determination. Acta Cryst..

[47] Sheldrick GM (2015). Crystal structure refinement with SHELXL. Acta Cryst..

[48] Spek AL (2009). Structure validation in chemical crystallography. Acta Cryst..

[49] Macrae CF (2008). Mercury CSD 2.0—New features for the visualization and investigation of crystal structures. Appl. Cryst..

[50] Kondhare D, Deshmukh S, Lade H (2019). Curcumin analogues with aldose reductase inhibitory activity: Synthesis, biological evaluation, and molecular docking. Processes.

[51] Mane V, Baiju TV, Namboothiri NN (2018). Synthesis of functionalized thieno[2,3-b]indoles via one-pot reaction of indoline-2-thiones with Morita–Baylis–Hillman and Rauhut–Currier adducts of nitroalkenes. ACS Omega.

[52] Moghaddam FM (2010). Facile entry to polycyclic indolylhydroquinoline skeletons via tandem C-alkylation and intramolecular S-alkylation. Tetrahedron.

