# Supplementary material for: Highly diastereoselective cascade [5 + 1] double Michael reaction, a route for the synthesis of spiro(thio)oxindoles
Source: Sci Rep. 2021 Nov 24;11:22834. doi: 10.1038/s41598-021-01766-6 (PMC8613191; doi:10.1038/s41598-021-01766-6)
Supplement: Supplementary file 1 — Supplementary Information 1. [file 41598_2021_1766_MOESM1_ESM.pdf]

Supporting Information

Highly Diastereoselective Cascade [5+1] Double Michael Reaction, a Route for the Synthesis of Spiro(thio)oxindoles

Firouz Matloubi Moghaddam[1]*, Vahid Saberi[1], Ashkan Karimi[1,2]

[1] Laboratory of Organic Synthesis and Natural Products, Department of Chemistry, Sharif University of Technology, 111559516 Tehran, Iran

[2] Department of Chemistry, McGill University, H3A-0B8 Montreal, Canada

Table of contents

1. Figures and tables S2

2. NMR spectra S4

3. Crystallography data S24

5. Cartesian coordinates and energies........ S36

Figures and tables:

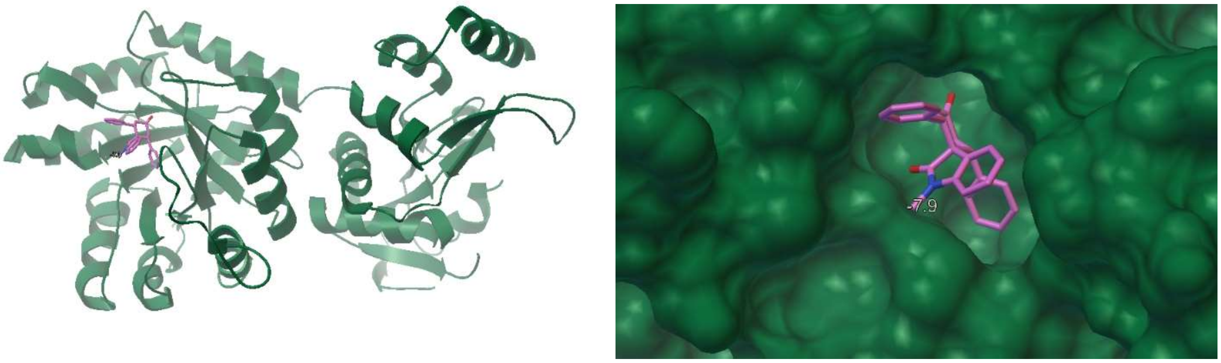

Figure S1. Interaction between *cis*-spiro oxindole **2** with OPRT domain of the LdUMPS (PDB ID: 2WNS). The figure is drawn using UCSF chimera 1.8.

Table S1. Electronic energy (Hartree) of *cis/trans* spiro oxindole and thiooxindole **1-4** calculated using B3LYP/6-31+G(d) in gas phase.

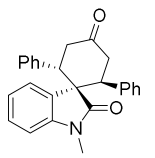	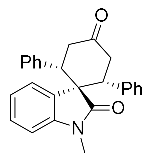	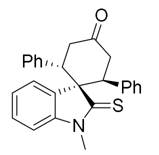	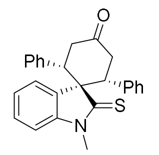
**1**	**2**	**3**	**4**
-1203.218 Hartree	-1203.209 Hartree	-1524.616 Hartree	-1524.624 Hartree

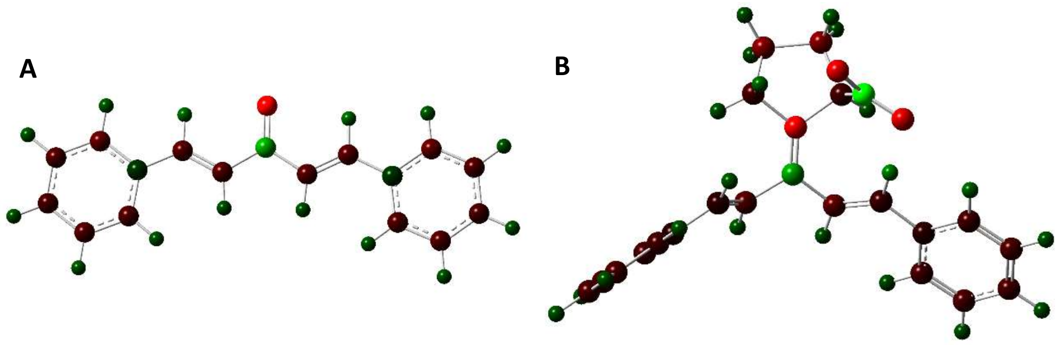

Figure S2. Mulliken charge distribution on (A) dibenzal ketone and (B) dibenzal iminium the product of reaction of L-proline with dibenzal ketone. Calculations were performed using B3LYP/6-31G(d) in gas phase.

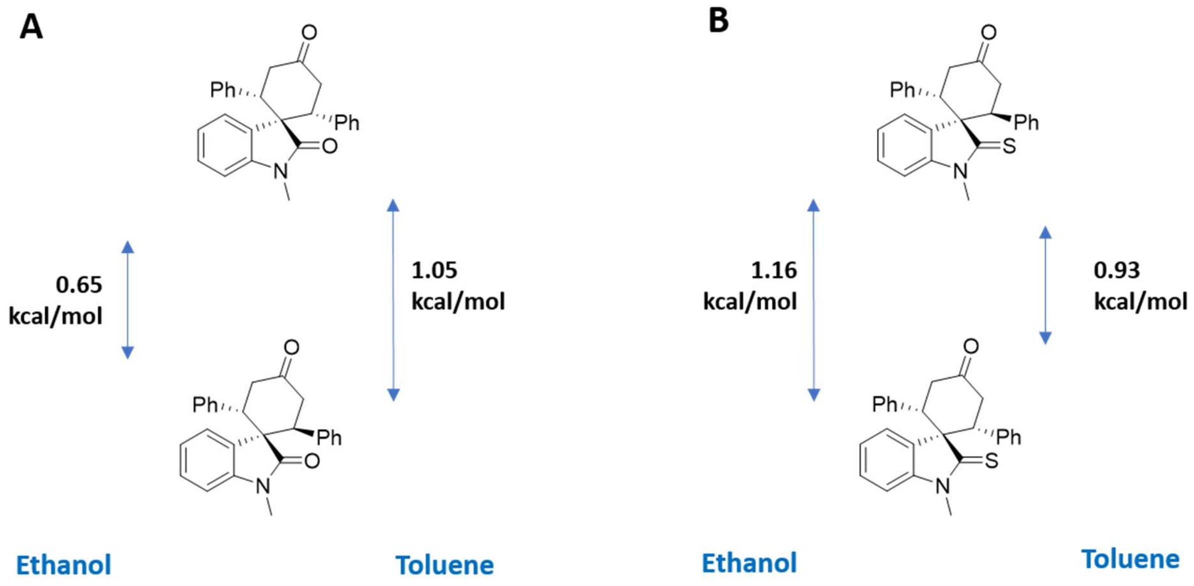

Figure S3. The energy diagram of *cis* and *trans* (A) oxindole (B) thiooxindole in ethanol and toluene.

NMR spectra:

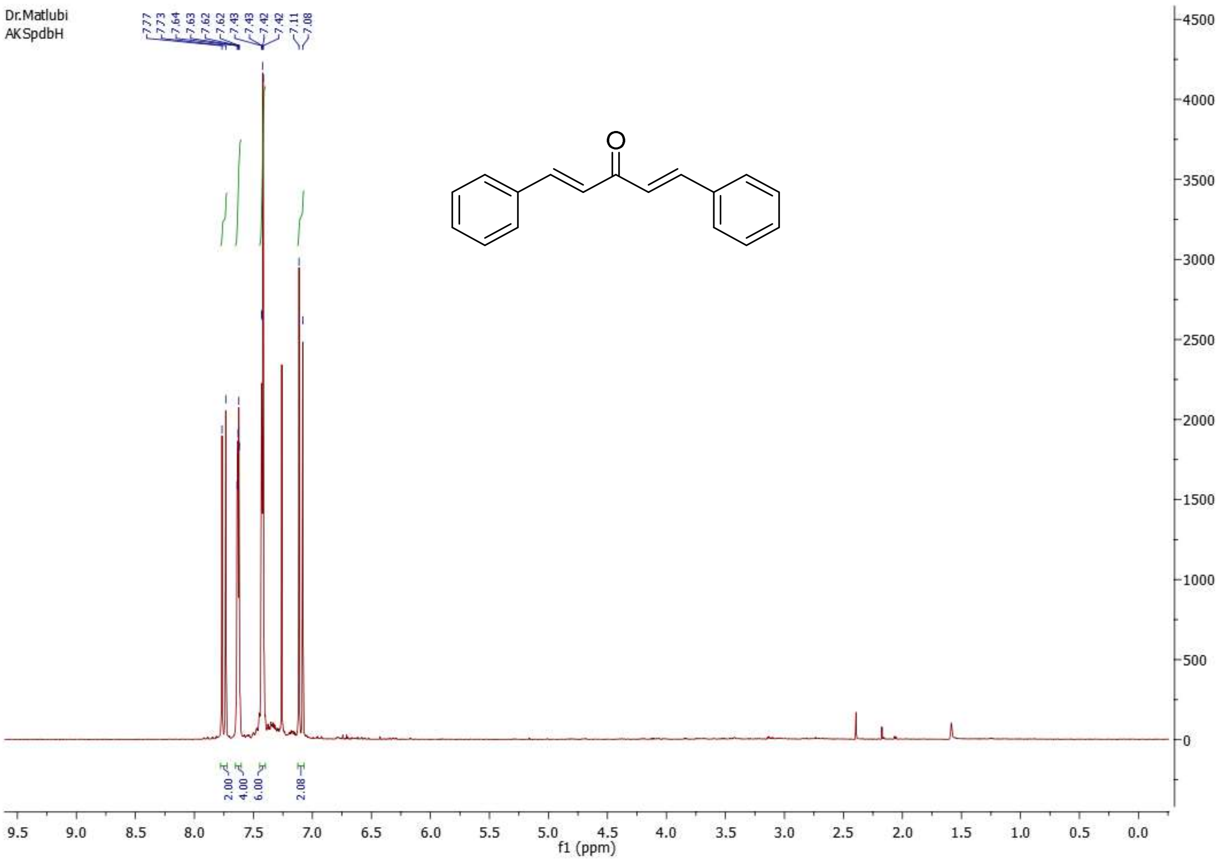

^{1}H NMR spectrum ($CDCl_3$, 500 MHz)

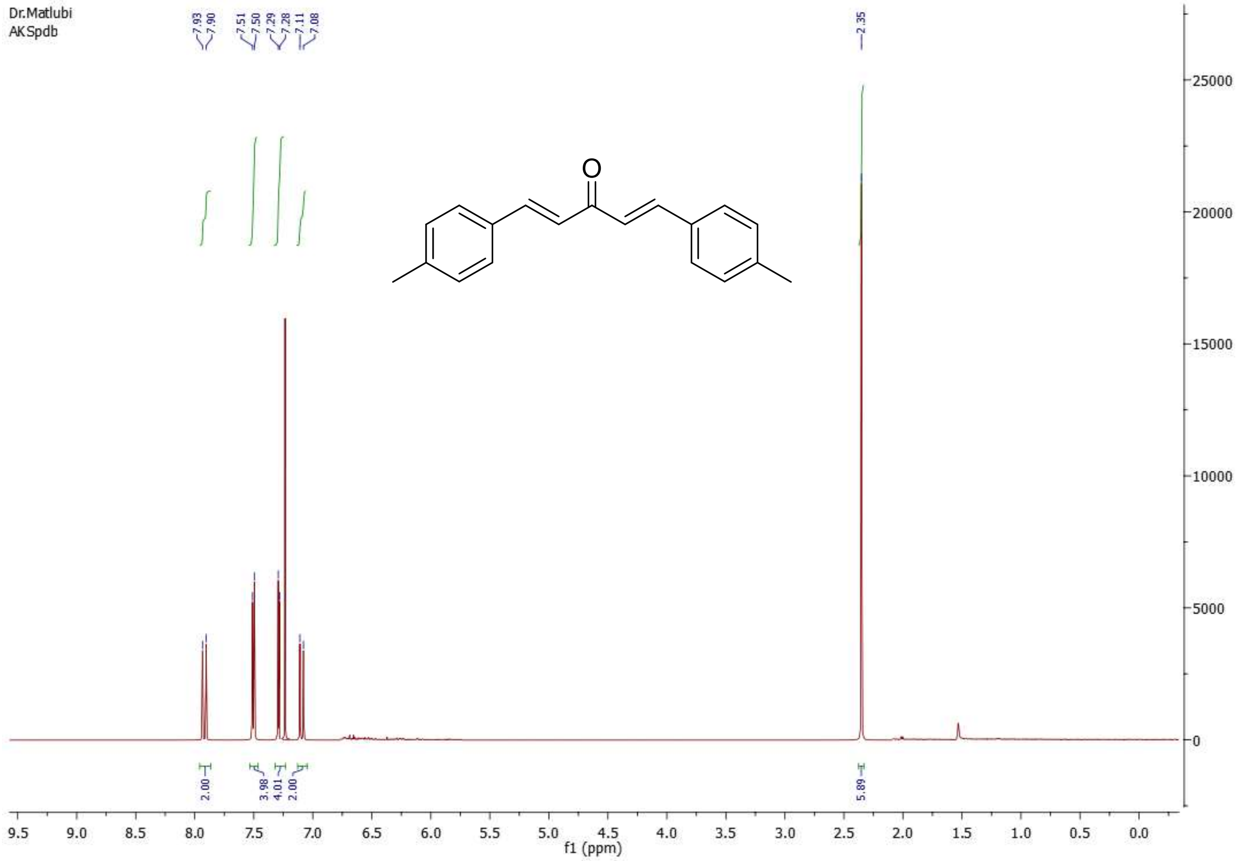

^{1}H NMR spectrum ($CDCl_3$, 500 MHz)

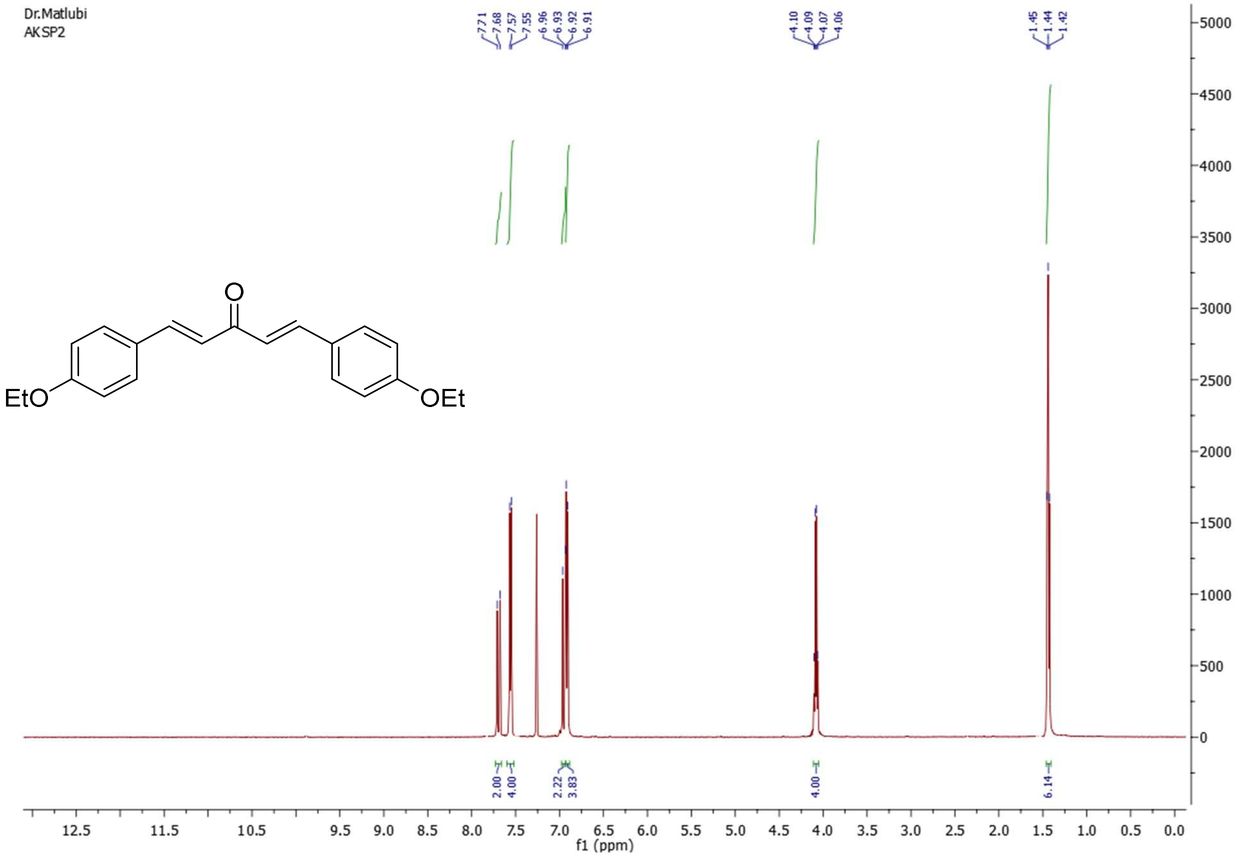

^1H NMR spectrum ($CDCl_3$, 500 MHz)

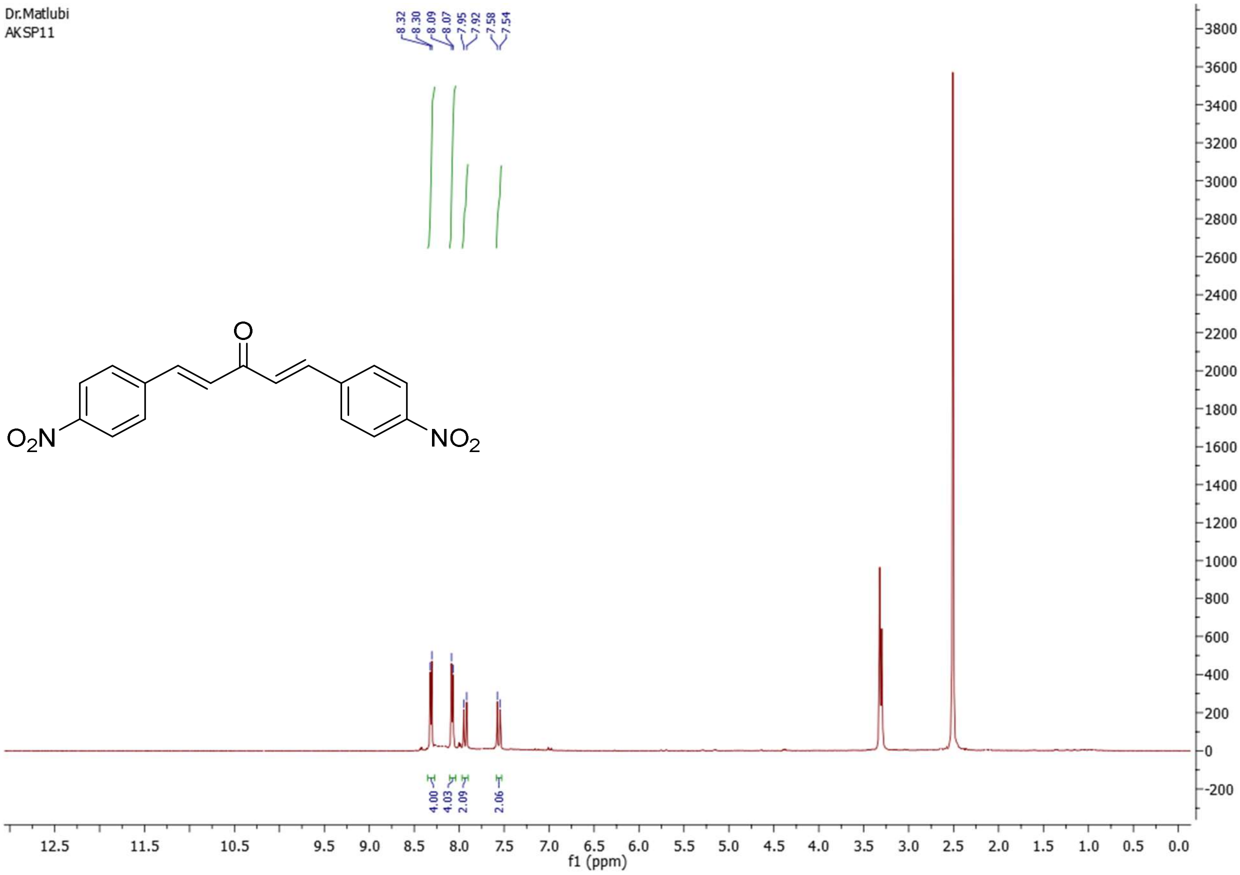

^{1}H NMR spectrum (d6-DMSO, 500 MHz)

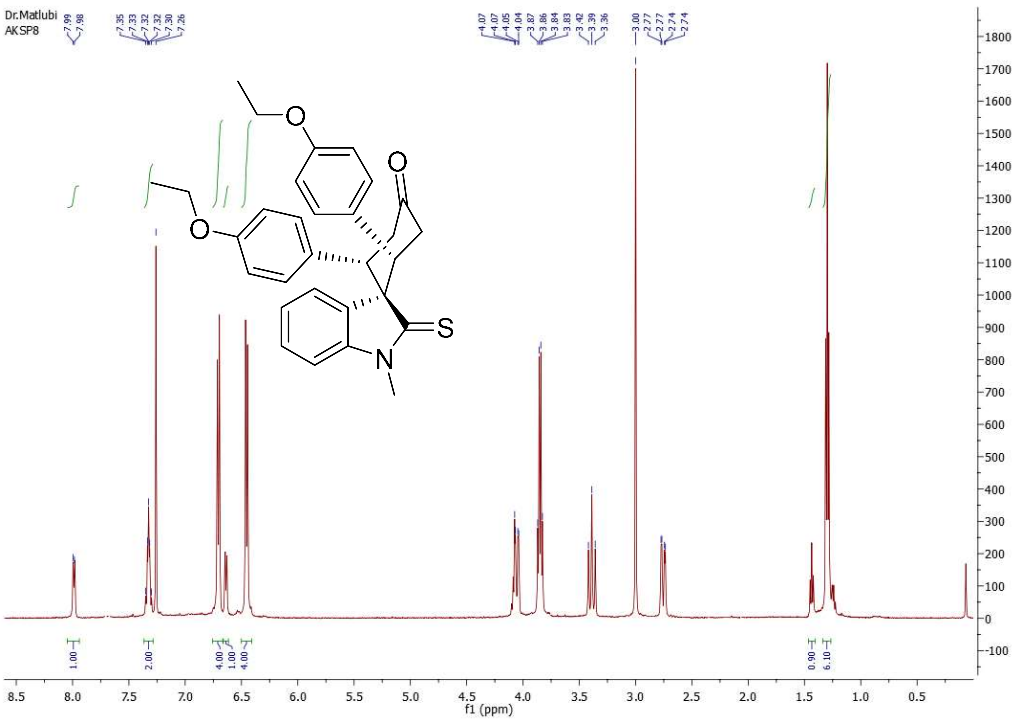

^{1}H NMR spectrum ($CDCl_3$, 500 MHz)

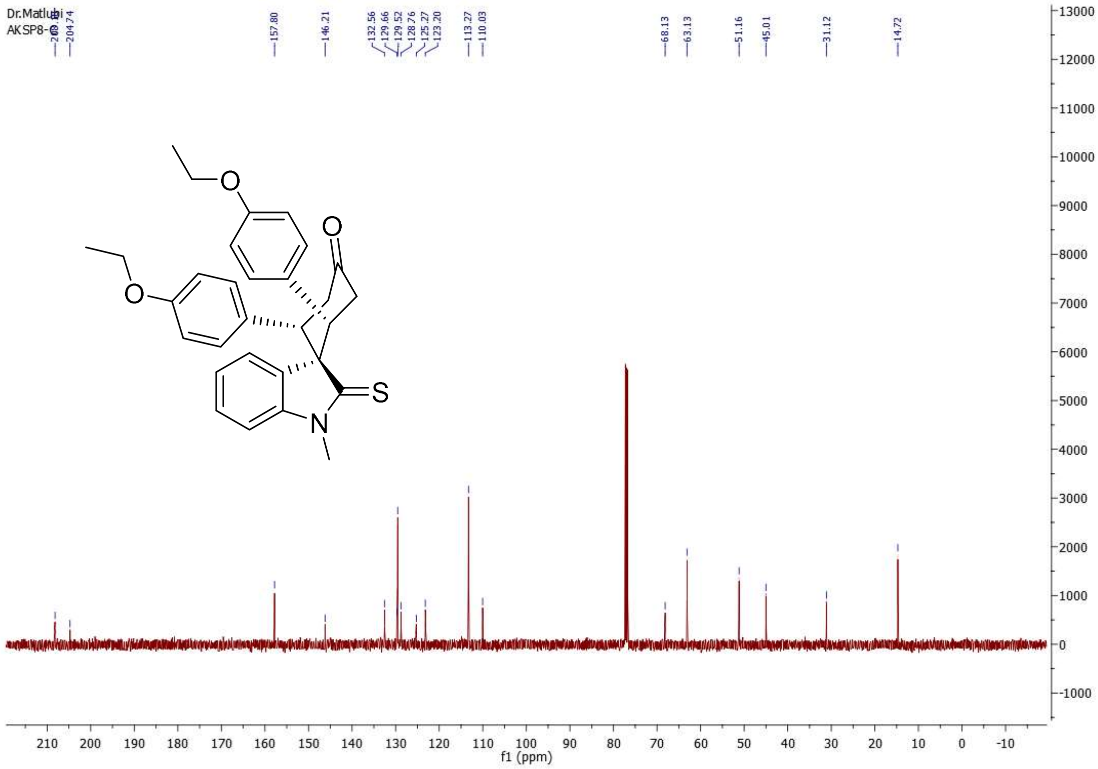

^{13}C NMR spectrum ($CDCl_3$, 125.8 MHz)

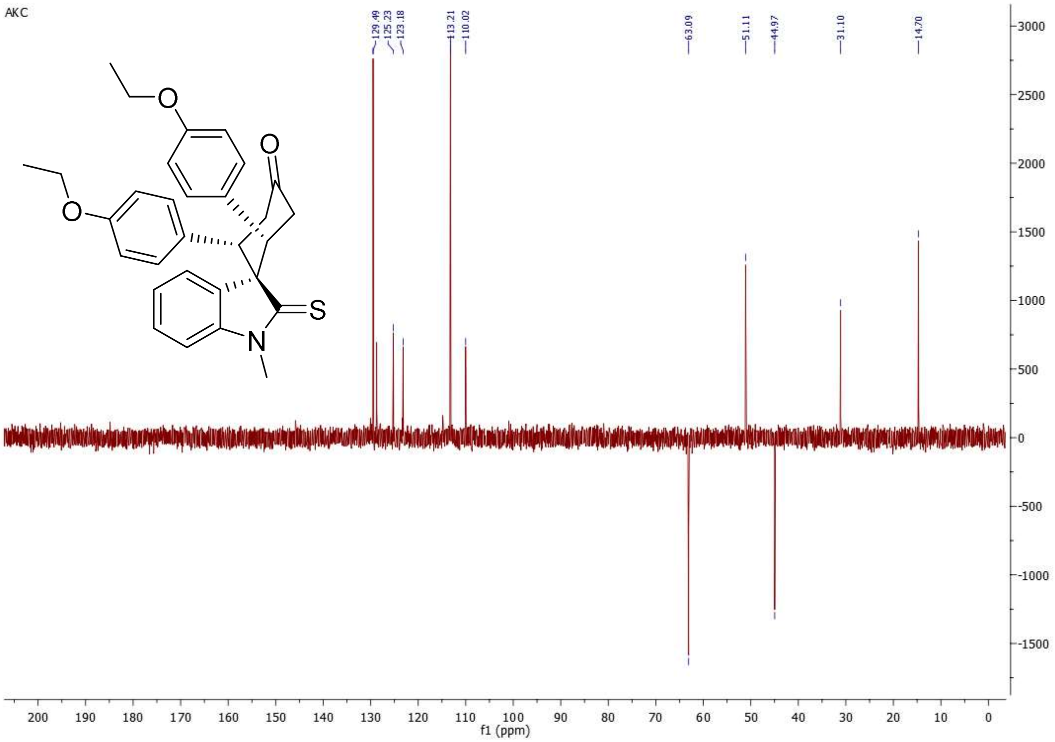

DEPT 135

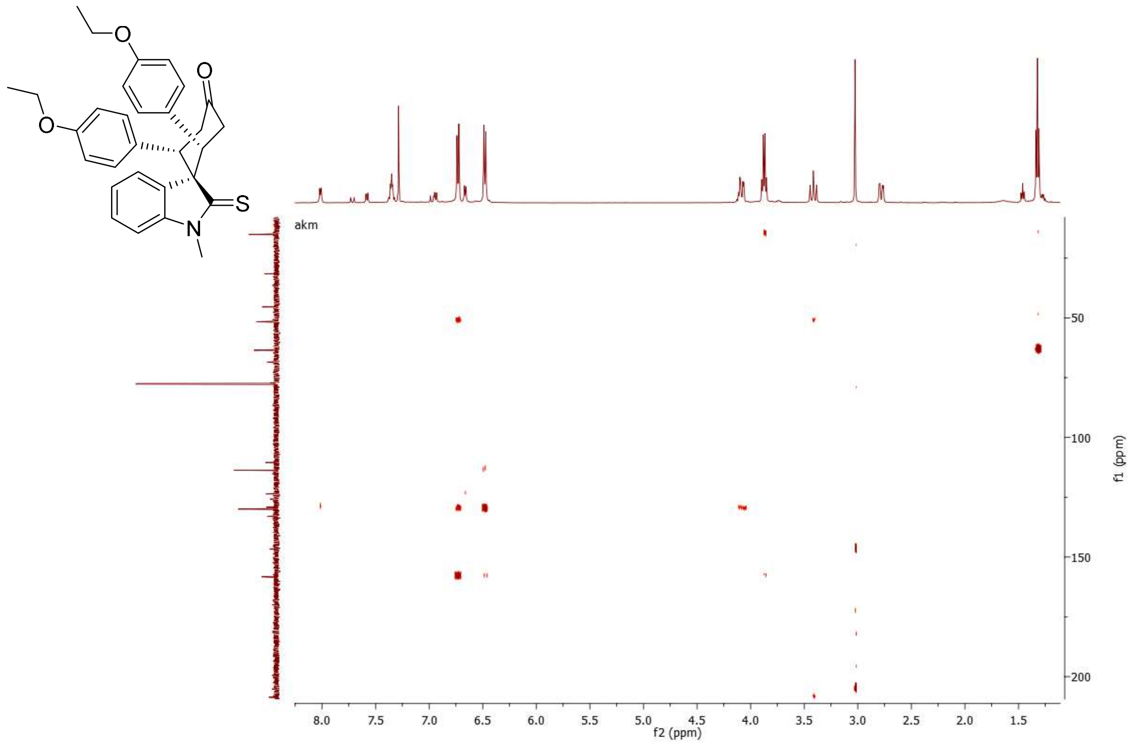

HMBC

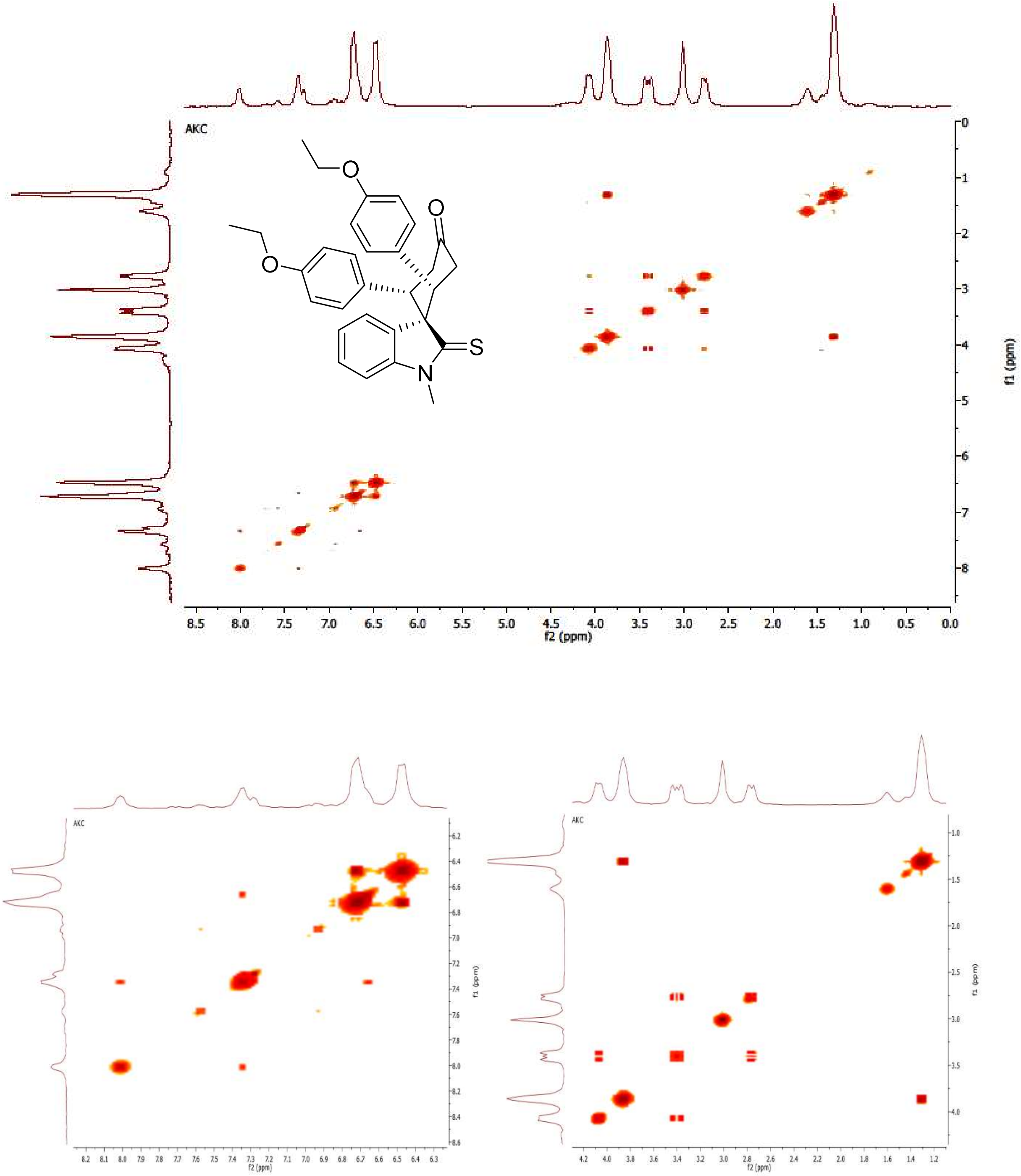

COSY

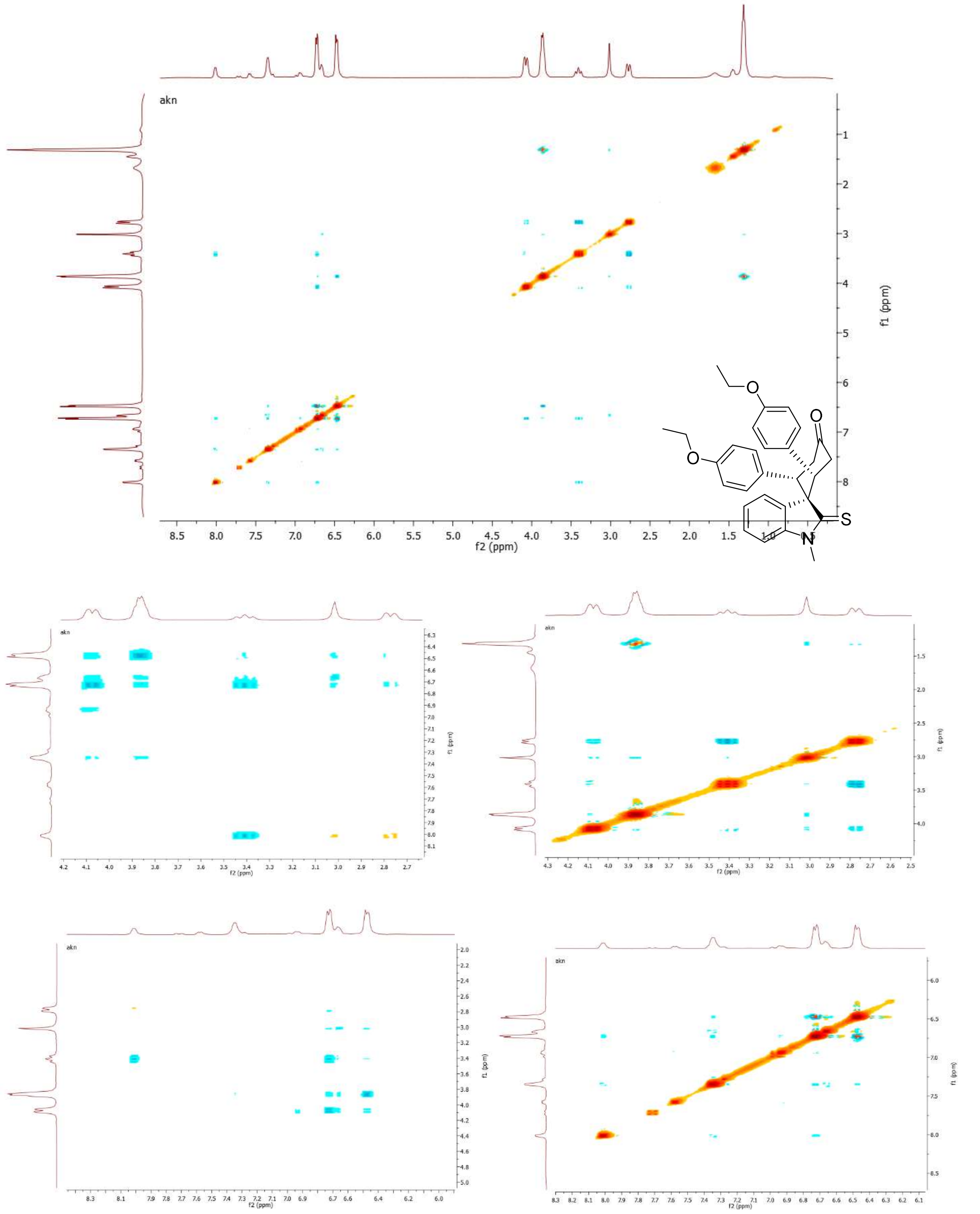

NOESY

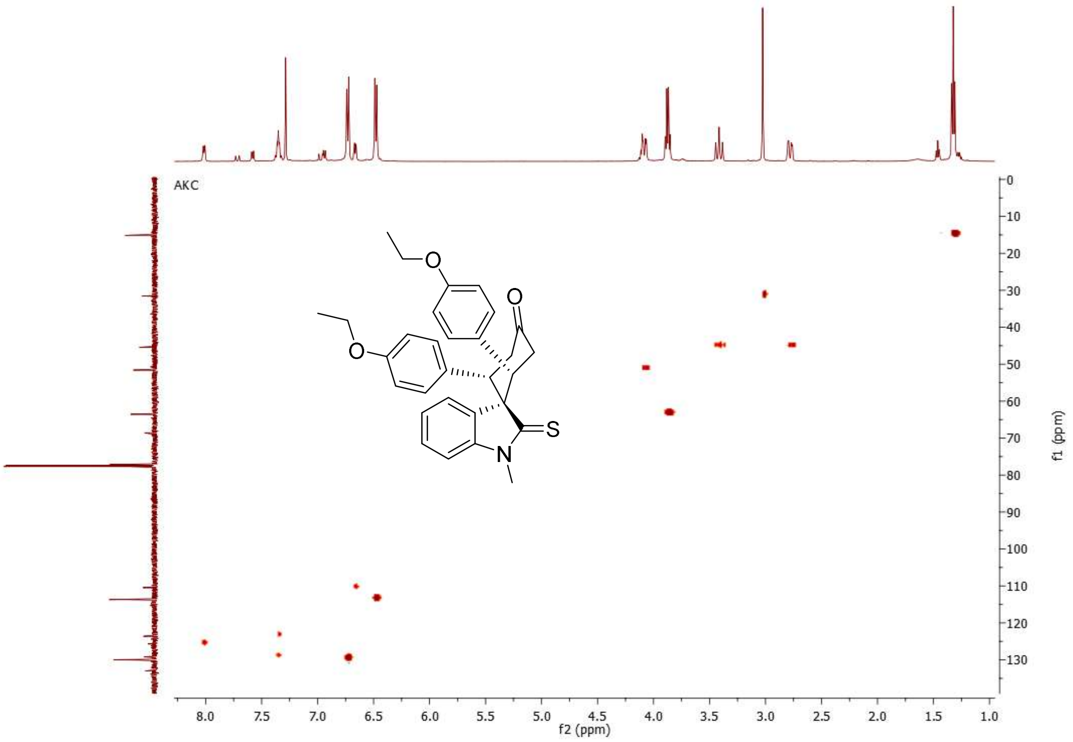

HSQC

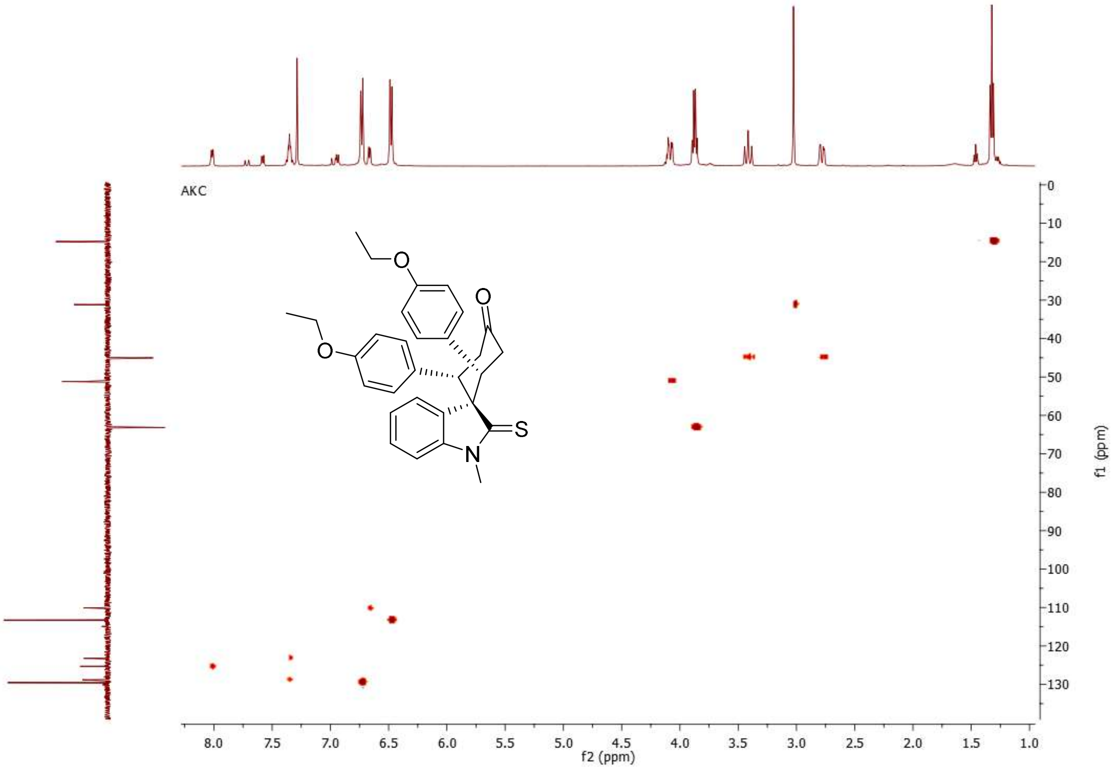

HSQC (on DEPT 135)

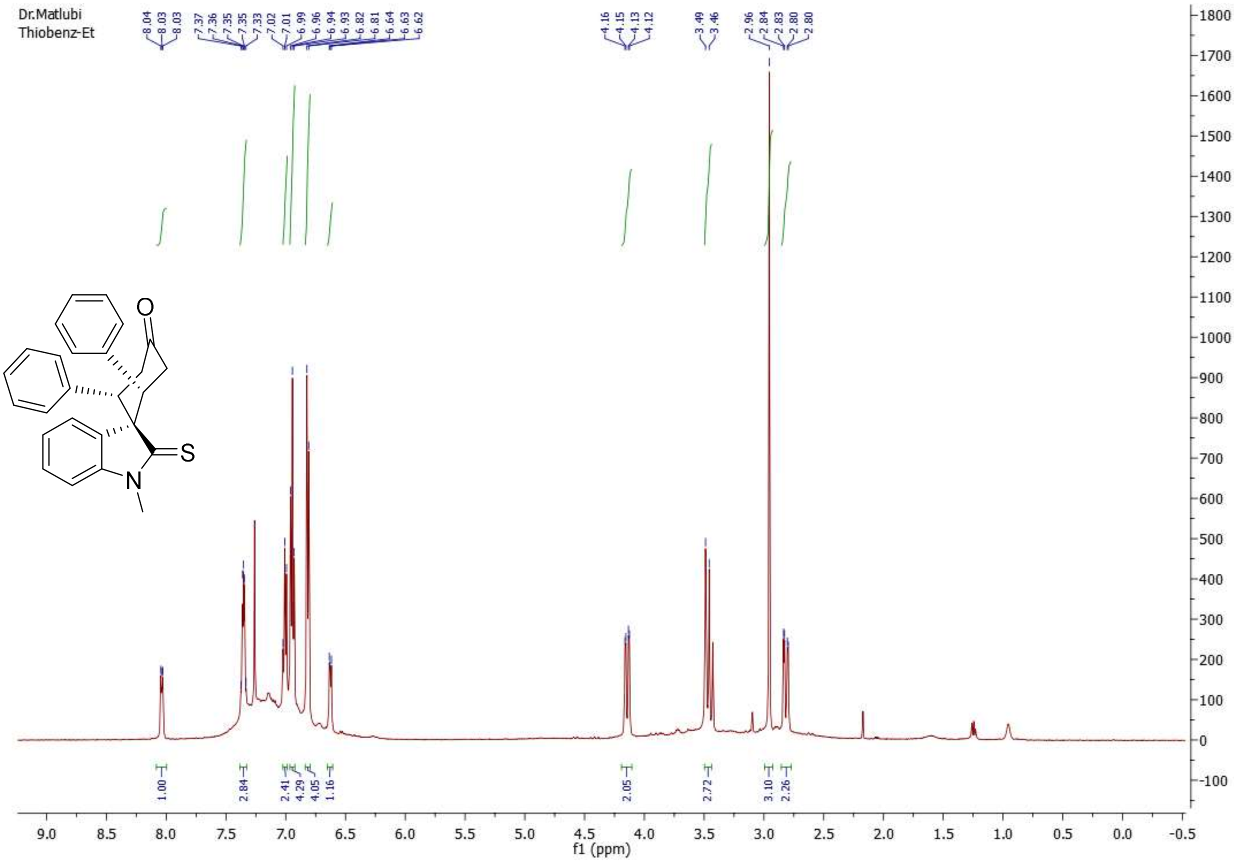

^{1}H NMR spectrum ($CDCl_3$, 500 MHz)

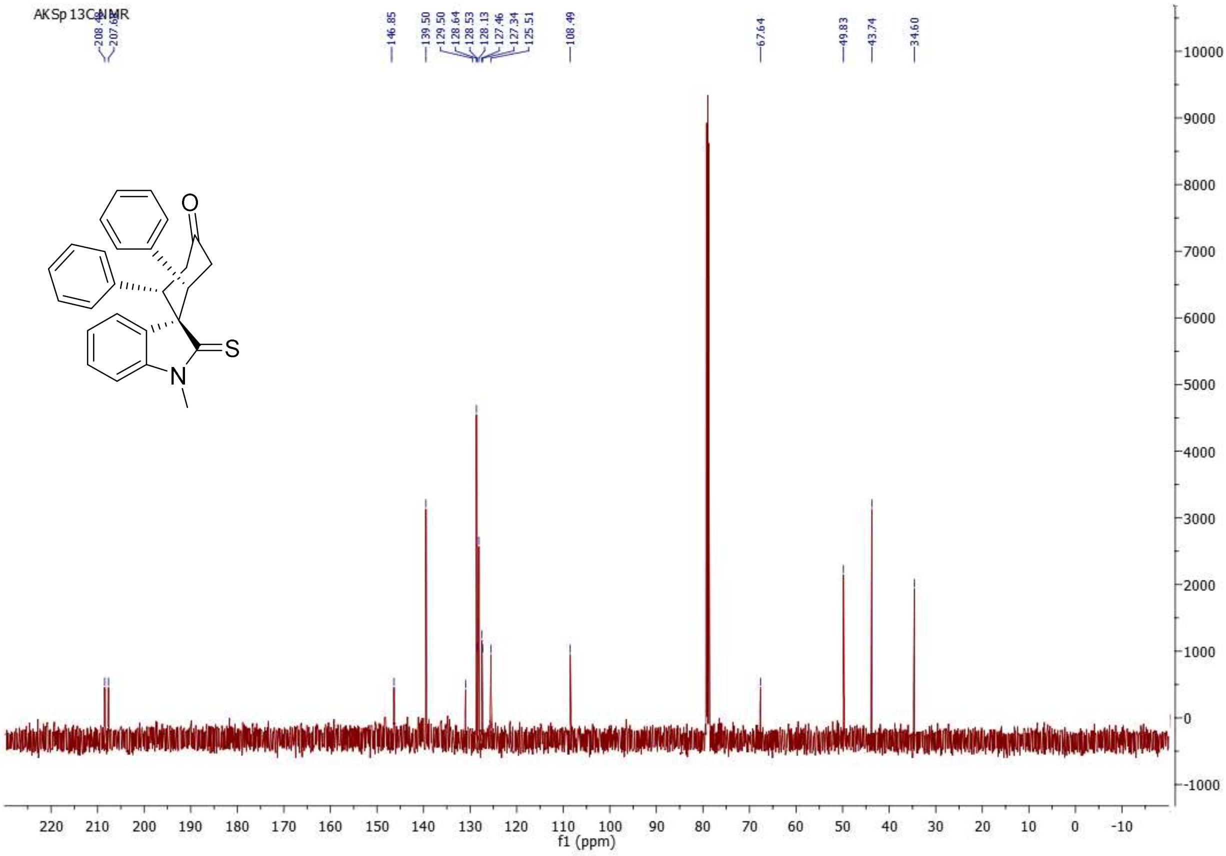

^{13}C NMR spectrum ($CDCl_3$, 125.8 MHz)

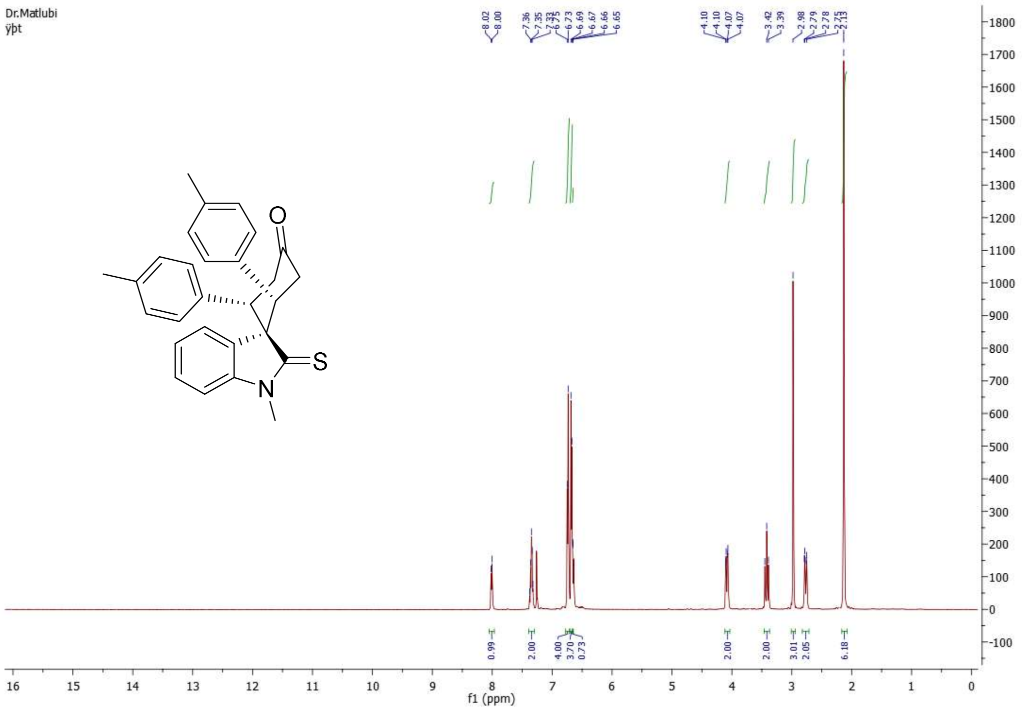

^{1}H NMR spectrum ($CDCl_3$, 500 MHz)

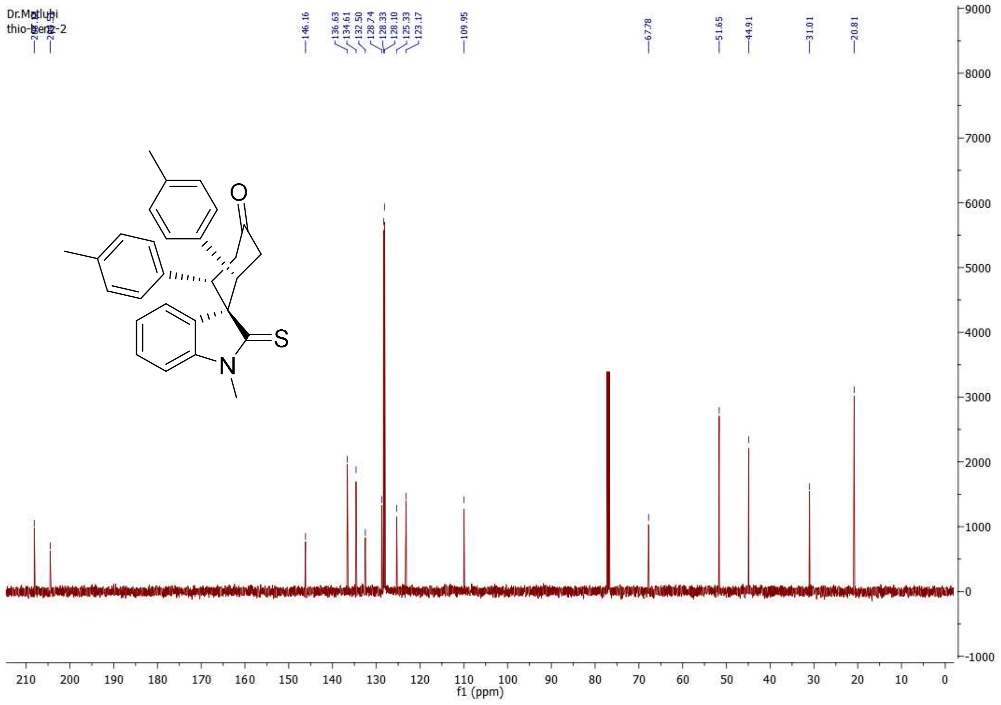

^{13}C NMR spectrum ($CDCl_3$, 125.8 MHz)

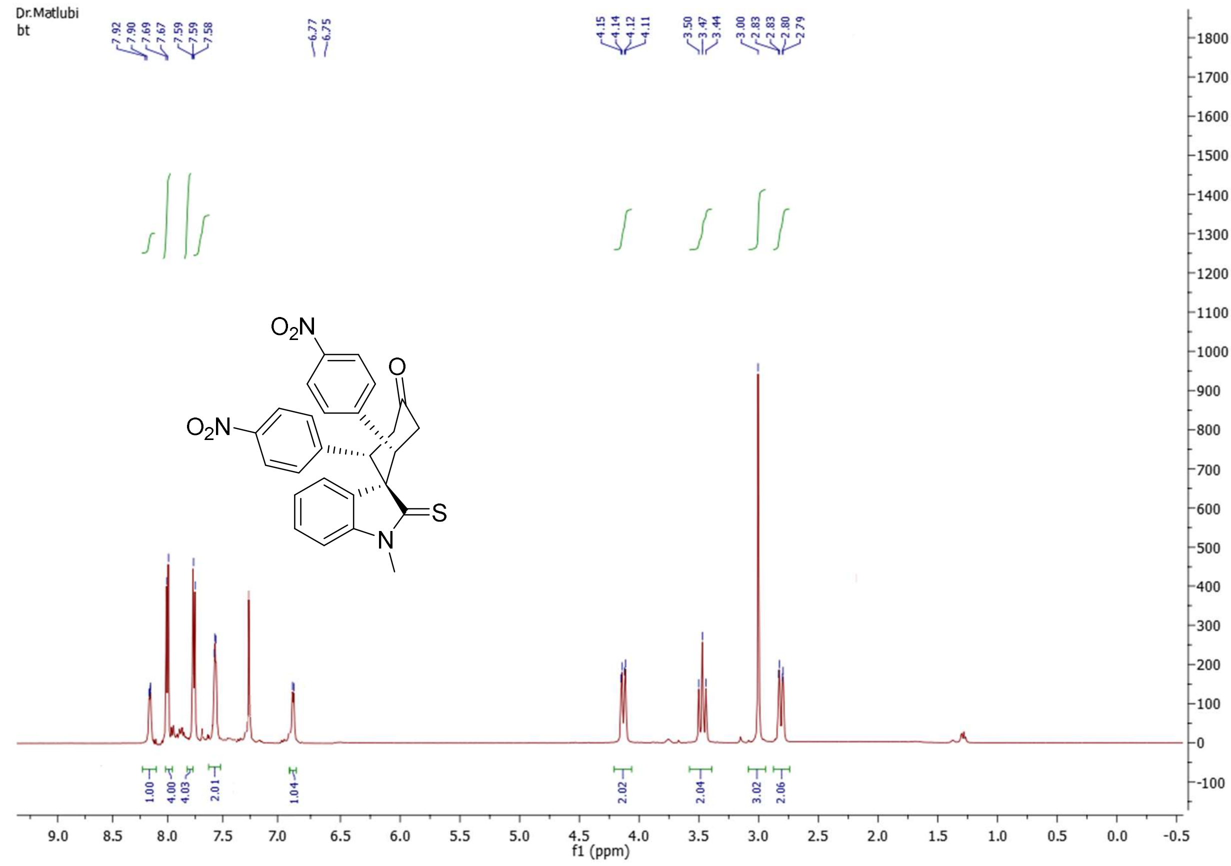

^{1}H NMR spectrum ($CDCl_3$, 500 MHz)

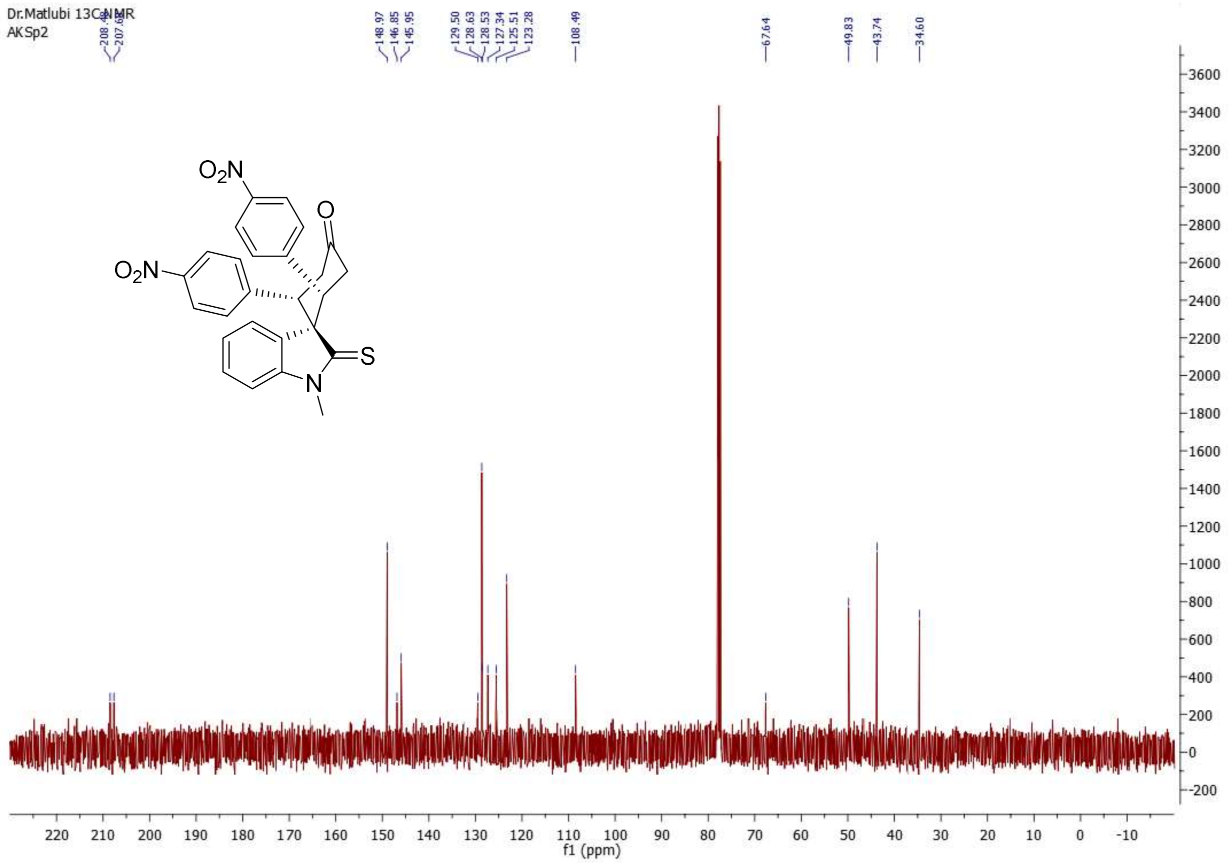

^{13}C NMR spectrum ($CDCl_3$, 125.8 MHz)

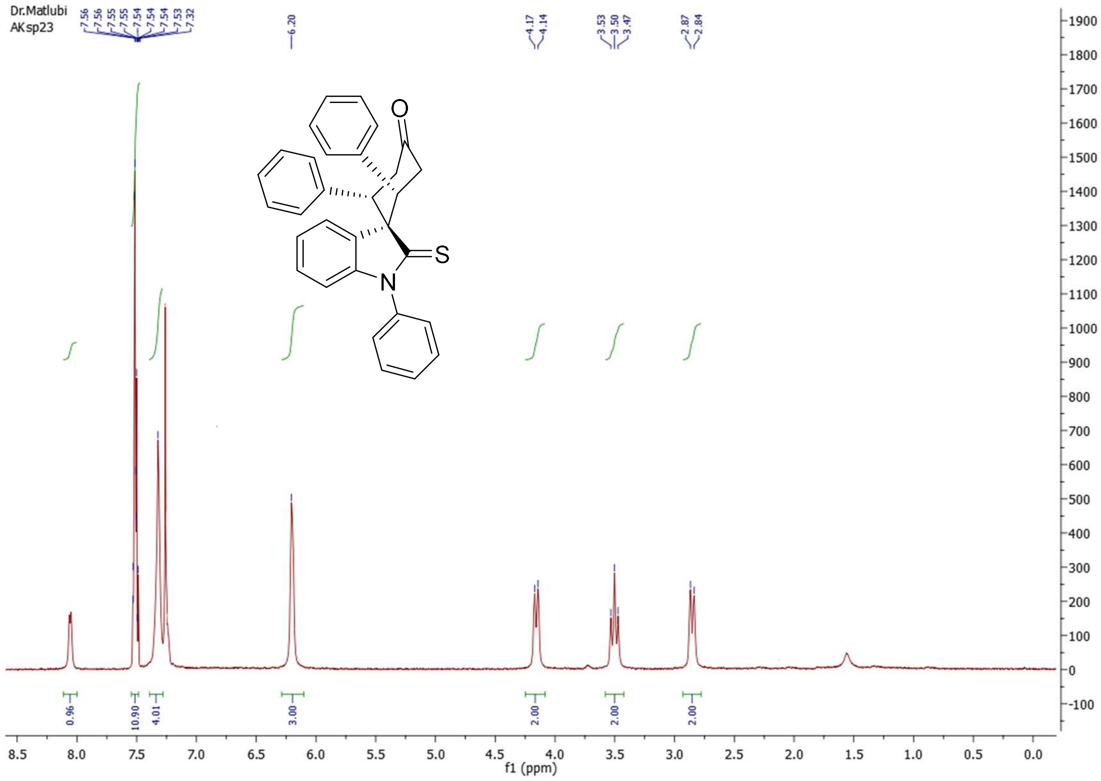

^{1}H NMR spectrum ($CDCl_3$, 500 MHz)

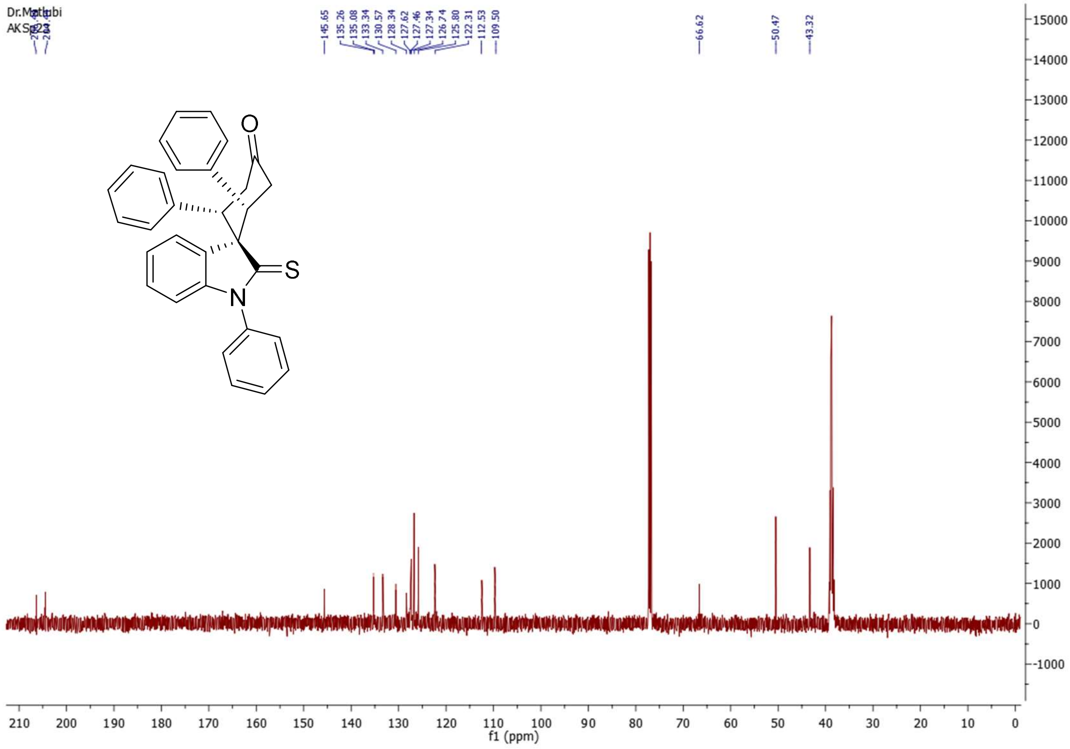

^{13}C NMR spectrum ($CDCl_3$ and d6-DMSO, 125.8 MHz)

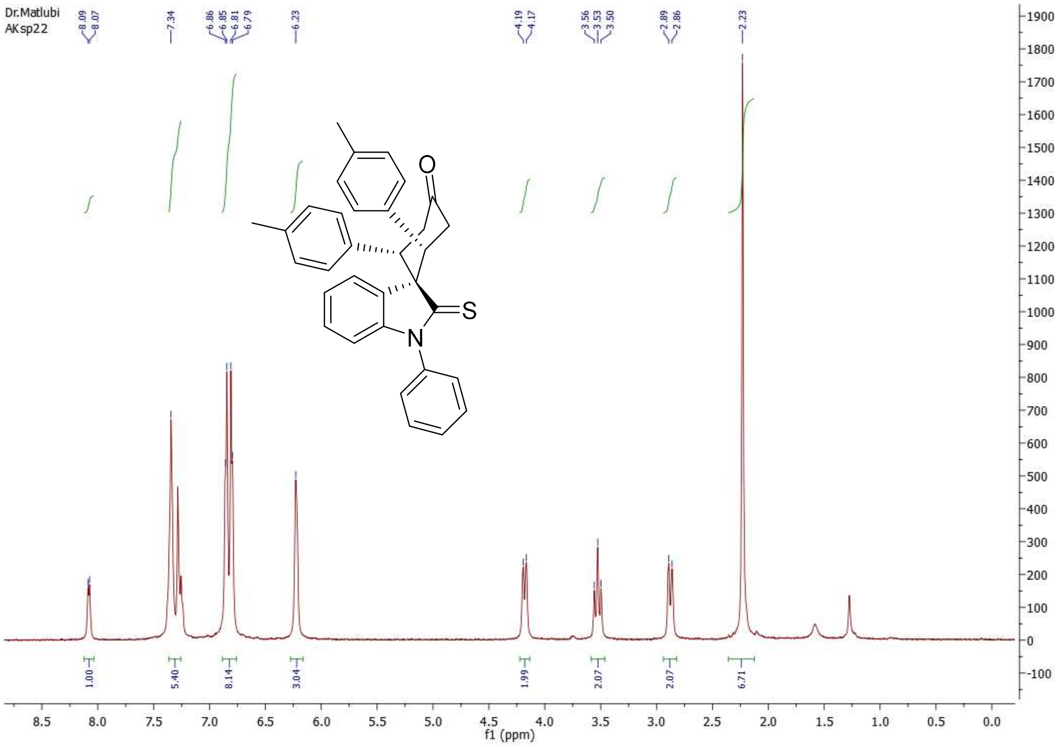

^{1}H NMR spectrum ($CDCl_3$, 500 MHz)

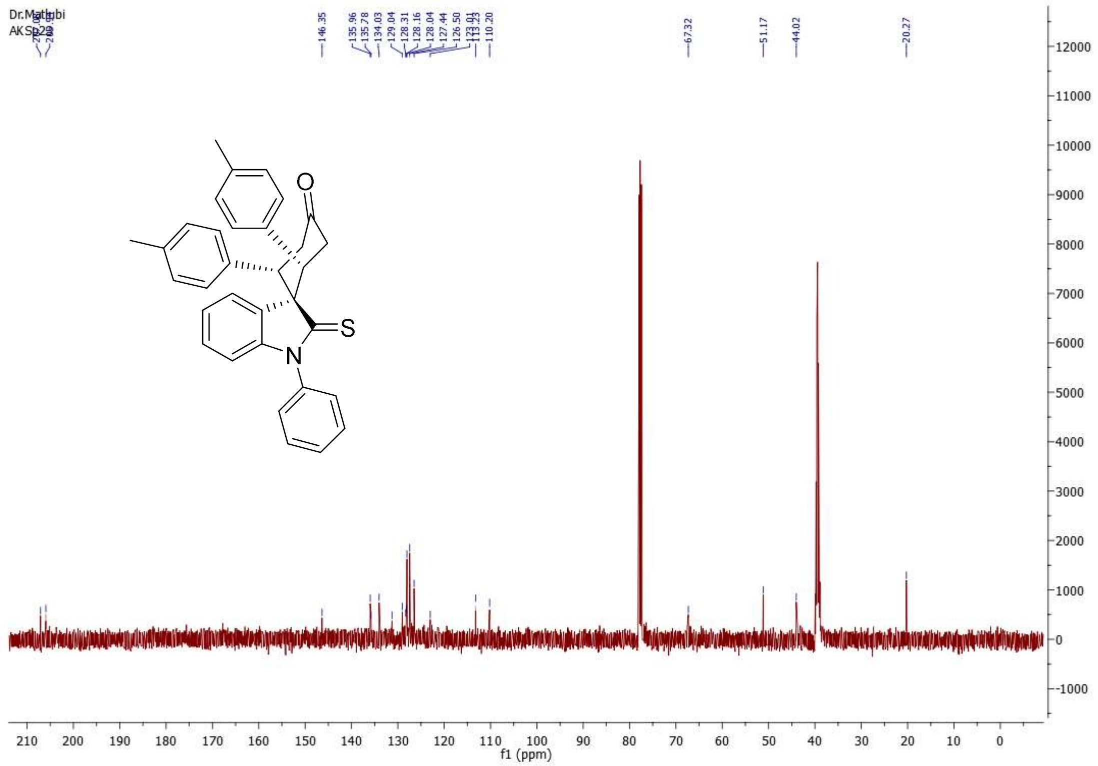

^{13}C NMR spectrum ($CDCl_3$ and d6-DMSO, 125.8 MHz)

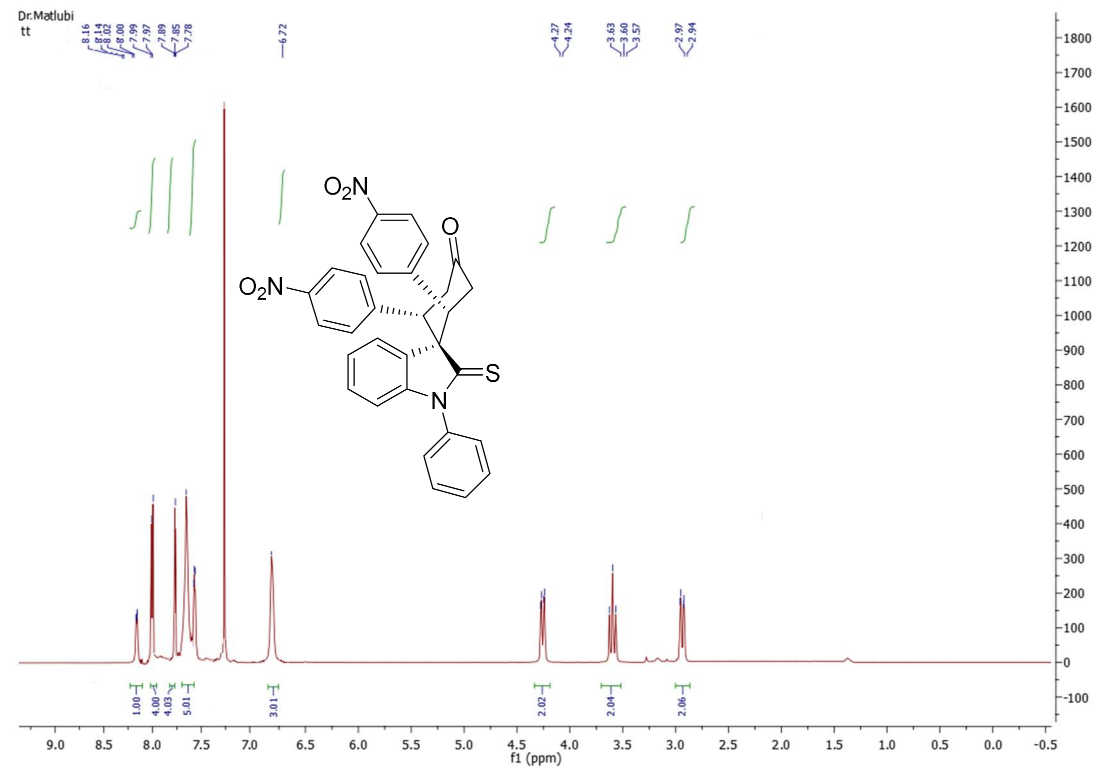

^{1}H NMR spectrum ($CDCl_3$, 500 MHz)

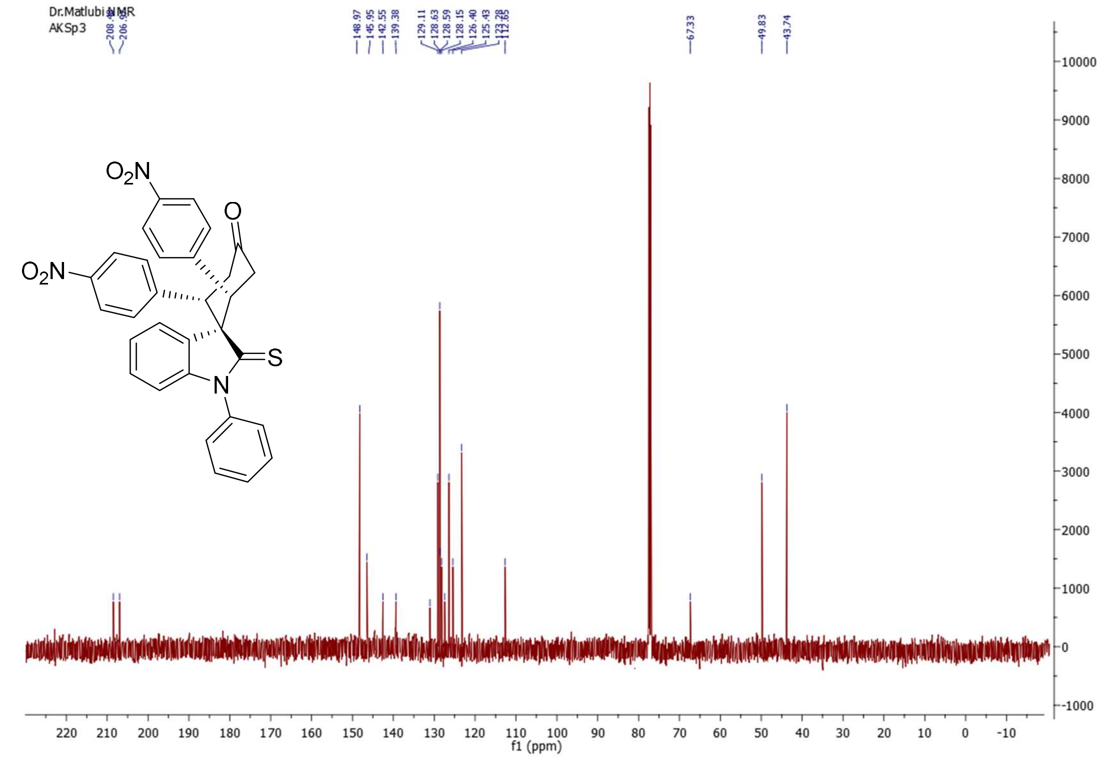

^{13}C NMR spectrum ($CDCl_3$, 125.8 MHz)

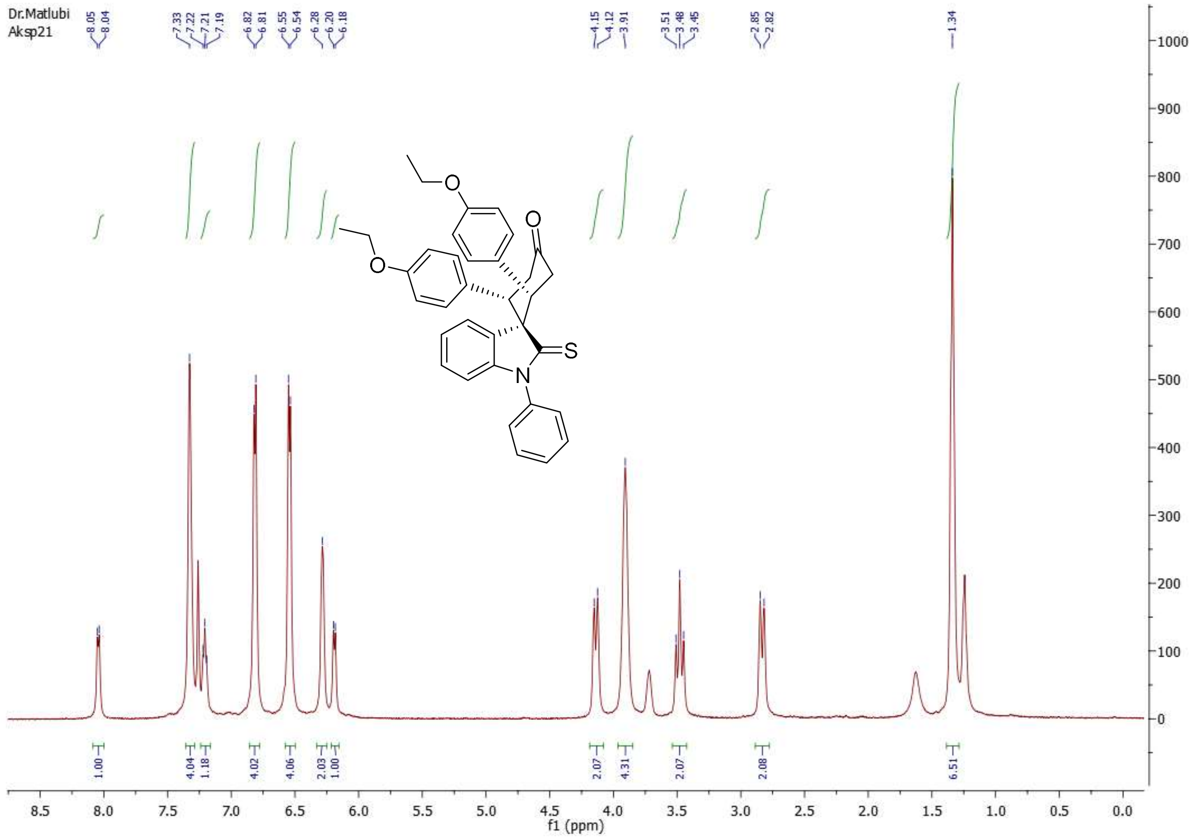

^{1}H NMR spectrum ($CDCl_3$, 500 MHz)

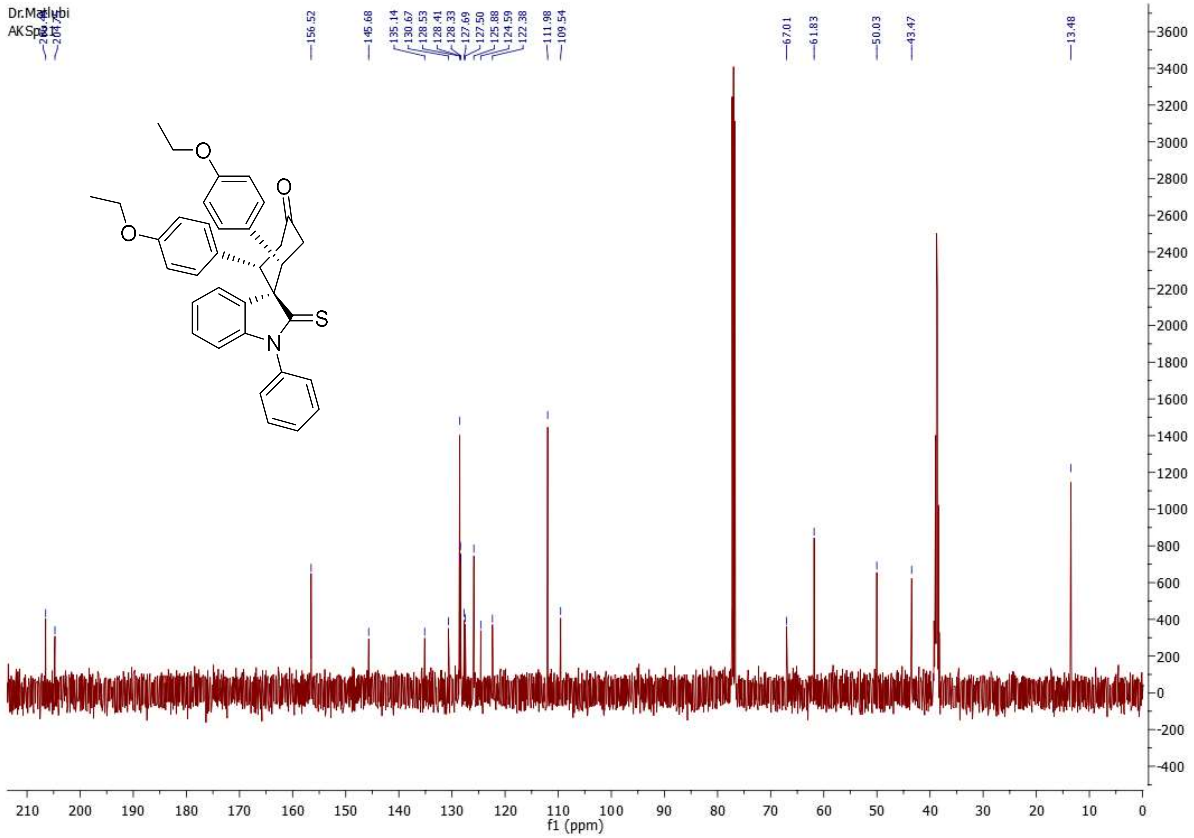

^{13}C NMR spectrum ($CDCl_3$, 125.8 MHz)

Crystallography data:

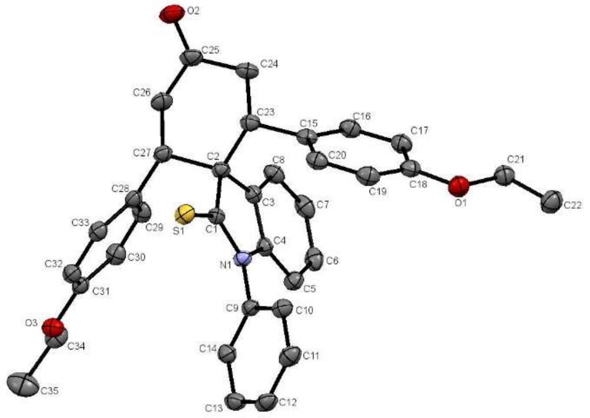

Empirical Formula	$C_{35} H_{33} N O_3 S$
Formula Weight	547.68
Crystal Color, Habit	pale yellow plate
Crystal Dimensions	0.240x0.160x0.080 mm
Crystal System	monoclinic
Lattice Type	P 2_1/c

a(Å)	16.7611(5)
b(Å)	10.7285(4)
c(Å)	16.8814(4)
α(°)	90
β(°)	113.469(1)
γ(°)	90
V($Å^3$)	2784.52(15)
Z	4
d($g\text{-}cm^{-3}$)	1.306

F(000)	1160
$\mu(cm^{-1})$	0.154

Intensity measurements:

Diffractometer	Bruker APEX II CCD
Monochromator	graphite
Radiation	MoKα (λ = 0.71069 Å)
Maximum theta	27.532 °
HKL ranges	-21 21 ; -13 13 ; -21 21
No. of Reflexions measured	Total: 19308 Unique: 6331 (R_{int} = 0.0360)
Absorption corrections	multi-scan; 0.6822 min, 0.7456 max

Structure solution and refinement:

Structure Solution	SHELXT-2014
Refinement	SHELXL-2014/7
Refinement type	Fsqd
Hydrogen atoms	constr
Parameters refined	363
Reflections/parameter	14
wR2	0.1042
**R1**	**3.74 %**
**Completeness**	**98.9 %**
Weights a, b	0.0520; 0.6732
GoF	1.038
Difference peak / hole (e $Å^{-3}$)	0.293(0.042) / -0.217(0.042)

Table S2. Atomic Coordinates (A x 10^4) and equivalent isotropic displacement parameters (A^2 x 10^3)

atom	x	y	z	U(eq)
S(1)	1741(1)	4434(1)	2205(1)	25(1)
O(1)	1119(1)	246(1)	5029(1)	30(1)
O(2)	-102(1)	8054(1)	3359(1)	36(1)
O(3)	5237(1)	8411(1)	3617(1)	27(1)
N(1)	2955(1)	4123(1)	3805(1)	20(1)
C(1)	2218(1)	4679(1)	3254(1)	20(1)
C(2)	1906(1)	5585(1)	3778(1)	21(1)
C(3)	2589(1)	5415(1)	4686(1)	21(1)
C(4)	3198(1)	4552(1)	4664(1)	20(1)
C(5)	3911(1)	4189(1)	5388(1)	26(1)
C(6)	3987(1)	4695(1)	6176(1)	29(1)
C(7)	3381(1)	5534(1)	6220(1)	30(1)
C(8)	2681(1)	5907(1)	5478(1)	26(1)
C(9)	3402(1)	3150(1)	3567(1)	21(1)
C(10)	2997(1)	2003(1)	3316(1)	27(1)
C(11)	3428(1)	1062(1)	3083(1)	31(1)
C(12)	4252(1)	1267(1)	3107(1)	31(1)
C(13)	4655(1)	2410(1)	3370(1)	30(1)
C(14)	4228(1)	3362(1)	3601(1)	26(1)
C(15)	984(1)	3865(1)	4061(1)	23(1)
C(16)	1104(1)	3681(1)	4915(1)	27(1)
C(17)	1140(1)	2494(1)	5264(1)	27(1)
C(18)	1064(1)	1452(1)	4750(1)	25(1)
C(19)	922(1)	1613(1)	3886(1)	29(1)
C(20)	882(1)	2802(1)	3551(1)	26(1)
C(21)	1197(1)	39(1)	5897(1)	30(1)
C(22)	1295(1)	-1343(1)	6061(1)	37(1)

C(23)	977(1)	5157(1)	3680(1)	23(1)
C(24)	531(1)	6139(1)	4027(1)	28(1)
C(25)	551(1)	7423(1)	3678(1)	29(1)
C(26)	1432(1)	7873(1)	3758(1)	30(1)
C(27)	1882(1)	6919(1)	3391(1)	23(1)
C(28)	2777(1)	7348(1)	3473(1)	23(1)
C(29)	3397(1)	7821(1)	4239(1)	27(1)
C(30)	4220(1)	8181(1)	4317(1)	27(1)
C(31)	4445(1)	8075(1)	3612(1)	23(1)
C(32)	3839(1)	7603(1)	2837(1)	25(1)
C(33)	3022(1)	7250(1)	2774(1)	24(1)
C(34)	5835(1)	9037(1)	4373(1)	31(1)
C(35)	6646(1)	9318(2)	4231(1)	48(1)

U(eq) is defined as 1/3 the trace of the Uij tensor.

Table S3. Bond lengths (A) and angles (deg)

S(1)-C(1)	1.648(1)	O(1)-C(18)	1.367(2)
O(1)-C(21)	1.437(2)	O(2)-C(25)	1.214(2)
O(3)-C(31)	1.372(2)	O(3)-C(34)	1.437(2)
N(1)-C(1)	1.353(2)	N(1)-C(4)	1.418(2)
N(1)-C(9)	1.433(2)	C(1)-C(2)	1.540(2)
C(2)-C(3)	1.515(2)	C(2)-C(27)	1.567(2)
C(2)-C(23)	1.568(2)	C(3)-C(8)	1.387(2)
C(3)-C(4)	1.390(2)	C(4)-C(5)	1.382(2)
C(5)-C(6)	1.394(2)	C(5)-H(5)	0.9500
C(6)-C(7)	1.382(2)	C(6)-H(6)	0.9500
C(7)-C(8)	1.391(2)	C(7)-H(7)	0.9500
C(8)-H(8)	0.9500	C(9)-C(14)	1.382(2)
C(9)-C(10)	1.388(2)	C(10)-C(11)	1.386(2)
C(10)-H(10)	0.9500	C(11)-C(12)	1.382(2)
C(11)-H(11)	0.9500	C(12)-C(13)	1.385(2)
C(12)-H(12)	0.9500	C(13)-C(14)	1.390(2)
C(13)-H(13)	0.9500	C(14)-H(14)	0.9500
C(15)-C(16)	1.389(2)	C(15)-C(20)	1.398(2)
C(15)-C(23)	1.525(2)	C(16)-C(17)	1.394(2)
C(16)-H(16)	0.9500	C(17)-C(18)	1.390(2)
C(17)-H(17)	0.9500	C(18)-C(19)	1.392(2)
C(19)-C(20)	1.386(2)	C(19)-H(19)	0.9500
C(20)-H(20)	0.9500	C(21)-C(22)	1.505(2)
C(21)-H(21A)	0.9900	C(21)-H(21B)	0.9900
C(22)-H(22A)	0.9800	C(22)-H(22B)	0.9800
C(22)-H(22C)	0.9800	C(23)-C(24)	1.537(2)
C(23)-H(23)	1.0000	C(24)-C(25)	1.503(2)
C(24)-H(24A)	0.9900	C(24)-H(24B)	0.9900
C(25)-C(26)	1.508(2)	C(26)-C(27)	1.540(2)

C(26)-H(26A)	0.9900	C(26)-H(26B)	0.9900
C(27)-C(28)	1.522(2)	C(27)-H(27)	1.0000
C(28)-C(29)	1.393(2)	C(28)-C(33)	1.399(2)
C(29)-C(30)	1.388(2)	C(29)-H(29)	0.9500
C(30)-C(31)	1.390(2)	C(30)-H(30)	0.9500
C(31)-C(32)	1.393(2)	C(32)-C(33)	1.383(2)
C(32)-H(32)	0.9500	C(33)-H(33)	0.9500
C(34)-C(35)	1.501(2)	C(34)-H(34A)	0.9900
C(34)-H(34B)	0.9900	C(35)-H(35A)	0.9800
C(35)-H(35B)	0.9800	C(35)-H(35C)	0.9800

C(18)-O(1)-C(21)	117.6(1)	C(31)-O(3)-C(34)	117.3(1)
C(1)-N(1)-C(4)	111.9(1)	C(1)-N(1)-C(9)	124.3(1)
C(4)-N(1)-C(9)	123.6(1)	N(1)-C(1)-C(2)	108.0(1)
N(1)-C(1)-S(1)	126.0(1)	C(2)-C(1)-S(1)	126.0(1)
C(3)-C(2)-C(1)	102.3(1)	C(3)-C(2)-C(27)	114.5(1)
C(1)-C(2)-C(27)	107.2(1)	C(3)-C(2)-C(23)	112.9(1)
C(1)-C(2)-C(23)	107.8(1)	C(27)-C(2)-C(23)	111.4(1)
C(8)-C(3)-C(4)	118.6(1)	C(8)-C(3)-C(2)	132.8(1)
C(4)-C(3)-C(2)	108.6(1)	C(5)-C(4)-C(3)	123.3(1)
C(5)-C(4)-N(1)	127.4(1)	C(3)-C(4)-N(1)	109.2(1)
C(4)-C(5)-C(6)	116.9(1)	C(4)-C(5)-H(5)	121.5
C(6)-C(5)-H(5)	121.5	C(7)-C(6)-C(5)	121.0(1)
C(7)-C(6)-H(6)	119.5	C(5)-C(6)-H(6)	119.5
C(6)-C(7)-C(8)	120.8(1)	C(6)-C(7)-H(7)	119.6
C(8)-C(7)-H(7)	119.6	C(3)-C(8)-C(7)	119.3(1)
C(3)-C(8)-H(8)	120.3	C(7)-C(8)-H(8)	120.3
C(14)-C(9)-C(10)	121.3(1)	C(14)-C(9)-N(1)	119.7(1)
C(10)-C(9)-N(1)	119.0(1)	C(11)-C(10)-C(9)	119.1(1)
C(11)-C(10)-H(10)	120.5	C(9)-C(10)-H(10)	120.5
C(12)-C(11)-C(10)	120.2(1)	C(12)-C(11)-H(11)	119.9

C(10)-C(11)-H(11)	119.9	C(11)-C(12)-C(13)	120.3(1)
C(11)-C(12)-H(12)	119.8	C(13)-C(12)-H(12)	119.8
C(12)-C(13)-C(14)	120.0(1)	C(12)-C(13)-H(13)	120.0
C(14)-C(13)-H(13)	120.0	C(9)-C(14)-C(13)	119.1(1)
C(9)-C(14)-H(14)	120.4	C(13)-C(14)-H(14)	120.4
C(16)-C(15)-C(20)	117.0(1)	C(16)-C(15)-C(23)	122.7(1)
C(20)-C(15)-C(23)	120.2(1)	C(15)-C(16)-C(17)	122.2(1)
C(15)-C(16)-H(16)	118.9	C(17)-C(16)-H(16)	118.9
C(18)-C(17)-C(16)	119.5(1)	C(18)-C(17)-H(17)	120.2
C(16)-C(17)-H(17)	120.2	O(1)-C(18)-C(17)	124.8(1)
O(1)-C(18)-C(19)	116.0(1)	C(17)-C(18)-C(19)	119.3(1)
C(20)-C(19)-C(18)	120.2(1)	C(20)-C(19)-H(19)	119.9
C(18)-C(19)-H(19)	119.9	C(19)-C(20)-C(15)	121.7(1)
C(19)-C(20)-H(20)	119.1	C(15)-C(20)-H(20)	119.1
O(1)-C(21)-C(22)	107.3(1)	O(1)-C(21)-H(21A)	110.3
C(22)-C(21)-H(21A)	110.3	O(1)-C(21)-H(21B)	110.3
C(22)-C(21)-H(21B)	110.3	H(21A)-C(21)-H(21B)	108.5
C(21)-C(22)-H(22A)	109.5	C(21)-C(22)-H(22B)	109.5
H(22A)-C(22)-H(22B)	109.5	C(21)-C(22)-H(22C)	109.5
H(22A)-C(22)-H(22C)	109.5	H(22B)-C(22)-H(22C)	109.5
C(15)-C(23)-C(24)	112.7(1)	C(15)-C(23)-C(2)	112.4(1)
C(24)-C(23)-C(2)	111.9(1)	C(15)-C(23)-H(23)	106.4
C(24)-C(23)-H(23)	106.4	C(2)-C(23)-H(23)	106.4
C(25)-C(24)-C(23)	112.9(1)	C(25)-C(24)-H(24A)	109.0
C(23)-C(24)-H(24A)	109.0	C(25)-C(24)-H(24B)	109.0
C(23)-C(24)-H(24B)	109.0	H(24A)-C(24)-H(24B)	107.8
O(2)-C(25)-C(24)	121.9(1)	O(2)-C(25)-C(26)	122.5(1)
C(24)-C(25)-C(26)	115.6(1)	C(25)-C(26)-C(27)	111.8(1)
C(25)-C(26)-H(26A)	109.3	C(27)-C(26)-H(26A)	109.3
C(25)-C(26)-H(26B)	109.3	C(27)-C(26)-H(26B)	109.3
H(26A)-C(26)-H(26B)	107.9	C(28)-C(27)-C(26)	112.7(1)
C(28)-C(27)-C(2)	112.2(1)	C(26)-C(27)-C(2)	112.0(1)

C(28)-C(27)-H(27)	106.5	C(26)-C(27)-H(27)	106.5
C(2)-C(27)-H(27)	106.5	C(29)-C(28)-C(33)	116.8(1)
C(29)-C(28)-C(27)	122.5(1)	C(33)-C(28)-C(27)	120.7(1)
C(30)-C(29)-C(28)	122.3(1)	C(30)-C(29)-H(29)	118.8
C(28)-C(29)-H(29)	118.8	C(29)-C(30)-C(31)	119.7(1)
C(29)-C(30)-H(30)	120.1	C(31)-C(30)-H(30)	120.1
O(3)-C(31)-C(30)	124.4(1)	O(3)-C(31)-C(32)	116.3(1)
C(30)-C(31)-C(32)	119.2(1)	C(33)-C(32)-C(31)	120.1(1)
C(33)-C(32)-H(32)	119.9	C(31)-C(32)-H(32)	119.9
C(32)-C(33)-C(28)	121.8(1)	C(32)-C(33)-H(33)	119.1
C(28)-C(33)-H(33)	119.1	O(3)-C(34)-C(35)	107.9(1)
O(3)-C(34)-H(34A)	110.1	C(35)-C(34)-H(34A)	110.1
O(3)-C(34)-H(34B)	110.1	C(35)-C(34)-H(34B)	110.1
H(34A)-C(34)-H(34B)	108.4	C(34)-C(35)-H(35A)	109.5
C(34)-C(35)-H(35B)	109.5	H(35A)-C(35)-H(35B)	109.5
C(34)-C(35)-H(35C)	109.5	H(35A)-C(35)-H(35C)	109.5
H(35B)-C(35)-H(35C)	109.5		

Table S4. Anisotropic displacement parameters (A^2 x 10^3)

atom	U11	U22	U33	U23	U13	U12
S(1)	30(1)	28(1)	17(1)	-1(1)	8(1)	6(1)
O(1)	36(1)	25(1)	32(1)	-2(1)	16(1)	1(1)
O(2)	35(1)	42(1)	34(1)	2(1)	16(1)	17(1)
O(3)	24(1)	31(1)	24(1)	-3(1)	8(1)	1(1)
N(1)	22(1)	22(1)	18(1)	0(1)	8(1)	4(1)
C(1)	22(1)	20(1)	21(1)	1(1)	10(1)	2(1)
C(2)	22(1)	22(1)	19(1)	-1(1)	10(1)	4(1)
C(3)	22(1)	21(1)	20(1)	0(1)	10(1)	-1(1)
C(4)	24(1)	20(1)	19(1)	0(1)	10(1)	-1(1)
C(5)	25(1)	26(1)	24(1)	2(1)	8(1)	2(1)
C(6)	29(1)	35(1)	19(1)	3(1)	6(1)	-4(1)
C(7)	33(1)	39(1)	19(1)	-5(1)	13(1)	-8(1)
C(8)	29(1)	30(1)	24(1)	-5(1)	14(1)	-2(1)
C(9)	25(1)	23(1)	17(1)	1(1)	9(1)	7(1)
C(10)	26(1)	26(1)	28(1)	-1(1)	11(1)	4(1)
C(11)	35(1)	25(1)	30(1)	-3(1)	11(1)	5(1)
C(12)	36(1)	33(1)	24(1)	1(1)	13(1)	15(1)
C(13)	27(1)	40(1)	26(1)	3(1)	14(1)	9(1)
C(14)	27(1)	28(1)	23(1)	1(1)	12(1)	2(1)
C(15)	18(1)	26(1)	26(1)	-3(1)	10(1)	1(1)
C(16)	28(1)	27(1)	27(1)	-6(1)	14(1)	-1(1)
C(17)	28(1)	30(1)	25(1)	-3(1)	12(1)	0(1)
C(18)	20(1)	26(1)	31(1)	-2(1)	12(1)	-1(1)
C(19)	28(1)	28(1)	30(1)	-8(1)	13(1)	-2(1)
C(20)	23(1)	32(1)	24(1)	-5(1)	10(1)	-1(1)
C(21)	33(1)	28(1)	31(1)	0(1)	15(1)	2(1)
C(22)	41(1)	28(1)	40(1)	2(1)	16(1)	4(1)

C(23)	22(1)	26(1)	22(1)	-2(1)	10(1)	3(1)
C(24)	24(1)	31(1)	33(1)	-3(1)	15(1)	5(1)
C(25)	32(1)	32(1)	25(1)	-4(1)	15(1)	10(1)
C(26)	35(1)	24(1)	34(1)	0(1)	18(1)	7(1)
C(27)	26(1)	21(1)	24(1)	0(1)	11(1)	5(1)
C(28)	28(1)	18(1)	24(1)	2(1)	11(1)	5(1)
C(29)	37(1)	26(1)	21(1)	-2(1)	15(1)	1(1)
C(30)	33(1)	26(1)	20(1)	-3(1)	9(1)	0(1)
C(31)	26(1)	20(1)	23(1)	3(1)	10(1)	5(1)
C(32)	29(1)	27(1)	19(1)	0(1)	11(1)	4(1)
C(33)	27(1)	24(1)	20(1)	0(1)	8(1)	3(1)
C(34)	31(1)	31(1)	25(1)	-3(1)	5(1)	1(1)
C(35)	32(1)	62(1)	47(1)	-15(1)	12(1)	-9(1)

The anisotropic displacement factor exponent takes the form 2 pi^2 [h^2a*^2U(11) +...+ 2hka*b*U(12)]

Table S5. Hydrogen Coordinates (A x 10^4) and equivalent isotropic displacement parameters (A^2 x 10^3)

atom	x	y	z	U(eq)
H(5)	4330	3622	5350	31
H(6)	4463	4459	6690	35
H(7)	3441	5860	6765	36
H(8)	2271	6492	5513	32
H(10)	2432	1865	3303	32
H(11)	3157	274	2907	37
H(12)	4542	621	2942	37
H(13)	5224	2543	3391	36
H(14)	4501	4149	3780	31
H(16)	1163	4387	5274	32
H(17)	1216	2398	5849	32
H(19)	853	907	3525	34
H(20)	782	2897	2958	32
H(21A)	673	348	5968	36
H(21B)	1711	485	6312	36
H(22A)	806	-1779	5618	55
H(22B)	1300	-1524	6632	55
H(22C)	1842	-1626	6039	55
H(23)	619	5086	3046	28
H(24A)	-82	5891	3872	34
H(24B)	823	6161	4665	34
H(26A)	1802	8027	4373	36
H(26B)	1364	8671	3443	36
H(27)	1515	6854	2759	28
H(29)	3250	7901	4726	32
H(30)	4628	8497	4850	32
H(32)	3987	7524	2352	29

H(33)	2615	6931	2241	29
H(34A)	5975	8499	4887	37
H(34B)	5574	9820	4469	37
H(35A)	6891	8540	4121	72
H(35B)	7072	9723	4746	72
H(35C)	6504	9874	3732	72

Cartesian coordinates and energies

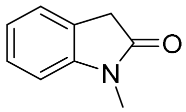

5

E= -478.0935

H= -478.0926

G= -478.1360

C	-2.73524728	-0.38277511	0.00000000
C	-2.43215428	0.99644689	-0.00004000
C	-3.42847928	1.97118089	-0.00001400
C	-4.76673428	1.53679289	-0.00003800
C	-5.08410628	0.17234389	-0.00006100
C	-4.06169028	-0.79792711	-0.00002400
C	-1.43538228	-1.15398511	0.00005700
C	-0.36885828	-0.05418411	-0.00006900
H	-3.18955128	3.02915889	0.00008900
H	-5.56316428	2.27449989	-0.00003700
H	-6.12316528	-0.14064411	-0.00012200
H	-4.31179328	-1.85498611	-0.00002500
H	-1.30270928	-1.79227711	-0.88208900
N	-1.02977328	1.17089189	-0.00012000
C	-0.34289528	2.45432389	0.00003800
H	-0.60192828	3.03462989	0.89331400
H	0.73034772	2.25444589	-0.00035200
H	-0.60256528	3.03512589	-0.89271500
O	0.86798972	-0.19998611	-0.00010500
H	-1.30274128	-1.79172411	0.88265500

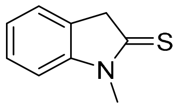

6

E= -801.0530

H= -801.0521

G= -801.0969

C	-0.82905000	-0.93437300	-0.00000800
C	-0.66203400	0.46316300	-0.00000500
C	-1.74312100	1.34300900	-0.00002600
C	-3.03267800	0.78187100	-0.00004700
C	-3.21742000	-0.60770900	-0.00005300
C	-2.10920800	-1.47761900	-0.00003800
C	0.54497500	-1.56450300	0.00002200
C	1.49558800	-0.37034700	0.00009100
H	-1.60654000	2.41872900	-0.00001900
H	-3.89647900	1.43913400	-0.00006100
H	-4.22239900	-1.01703800	-0.00007000
H	-2.25731900	-2.55345200	-0.00004100
H	0.73641800	-2.19085700	-0.87976700
N	0.72686500	0.76541400	0.00001100
C	1.23993900	2.13180100	-0.00004000
H	0.89665500	2.66466700	0.89363600
H	2.32929400	2.08582800	-0.00031400
H	0.89619100	2.66474800	-0.89348700
H	0.73636200	-2.19091200	0.87978300
S	3.19861400	-0.45440700	0.00005500

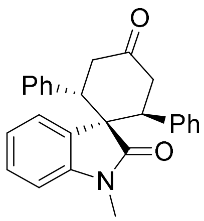

1 (Gas phase)

E= -1203.2183

H= -1203.1979

G= -1203.2079

C	1.48730400	-0.43130500	1.19877400
C	0.03613700	-0.19855400	0.59797500
C	-0.29404800	-1.24325900	-0.51597100
C	-0.11860600	-2.69854400	0.01270200
C	1.27707700	-2.91147800	0.56509500
C	1.64752700	-1.91342400	1.65172600
H	-0.82810500	-2.85480600	0.83134700
H	-0.30442100	-3.41389000	-0.79156300
H	0.94594900	-2.07531100	2.47920300
H	2.67132600	-2.09878400	1.98377200
H	0.44248600	-1.07816700	-1.31199400
C	-0.96512500	-0.30883200	1.77905600
C	-0.18319600	1.23512200	0.13978000
C	0.35293800	1.94747400	-0.92178100
C	-1.12058200	1.85118700	0.98926800
C	-0.04043500	3.28141500	-1.11891600
H	1.07336200	1.48871000	-1.58540000
C	-1.51523600	3.17015200	0.81212900
C	-0.95889600	3.88339400	-0.25823700
H	0.37513200	3.84525400	-1.94515700
H	-2.23774500	3.63458700	1.47101000
H	-1.25382300	4.91375300	-0.41898500
N	-1.55280500	0.93112400	1.96052800
C	-2.56400500	1.17298900	2.99053900
H	-2.66795000	0.23511000	3.54036500
H	-2.24358700	1.96974700	3.66955700
H	-3.52168400	1.44399900	2.53374700
O	2.03299400	-3.81038600	0.17751500
C	-1.68252500	-1.04581800	-1.12077000
C	-2.84949900	-1.36781800	-0.40937000
C	-1.80899100	-0.55669800	-2.42782300
C	-4.10586400	-1.19398100	-0.99234500
H	-2.77756400	-1.75541400	0.59919600
C	-3.06483600	-0.38520000	-3.01267900
H	-0.91550000	-0.30760400	-2.98988000
C	-4.21902300	-0.70273600	-2.29499400
H	-4.99712800	-1.44973900	-0.43025600
H	-3.14036000	-0.00628200	-4.02549300
H	-5.19579800	-0.57359100	-2.74670600
H	1.53796700	0.19335400	2.09954400
C	2.61044200	0.04255300	0.27768900
C	3.19995600	-0.77566900	-0.69906900
C	3.09123100	1.35449400	0.42447000
C	4.21556400	-0.28121800	-1.52319800
H	2.90803300	-1.81163900	-0.79524300
C	4.10878800	1.84579600	-0.39282800
H	2.65647600	1.99535500	1.18312300
C	4.66967200	1.03012800	-1.37856700
H	4.65992700	-0.93151700	-2.26803500
H	4.46418200	2.86109000	-0.25876600
H	5.46086400	1.40814500	-2.01564400
O	-1.21762100	-1.30852000	2.47656400

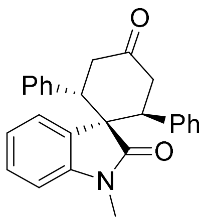

1 (In ethanol)

E= -1195.5054

H= -1195.2395

G= -1195.5004

C	1.48730400	-0.43130500	1.19877400
C	0.03613700	-0.19855400	0.59797500
C	-0.29404800	-1.24325900	-0.51597100
C	-0.11860600	-2.69854400	0.01270200
C	1.27707700	-2.91147800	0.56509500
C	1.64752700	-1.91342400	1.65172600
H	-0.82810500	-2.85480600	0.83134700
H	-0.30442100	-3.41389000	-0.79156300
H	0.94594900	-2.07531100	2.47920300
H	2.67132600	-2.09878400	1.98377200
H	0.44248600	-1.07816700	-1.31199400
C	-0.96512500	-0.30883200	1.77905600
C	-0.18319600	1.23512200	0.13978000
C	0.35293800	1.94747400	-0.92178100
C	-1.12058200	1.85118700	0.98926800
C	-0.04043500	3.28141500	-1.11891600
H	1.07336200	1.48871000	-1.58540000
C	-1.51523600	3.17015200	0.81212900
C	-0.95889600	3.88339400	-0.25823700
H	0.37513200	3.84525400	-1.94515700
H	-2.23774500	3.63458700	1.47101000
H	-1.25382300	4.91375300	-0.41898500
N	-1.55280500	0.93112400	1.96052800
C	-2.56400500	1.17298900	2.99053900
H	-2.66795000	0.23511000	3.54036500
H	-2.24358700	1.96974700	3.66955700
H	-3.52168400	1.44399900	2.53374700
O	2.03299400	-3.81038600	0.17751500
C	-1.68252500	-1.04581800	-1.12077000
C	-2.84949900	-1.36781800	-0.40937000
C	-1.80899100	-0.55669800	-2.42782300
C	-4.10586400	-1.19398100	-0.99234500
H	-2.77756400	-1.75541400	0.59919600
C	-3.06483600	-0.38520000	-3.01267900
H	-0.91550000	-0.30760400	-2.98988000
C	-4.21902300	-0.70273600	-2.29499400
H	-4.99712800	-1.44973900	-0.43025600
H	-3.14036000	-0.00628200	-4.02549300
H	-5.19579800	-0.57359100	-2.74670600
H	1.53796700	0.19335400	2.09954400
C	2.61044200	0.04255300	0.27768900
C	3.19995600	-0.77566900	-0.69906900
C	3.09123100	1.35449400	0.42447000
C	4.21556400	-0.28121800	-1.52319800
H	2.90803300	-1.81163900	-0.79524300
C	4.10878800	1.84579600	-0.39282800
H	2.65647600	1.99535500	1.18312300
C	4.66967200	1.03012800	-1.37856700
H	4.65992700	-0.93151700	-2.26803500
H	4.46418200	2.86109000	-0.25876600
H	5.46086400	1.40814500	-2.01564400
O	-1.21762100	-1.30852000	2.47656400

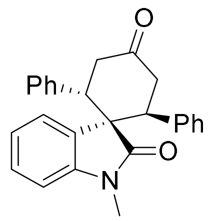

1 (In toluene)

E= -1195.4977

H= -1195.2475

G= -1195.3465

C	-1.14035091	-1.02870812	0.00000000
C	-2.59151791	-0.79595712	-0.60079900
C	-2.92170291	-1.84066212	-1.71474500
C	-2.74626091	-3.29594712	-1.18607200
C	-1.35057791	-3.50888112	-0.63367900
C	-0.98012791	-2.51082712	0.45295200
H	-3.45575991	-3.45220912	-0.36742700
H	-2.93207591	-4.01129312	-1.99033700
H	-1.68170591	-2.67271412	1.28042900
H	0.04367109	-2.69618712	0.78499800
H	-2.18516891	-1.67557012	-2.51076800
C	-3.59277991	-0.90623512	0.58028200
C	-2.81085091	0.63771888	-1.05899400
C	-2.27471691	1.35007088	-2.12055500
C	-3.74823691	1.25378388	-0.20950600
C	-2.66808991	2.68401188	-2.31769000
H	-1.55429291	0.89130688	-2.78417400
C	-4.14289091	2.57274888	-0.38664500
C	-3.58655091	3.28599088	-1.45701100
H	-2.25252291	3.24785088	-3.14393100
H	-4.86539991	3.03718388	0.27223600
H	-3.88147791	4.31634988	-1.61775900
N	-4.18045991	0.33372088	0.76175400
C	-5.19165991	0.57558588	1.79176500
H	-5.29560491	-0.36229312	2.34159100
H	-4.87124191	1.37234388	2.47078300
H	-6.14933891	0.84659588	1.33497300
O	-0.59466091	-4.40778912	-1.02125900
C	-4.31017991	-1.64322112	-2.31954400
C	-5.47715391	-1.96522112	-1.60814400
C	-4.43664591	-1.15410112	-3.62659700
C	-6.73351891	-1.79138412	-2.19111900
H	-5.40521891	-2.35281712	-0.59957800
C	-5.69249091	-0.98260312	-4.21145300
H	-3.54315491	-0.90500712	-4.18865400
C	-6.84667791	-1.30013912	-3.49376800
H	-7.62478291	-2.04714212	-1.62903000
H	-5.76801491	-0.60368512	-5.22426700
H	-7.82345291	-1.17099412	-3.94548000
H	-1.08968791	-0.40404912	0.90077000
C	-0.01721291	-0.55485012	-0.92108500
C	0.57230109	-1.37307212	-1.89784300
C	0.46357609	0.75709088	-0.77430400
C	1.58790909	-0.87862112	-2.72197200
H	0.28037809	-2.40904212	-1.99401700
C	1.48113309	1.24839288	-1.59160200
H	0.02882109	1.39795188	-0.01565100
C	2.04201709	0.43272488	-2.57734100
H	2.03227209	-1.52892012	-3.46680900
H	1.83652709	2.26368688	-1.45754000
H	2.83320909	0.81074188	-3.21441800
O	-3.84527591	-1.90592312	1.27779000

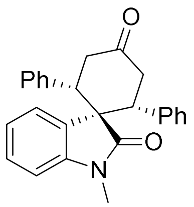

2 (Gas phase)

E= -1203.2089

H= -1203.1794

G= -1203.1945

C	-0.79744819	0.14354067	0.00000000
C	0.49025781	-0.63944933	0.43822900
C	1.77796081	0.14354367	-0.00000600
C	1.75176381	1.61107967	0.52007600
C	0.49025281	2.32361767	0.06242400
C	-0.77125519	1.61107667	0.52008000
H	-0.76504619	0.17604467	-1.09730300
H	1.74667981	1.58857267	1.61499900
H	2.63048081	2.14920867	0.15722500
H	-0.76616719	1.58857067	1.61500400
H	-1.64997419	2.14920267	0.15723200
H	1.74555481	0.17604767	-1.09730800
C	0.49026481	-0.87427133	1.96265600
C	0.49025881	-2.05709433	-0.10372700
C	0.49025381	-2.53124433	-1.40385200
C	0.49026681	-2.95822033	0.97483000
C	0.49025681	-3.91861833	-1.62521700
H	0.49024781	-1.84606933	-2.24473300
C	0.49026981	-4.33147333	0.77440300
C	0.49026481	-4.80323133	-0.54584300
H	0.49025281	-4.30209133	-2.63828200
H	0.49027681	-5.02243733	1.60766800
H	0.49026781	-5.87178833	-0.72587400
N	0.49027081	-2.24014833	2.18416400
C	0.49028181	-2.81615733	3.52896700
H	0.49026981	-1.97200933	4.22190500
H	-0.40492219	-3.42647333	3.68620500
H	1.38550781	-3.42644433	3.68620300
O	0.49025081	3.36861467	-0.59332100
C	-2.08941419	-0.56531733	0.40078800
C	-2.54607219	-0.57399733	1.72873300
C	-2.86173119	-1.20639033	-0.57709700
C	-3.74190019	-1.21228933	2.06246300
H	-1.96522119	-0.08865233	2.50302500
C	-4.05883319	-1.84279033	-0.24458300
H	-2.51986819	-1.20803133	-1.60620500
C	-4.50332819	-1.84714633	1.07848300
H	-4.08184919	-1.20793333	3.09205400
H	-4.64196219	-2.33140033	-1.01673800
H	-5.43414319	-2.33696633	1.34016200
C	3.06992781	-0.56531033	0.40078200
C	3.52657881	-0.57400033	1.72872900
C	3.84225281	-1.20637333	-0.57710400
C	4.72240781	-1.21229033	2.06246000
H	2.94572181	-0.08866533	2.50302300
C	5.03935481	-1.84277033	-0.24459000
H	3.50039381	-1.20800633	-1.60621500
C	5.48384381	-1.84713533	1.07847800
H	5.06235181	-1.20794233	3.09205200
H	5.62249081	-2.33137133	-1.01674700
H	6.41465981	-2.33695333	1.34015700
O	0.49026981	-0.01204033	2.86215900

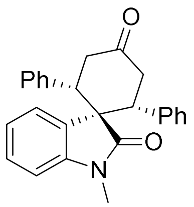

2 (In ethanol)

E= -1195.5118

H= -1195.4754

G= -1195.4916

C	-2.02721283	0.78009693	0.44317295
C	-0.73950683	-0.00289307	0.88140195
C	0.54819617	0.78009993	0.44316695
C	0.52199917	2.24763593	0.96324895
C	-0.73951183	2.96017393	0.50559695
C	-2.00101983	2.24763293	0.96325295
H	-1.99481083	0.81260093	-0.65413005
H	0.51691517	2.22512893	2.05817195
H	1.40071617	2.78576493	0.60039795
H	-1.99593183	2.22512693	2.05817695
H	-2.87973883	2.78575893	0.60040495
H	0.51579017	0.81260393	-0.65413505
C	-0.73949983	-0.23771507	2.40582895
C	-0.73950483	-1.42053807	0.33944595
C	-0.73951083	-1.89468807	-0.96067905
C	-0.73949783	-2.32166407	1.41800295
C	-0.73950783	-3.28206207	-1.18204405
H	-0.73951683	-1.20951307	-1.80156005
C	-0.73949483	-3.69491707	1.21757595
C	-0.73949983	-4.16667507	-0.10267005
H	-0.73951183	-3.66553507	-2.19510905
H	-0.73948783	-4.38588107	2.05084095
H	-0.73949683	-5.23523207	-0.28270105
N	-0.73949383	-1.60359207	2.62733695
C	-0.73948283	-2.17960107	3.97213995
H	-0.73949483	-1.33545307	4.66507795
H	-1.63468683	-2.78991707	4.12937795
H	0.15574317	-2.78988807	4.12937595
O	-0.73951383	4.00517093	-0.15014805
C	-3.31917883	0.07123893	0.84396095
C	-3.77583683	0.06255893	2.17190595
C	-4.09149583	-0.56983407	-0.13392405
C	-4.97166483	-0.57573307	2.50563595
H	-3.19498583	0.54790393	2.94619795
C	-5.28859783	-1.20623407	0.19858995
H	-3.74963283	-0.57147507	-1.16303205
C	-5.73309283	-1.21059007	1.52165595
H	-5.31161383	-0.57137707	3.53522695
H	-5.87172683	-1.69484407	-0.57356505
H	-6.66390783	-1.70041007	1.78333495
C	1.84016317	0.07124593	0.84395495
C	2.29681417	0.06255593	2.17190195
C	2.61248817	-0.56981707	-0.13393105
C	3.49264317	-0.57573407	2.50563295
H	1.71595717	0.54789093	2.94619595
C	3.80959017	-1.20621407	0.19858295
H	2.27062917	-0.57145007	-1.16304205
C	4.25407917	-1.21057907	1.52165095
H	3.83258717	-0.57138607	3.53522495
H	4.39272617	-1.69481507	-0.57357405
H	5.18489517	-1.70039707	1.78332995
O	-0.73949483	0.62451593	3.30533195

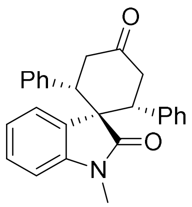

2 (In toluene)

E= -1195.5030

H= -1195.3858

G= -1195.4568

C	-1.99362047	0.88516745	0.00000000
C	-0.70591447	0.10217745	0.43822900
C	0.58178853	0.88517045	-0.00000600
C	0.55559153	2.35270645	0.52007600
C	-0.70591947	3.06524445	0.06242400
C	-1.96742747	2.35270345	0.52008000
H	-1.96121847	0.91767145	-1.09730300
H	0.55050753	2.33019945	1.61499900
H	1.43430853	2.89083545	0.15722500
H	-1.96233947	2.33019745	1.61500400
H	-2.84614647	2.89082945	0.15723200
H	0.54938253	0.91767445	-1.09730800
C	-0.70590747	-0.13264455	1.96265600
C	-0.70591247	-1.31546755	-0.10372700
C	-0.70591847	-1.78961755	-1.40385200
C	-0.70590547	-2.21659355	0.97483000
C	-0.70591547	-3.17699155	-1.62521700
H	-0.70592447	-1.10444255	-2.24473300
C	-0.70590247	-3.58984655	0.77440300
C	-0.70590747	-4.06160455	-0.54584300
H	-0.70591947	-3.56046455	-2.63828200
H	-0.70589547	-4.28081055	1.60766800
H	-0.70590447	-5.13016155	-0.72587400
N	-0.70590147	-1.49852155	2.18416400
C	-0.70589047	-2.07453055	3.52896700
H	-0.70590247	-1.23038255	4.22190500
H	-1.60109447	-2.68484655	3.68620500
H	0.18933553	-2.68481755	3.68620300
O	-0.70592147	4.11024145	-0.59332100
C	-3.28558647	0.17630945	0.40078800
C	-3.74224447	0.16762945	1.72873300
C	-4.05790347	-0.46476355	-0.57709700
C	-4.93807247	-0.47066255	2.06246300
H	-3.16139347	0.65297445	2.50302500
C	-5.25500547	-1.10116355	-0.24458300
H	-3.71604047	-0.46640455	-1.60620500
C	-5.69950047	-1.10551955	1.07848300
H	-5.27802147	-0.46630655	3.09205400
H	-5.83813447	-1.58977355	-1.01673800
H	-6.63031547	-1.59533955	1.34016200
C	1.87375553	0.17631645	0.40078200
C	2.33040653	0.16762645	1.72872900
C	2.64608053	-0.46474655	-0.57710400
C	3.52623553	-0.47066355	2.06246000
H	1.74954953	0.65296145	2.50302300
C	3.84318253	-1.10114355	-0.24459000
H	2.30422153	-0.46637955	-1.60621500
C	4.28767153	-1.10550855	1.07847800
H	3.86617953	-0.46631555	3.09205200
H	4.42631853	-1.58974455	-1.01674700
H	5.21848753	-1.59532655	1.34015700
O	-0.70590247	0.72958645	2.86215900

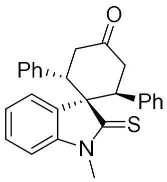

3 (Gas phase)

E= -1524.6162

H= -1524.5954

G= -1524.6064

C	-1.17224883	0.34290271	0.00000000
C	-2.59640083	0.83908171	-0.51238500
C	-3.00421683	0.12602871	-1.86045200
C	-2.89249483	-1.42133229	-1.77052700
C	-1.48053283	-1.81178429	-1.38485100
C	-1.03689583	-1.21007029	-0.06348800
H	-3.58177483	-1.78928429	-1.00288900
H	-3.13854483	-1.86238029	-2.73957900
H	-1.69224683	-1.62664129	0.71166800
H	-0.00114683	-1.49537829	0.13492200
H	-2.25268583	0.46539171	-2.58454000
C	-3.65275883	0.64091571	0.60827500
C	-2.66827483	2.35118371	-0.73832600
C	-1.99587783	3.18362471	-1.62363800
C	-3.66856083	2.88563171	0.08134500
C	-2.32434183	4.54765771	-1.65659300
H	-1.22183383	2.79575471	-2.27084900
C	-4.01092683	4.23157471	0.06431800
C	-3.31722783	5.06432371	-0.82145200
H	-1.79858183	5.20558371	-2.33782300
H	-4.78996083	4.62652171	0.70300700
H	-3.56007983	6.11953671	-0.85971200
N	-4.22498683	1.84786171	0.86783500
C	-5.30098183	2.08549871	1.84096200
H	-5.50997483	1.13732271	2.33613900
H	-4.97756783	2.83053471	2.57417800
H	-6.19374283	2.44285971	1.31853600
S	-4.05037483	-0.80621829	1.44393200
O	-0.76254683	-2.54245229	-2.07882800
C	-4.36871183	0.56018071	-2.39121900
C	-5.56646883	0.09339671	-1.82807100
C	-4.44019783	1.41588271	-3.49961400
C	-6.79889383	0.48295971	-2.35630100
H	-5.53823583	-0.57129729	-0.97414100
C	-5.67141383	1.80467171	-4.02980700
H	-3.52316583	1.78246571	-3.94703000
C	-6.85677083	1.33887871	-3.45835900
H	-7.71403583	0.10974671	-1.91036700
H	-5.70349983	2.46604871	-4.88808200
H	-7.81469183	1.63470971	-3.87044000
H	-1.12583583	0.62764571	1.05731700
C	-0.00404683	1.03951471	-0.69743900
C	0.56104317	0.56243871	-1.89075500
C	0.54340917	2.19402471	-0.11578800
C	1.61892917	1.24500971	-2.49886400
H	0.21452317	-0.36363529	-2.32850600
C	1.60499317	2.86995171	-0.71666800
H	0.12670617	2.56819171	0.81293300
C	2.14071417	2.40245771	-1.91934800
H	2.04374117	0.85843071	-3.41825200
H	2.01303217	3.75794571	-0.24762400
H	2.96499217	2.92549671	-2.39020400

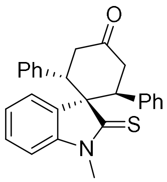

3 (In ethanol)

E= -1516.6051

H= -1516.5684

G= -1516.5894

C	-1.40350881	2.63955339	0.00000000
C	-2.82766081	3.13573239	-0.51238500
C	-3.23547681	2.42267939	-1.86045200
C	-3.12375481	0.87531839	-1.77052700
C	-1.71179281	0.48486639	-1.38485100
C	-1.26815581	1.08658039	-0.06348800
H	-3.81303481	0.50736639	-1.00288900
H	-3.36980481	0.43427039	-2.73957900
H	-1.92350681	0.67000939	0.71166800
H	-0.23240681	0.80127239	0.13492200
H	-2.48394581	2.76204239	-2.58454000
C	-3.88401881	2.93756639	0.60827500
C	-2.89953481	4.64783439	-0.73832600
C	-2.22713781	5.48027539	-1.62363800
C	-3.89982081	5.18228239	0.08134500
C	-2.55560181	6.84430839	-1.65659300
H	-1.45309381	5.09240539	-2.27084900
C	-4.24218681	6.52822539	0.06431800
C	-3.54848781	7.36097439	-0.82145200
H	-2.02984181	7.50223439	-2.33782300
H	-5.02122081	6.92317239	0.70300700
H	-3.79133981	8.41618739	-0.85971200
N	-4.45624681	4.14451239	0.86783500
C	-5.53224181	4.38214939	1.84096200
H	-5.74123481	3.43397339	2.33613900
H	-5.20882781	5.12718539	2.57417800
H	-6.42500281	4.73951039	1.31853600
S	-4.28163481	1.49043239	1.44393200
O	-0.99380681	-0.24580161	-2.07882800
C	-4.59997181	2.85683139	-2.39121900
C	-5.79772881	2.39004739	-1.82807100
C	-4.67145781	3.71253339	-3.49961400
C	-7.03015381	2.77961039	-2.35630100
H	-5.76949581	1.72535339	-0.97414100
C	-5.90267381	4.10132239	-4.02980700
H	-3.75442581	4.07911639	-3.94703000
C	-7.08803081	3.63552939	-3.45835900
H	-7.94529581	2.40639739	-1.91036700
H	-5.93475981	4.76269939	-4.88808200
H	-8.04595181	3.93136039	-3.87044000
H	-1.35709581	2.92429639	1.05731700
C	-0.23530681	3.33616539	-0.69743900
C	0.32978319	2.85908939	-1.89075500
C	0.31214919	4.49067539	-0.11578800
C	1.38766919	3.54166039	-2.49886400
H	-0.01673681	1.93301539	-2.32850600
C	1.37373319	5.16660239	-0.71666800
H	-0.10455381	4.86484239	0.81293300
C	1.90945419	4.69910839	-1.91934800
H	1.81248119	3.15508139	-3.41825200
H	1.78177219	6.05459639	-0.24762400
H	2.73373219	5.22214739	-2.39020400

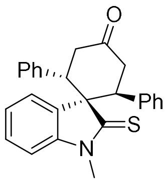

3 (In toluene)

E= -1516.5969

H= -1516.5634

G= -1516.5846

C	-2.94258381	1.24401912	0.00000000
C	-4.36673581	1.74019812	-0.51238500
C	-4.77455181	1.02714512	-1.86045200
C	-4.66282981	-0.52021588	-1.77052700
C	-3.25086781	-0.91066788	-1.38485100
C	-2.80723081	-0.30895388	-0.06348800
H	-5.35210981	-0.88816788	-1.00288900
H	-4.90887981	-0.96126388	-2.73957900
H	-3.46258181	-0.72552488	0.71166800
H	-1.77148181	-0.59426188	0.13492200
H	-4.02302081	1.36650812	-2.58454000
C	-5.42309381	1.54203212	0.60827500
C	-4.43860981	3.25230012	-0.73832600
C	-3.76621281	4.08474112	-1.62363800
C	-5.43889581	3.78674812	0.08134500
C	-4.09467681	5.44877412	-1.65659300
H	-2.99216881	3.69687112	-2.27084900
C	-5.78126181	5.13269112	0.06431800
C	-5.08756281	5.96544012	-0.82145200
H	-3.56891681	6.10670012	-2.33782300
H	-6.56029581	5.52763812	0.70300700
H	-5.33041481	7.02065312	-0.85971200
N	-5.99532181	2.74897812	0.86783500
C	-7.07131681	2.98661512	1.84096200
H	-7.28030981	2.03843912	2.33613900
H	-6.74790281	3.73165112	2.57417800
H	-7.96407781	3.34397612	1.31853600
S	-5.82070981	0.09489812	1.44393200
O	-2.53288181	-1.64133588	-2.07882800
C	-6.13904681	1.46129712	-2.39121900
C	-7.33680381	0.99451312	-1.82807100
C	-6.21053281	2.31699912	-3.49961400
C	-8.56922881	1.38407612	-2.35630100
H	-7.30857081	0.32981912	-0.97414100
C	-7.44174881	2.70578812	-4.02980700
H	-5.29350081	2.68358212	-3.94703000
C	-8.62710581	2.23999512	-3.45835900
H	-9.48437081	1.01086312	-1.91036700
H	-7.47383481	3.36716512	-4.88808200
H	-9.58502681	2.53582612	-3.87044000
H	-2.89617081	1.52876212	1.05731700
C	-1.77438181	1.94063112	-0.69743900
C	-1.20929181	1.46355512	-1.89075500
C	-1.22692581	3.09514112	-0.11578800
C	-0.15140581	2.14612612	-2.49886400
H	-1.55581181	0.53748112	-2.32850600
C	-0.16534181	3.77106812	-0.71666800
H	-1.64362881	3.46930812	0.81293300
C	0.37037919	3.30357412	-1.91934800
H	0.27340619	1.75954712	-3.41825200
H	0.24269719	4.65906212	-0.24762400
H	1.19465719	3.82661312	-2.39020400

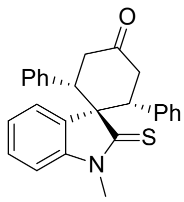

4(Gas phase)

E= -1524.6243

H= -1524.6045

G= -1524.6184

C	-1.28317300	1.10360200	-0.83749800
C	-0.00001800	0.37978800	-0.26899000
C	1.28312000	1.10363000	-0.83751200
C	1.26996200	2.63260200	-0.56440900
C	-0.00005000	3.27672000	-1.09927600
C	-1.27004500	2.63257500	-0.56439600
H	-1.20132800	0.97260200	-1.92516000
H	1.30603500	2.80231800	0.51769100
H	2.13594700	3.09762700	-1.04228600
H	-1.30611100	2.80229200	0.51770400
H	-2.13604400	3.09758200	-1.04226700
H	1.20126700	0.97262700	-1.92517300
C	0.00000500	0.23972500	1.26961000
C	0.00000300	-1.07713300	-0.72006500
C	-0.00000900	-1.64104900	-1.98598600
C	0.00005000	-1.90160400	0.40826400
C	0.00002900	-3.03981200	-2.10576000
H	-0.00004600	-1.01901300	-2.87403200
C	0.00009200	-3.28672200	0.31507900
C	0.00008000	-3.84967200	-0.96756600
H	0.00002000	-3.49425700	-3.08905000
H	0.00013600	-3.91506000	1.19585700
H	0.00011100	-4.92777700	-1.07374100
N	0.00004800	-1.08778000	1.56849000
C	0.00010000	-1.65108600	2.92652700
H	-0.00006300	-0.81625000	3.62727800
H	-0.89559700	-2.26312200	3.06951200
H	0.89599800	-2.26281600	3.06957800
S	-0.00000400	1.49390700	2.44631100
O	-0.00006400	4.23927100	-1.87155500
C	-2.59574400	0.44805900	-0.41275700
C	-3.15599700	0.64857600	0.85818800
C	-3.28878500	-0.35798300	-1.32802500
C	-4.36844700	0.04776000	1.20277500
H	-2.64089400	1.26445400	1.58323900
C	-4.50204900	-0.95676900	-0.98583500
H	-2.87194900	-0.51911400	-2.31595800
C	-5.04617700	-0.75624100	0.28398300
H	-4.78580500	0.21463400	2.18941900
H	-5.02104400	-1.57465700	-1.70968500
H	-5.99006800	-1.21646700	0.55262200
C	2.59570700	0.44811200	-0.41278000
C	3.15596900	0.64864800	0.85815800
C	3.28875200	-0.35792600	-1.32804800
C	4.36843100	0.04785300	1.20273900
H	2.64086500	1.26452500	1.58320900
C	4.50202900	-0.95669100	-0.98586500
H	2.87190900	-0.51907200	-2.31597500
C	5.04616500	-0.75614500	0.28394700
H	4.78579600	0.21474100	2.18937800
H	5.02102700	-1.57457600	-1.70971500
H	5.99006500	-1.21635600	0.55258200

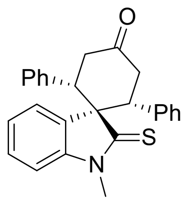

4 (In ethanol)

E= -1516.6100

H= -1516.5794

G= -1516.6034

C	0.78947370	1.90590109	0.00000000
C	2.07262870	1.18208709	0.56850800
C	3.35576670	1.90592909	-0.00001400
C	3.34260870	3.43490109	0.27308900
C	2.07259670	4.07901909	-0.26177800
C	0.80260170	3.43487409	0.27310200
H	0.87131870	1.77490109	-1.08766200
H	3.37868170	3.60461709	1.35518900
H	4.20859370	3.89992609	-0.20478800
H	0.76653570	3.60459109	1.35520200
H	-0.06339730	3.89988109	-0.20476900
H	3.27391370	1.77492609	-1.08767500
C	2.07265170	1.04202409	2.10710800
C	2.07264970	-0.27483391	0.11743300
C	2.07263770	-0.83874991	-1.14848800
C	2.07269670	-1.09930491	1.24576200
C	2.07267570	-2.23751291	-1.26826200
H	2.07260070	-0.21671391	-2.03653400
C	2.07273870	-2.48442291	1.15257700
C	2.07272670	-3.04737291	-0.13006800
H	2.07266670	-2.69195791	-2.25155200
H	2.07278270	-3.11276091	2.03335500
H	2.07275770	-4.12547791	-0.23624300
N	2.07269470	-0.28548091	2.40598800
C	2.07274670	-0.84878691	3.76402500
H	2.07258370	-0.01395091	4.46477600
H	1.17704970	-1.46082291	3.90701000
H	2.96864470	-1.46051691	3.90707600
S	2.07264270	2.29620609	3.28380900
O	2.07258270	5.04157009	-1.03405700
C	-0.52309730	1.25035809	0.42474100
C	-1.08335030	1.45087509	1.69568600
C	-1.21613830	0.44431609	-0.49052700
C	-2.29580030	0.85005909	2.04027300
H	-0.56824730	2.06675309	2.42073700
C	-2.42940230	-0.15446991	-0.14833700
H	-0.79930230	0.28318509	-1.47846000
C	-2.97353030	0.04605809	1.12148100
H	-2.71315830	1.01693309	3.02691700
H	-2.94839730	-0.77235791	-0.87218700
H	-3.91742130	-0.41416791	1.39012000
C	4.66835370	1.25041109	0.42471800
C	5.22861570	1.45094709	1.69565600
C	5.36139870	0.44437309	-0.49055000
C	6.44107770	0.85015209	2.04023700
H	4.71351170	2.06682409	2.42070700
C	6.57467570	-0.15439191	-0.14836700
H	4.94455570	0.28322709	-1.47847700
C	7.11881170	0.04615409	1.12144500
H	6.85844270	1.01704009	3.02687600
H	7.09367370	-0.77227691	-0.87221700
H	8.06271170	-0.41405691	1.39008000

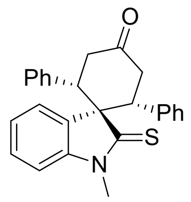

4 (In toluene)

E= -1516.6007

H= -1516.5784

G= -1516.5937

C	-4.45362269	-2.11963501	0.09782903
C	-3.17046769	-2.84344901	0.66633703
C	-1.88732969	-2.11960701	0.09781503
C	-1.90048769	-0.59063501	0.37091803
C	-3.17049969	0.05348299	-0.16394897
C	-4.44049469	-0.59066201	0.37093103
H	-4.37177769	-2.25063501	-0.98983297
H	-1.86441469	-0.42091901	1.45301803
H	-1.03450269	-0.12561001	-0.10695897
H	-4.47656069	-0.42094501	1.45303103
H	-5.30649369	-0.12565501	-0.10693997
H	-1.96918269	-2.25061001	-0.98984597
C	-3.17044469	-2.98351201	2.20493703
C	-3.17044669	-4.30037001	0.21526203
C	-3.17045869	-4.86428601	-1.05065897
C	-3.17039969	-5.12484101	1.34359103
C	-3.17042069	-6.26304901	-1.17043297
H	-3.17049569	-4.24225001	-1.93870497
C	-3.17035769	-6.50995901	1.25040603
C	-3.17036969	-7.07290901	-0.03223897
H	-3.17042969	-6.71749401	-2.15372297
H	-3.17031369	-7.13829701	2.13118403
H	-3.17033869	-8.15101401	-0.13841397
N	-3.17040169	-4.31101701	2.50381703
C	-3.17034969	-4.87432301	3.86185403
H	-3.17051269	-4.03948701	4.56260503
H	-4.06604669	-5.48635901	4.00483903
H	-2.27445169	-5.48605301	4.00490503
S	-3.17045369	-1.72933001	3.38163803
O	-3.17051369	1.01603399	-0.93622797
C	-5.76619369	-2.77517801	0.52257003
C	-6.32644669	-2.57466101	1.79351503
C	-6.45923469	-3.58122001	-0.39269797
C	-7.53889669	-3.17547701	2.13810203
H	-5.81134369	-1.95878301	2.51856603
C	-7.67249869	-4.18000601	-0.05050797
H	-6.04239869	-3.74235101	-1.38063097
C	-8.21662669	-3.97947801	1.21931003
H	-7.95625469	-3.00860301	3.12474603
H	-8.19149369	-4.79789401	-0.77435797
H	-9.16051769	-4.43970401	1.48794903
C	-0.57474269	-2.77512501	0.52254703
C	-0.01448069	-2.57458901	1.79348503
C	0.11830231	-3.58116301	-0.39272097
C	1.19798131	-3.17538401	2.13806603
H	-0.52958469	-1.95871201	2.51853603
C	1.33157931	-4.17992801	-0.05053797
H	-0.29854069	-3.74230901	-1.38064797
C	1.87571531	-3.97938201	1.21927403
H	1.61534631	-3.00849601	3.12470503
H	1.85057731	-4.79781301	-0.77438797
H	2.81961531	-4.43959301	1.48790903

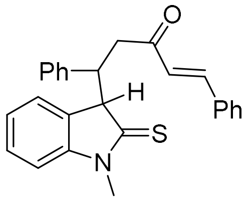

E= -1524.164265

H= -1524.163321

G= -1524.247851

C	-4.75279119	0.03189793	0.00000000
C	-5.22291419	-1.09307907	1.00834300
C	-0.88068319	0.03357293	0.42513300
C	-2.25736119	-0.43529407	0.16437100
C	-3.36485119	0.58747893	0.38161600
H	-4.62767019	-0.50160807	-0.94911000
H	-0.75832719	1.06258693	0.74309700
H	-3.34912019	0.88694793	1.43876700
H	-3.14396119	1.47967993	-0.22006200
C	-5.70982019	-0.54929607	2.35320000
C	-6.44795919	-1.81096907	0.47484200
C	-6.63791619	-2.57584007	-0.66422500
C	-7.51814219	-1.57004307	1.34697100
C	-7.91748919	-3.09415007	-0.92358000
H	-5.81214019	-2.77846907	-1.33669100
C	-8.79182919	-2.06723507	1.10543200
C	-8.97829919	-2.83633607	-0.05170800
H	-8.08261619	-3.70058207	-1.80597900
H	-9.61507219	-1.87459307	1.78097500
H	-9.96089819	-3.23941207	-0.26650100
N	-7.04628319	-0.80946107	2.44852000
C	-7.90591519	-0.40800207	3.56924900
H	-7.27675419	0.11828293	4.28809300
H	-8.69880919	0.25339093	3.20679200
H	-8.34722119	-1.29404907	4.03654300
S	-4.75560319	0.21316593	3.55334300
O	-2.50187919	-1.60383307	-0.19987800
C	-5.80242419	1.11764893	-0.19731400
C	-5.94166819	2.18947293	0.69877500
C	-6.65245719	1.06428793	-1.31161900
C	-6.90645519	3.17581793	0.48333200
H	-5.30960719	2.24318093	1.57596700
C	-7.61683019	2.05002793	-1.52851800
H	-6.55982419	0.23935393	-2.00888700
C	-7.74599219	3.11146793	-0.63076400
H	-6.99932319	3.99617293	1.18628100
H	-8.26254719	1.98999793	-2.39728600
H	-8.49061519	3.88122093	-0.79872300
C	0.15157781	-0.81680507	0.27749800
H	-0.11062219	-1.82418007	-0.04332200
H	-4.37426319	-1.76840407	1.14046400
C	1.57331381	-0.55702607	0.49810400
C	2.49312881	-1.59964407	0.27419100
C	2.06703581	0.69029693	0.93000900
C	3.85860681	-1.40537907	0.47311800
H	2.12422781	-2.56456307	-0.05664900
C	3.43025981	0.88313493	1.12858100
H	1.37824581	1.50642393	1.11244600
C	4.33172281	-0.16317007	0.90094300
H	4.55155881	-2.21955207	0.29643700
H	3.79482781	1.84752093	1.46263400
H	5.39287281	-0.00918607	1.05781200

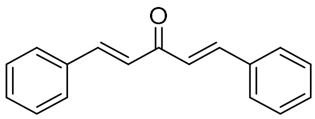

E= -730.994723

H= -730.993779

G= -731.055450

C	-7.36842125	0.19138756	0.00000000
H	-7.28216425	1.27465356	-0.00008000
C	-6.11123925	-0.58247344	-0.00014700
C	-4.85405725	0.19138756	0.00000100
H	-4.94031425	1.27465356	-0.00007700
C	-3.65129725	-0.42655444	0.00021700
H	-3.67530725	-1.51587444	0.00027800
C	-8.57118125	-0.42655444	0.00021600
H	-8.54717125	-1.51587444	0.00027900
C	-2.32322925	0.18601456	0.00036900
C	-2.11358625	1.58331456	0.00031100
C	-1.19100825	-0.65715244	0.00058500
C	-0.82294025	2.11075656	0.00046500
H	-2.96398525	2.25699056	0.00014700
C	0.10166275	-0.12869544	0.00073900
H	-1.33668725	-1.73347144	0.00063200
C	0.29062075	1.25785956	0.00068000
H	-0.68089025	3.18683156	0.00041800
H	0.95804675	-0.79528944	0.00090600
H	1.29369175	1.67216256	0.00079900
C	-9.89924925	0.18601456	0.00036700
C	-11.03147025	-0.65715244	0.00058700
C	-10.10889225	1.58331456	0.00030600
C	-12.32414125	-0.12869544	0.00074000
H	-10.88579125	-1.73347144	0.00063600
C	-11.39953825	2.11075656	0.00045900
H	-9.25849325	2.25699056	0.00014000
C	-12.51309925	1.25785956	0.00067700
H	-13.18052525	-0.79528944	0.00090900
H	-11.54158825	3.18683156	0.00041000
H	-13.51617025	1.67216256	0.00079600
O	-6.11123925	-1.84389044	0.00028000

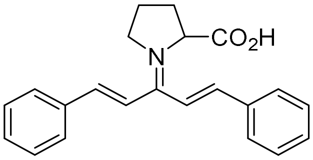

E= -1043.6002

H= -1043.5798

G= -1043.5964

C	-1.47527915	-1.22807016	0.00000000
H	-1.69823315	-2.09816916	-0.58601200
C	-2.58751415	-0.28569116	0.09871300
C	-3.90763715	-0.95472616	0.03148900
H	-3.99194015	-1.70064016	-0.73448700
C	-4.85729115	-0.79270516	0.94100100
H	-4.68757215	-0.11152016	1.75535200
C	-0.34149115	-1.12188316	0.69556300
H	-0.19926015	-0.28299716	1.35987300
C	-6.14368015	-1.51124816	0.98725600
C	-6.70048015	-2.12088616	-0.13362900
C	-6.82909915	-1.57458916	2.19576400
C	-7.90366315	-2.79130516	-0.04040200
H	-6.20557115	-2.05565416	-1.08187600
C	-8.03218015	-2.24935616	2.28945000
H	-6.41491115	-1.10063416	3.06443500
C	-8.57113315	-2.86062816	1.17202300
H	-8.32471215	-3.25253916	-0.91123100
H	-8.54532215	-2.29508616	3.22886800

H	-9.50555015	-3.38053816	1.24124300
C	0.77845785	-2.06752716	0.71043000
C	1.85550085	-1.73590316	1.52967000
C	0.82947185	-3.24182616	-0.03681500
C	2.96143085	-2.56317116	1.60094800
H	1.80680885	-0.82610116	2.09754100
C	1.93307685	-4.06588516	0.03684300
H	0.01116885	-3.51363116	-0.67322700
C	3.00081985	-3.72764916	0.85621700
H	3.78473985	-2.29939616	2.23374400
H	1.96581685	-4.96881816	-0.53961700
H	3.85639985	-4.37120216	0.91051800
N	-2.47408315	0.98882584	0.23537300
C	-3.58893315	1.99411084	0.26713800
C	-1.19744515	1.78517284	0.33967100
H	-3.61995315	2.36322984	1.27960400
H	-4.51433615	1.55210984	-0.05061800
C	-3.04745915	3.07682284	-0.66961000
C	-1.54605215	3.14972684	-0.30122800
H	-0.40520715	1.25267484	-0.15255200
C	-0.92577815	1.94955184	1.89483900
H	-3.55147015	4.01943184	-0.51174500
H	-3.17853215	2.76930184	-1.69954400
H	-1.39351015	3.91263384	0.44369200
H	-0.93300615	3.34700584	-1.16908100
O	-1.72150615	2.71442484	2.46011400
O	0.01197385	1.25799284	2.34913500
